# A biomimetic nanoparticle coordinates antibacterial and immunomodulatory functions to promote periodontal regeneration

**DOI:** 10.1038/s41368-026-00459-y

**Published:** 2026-10-26

**Authors:** Yufei Tang, Junhua Xu, Fang Zheng, Qin Xue, Sze Wing Cheung, Min Sun, Song Zhou, Yao He, Lin Xiang

**Affiliations:** 1 State Key Laboratory of Oral Diseases & National Center for Stomatology & National Clinical Research Center for Oral Diseases, West China Hospital of Stomatology, Sichuan University, Chengdu, China; 2 Biopharmaceutical Research Institute, West China Hospital, Sichuan University, Chengdu, China; 3 Stomatological Hospital of Chongqing Medical University & Chongqing Key Laboratory of Oral Diseases and Biomedical Sciences & Chongqing Municipal Key Laboratory of Oral Biomedical Engineering of Higher Education, Chongqing, China

**Keywords:** Periodontitis, Nanoparticles

## Abstract

Periodontitis is a chronic inflammatory disorder driven by pathogenic microbial communities and an imbalanced host immune response, finally leading to progressive breakdown of periodontal soft and hard tissues. Current treatments primarily target infection but often fail to resolve inflammation or support subsequent tissue repair. Hence, we developed NMm@MINO, a biomimetic nanoparticle constructed by coating minocycline-loaded gelatin nanoparticles with a hybrid neutrophil-macrophage membrane. NMm@MINO formed a stable core-shell structure with a hydrodynamic diameter of (261.53 ± 11.59) nm and a membrane shell thickness of ~21 nm, maintaining colloidal stability for 30 days. Minocycline release was protease-responsive, with ~50% payload released within 2 h under gelatinase exposure and reaching equilibrium at ~8 h. In vitro, NMm@MINO achieved > 99% killing efficiency against *Porphyromonas gingivalis* and *Staphylococcus aureus*. Simultaneously, NMm@MINO reprogrammed polarization by downregulating M1-associated transcripts (*Nos2, Tnf, Il1b, and Cd86*) and upregulating M2-associated markers (*Mrc1, Arg1, and Tgfb1*), corroborated by decreased iNOS and increased CD206 immunofluorescence. NMm@MINO further enhanced osteogenesis (upregulating *Alpl, Spp1, Runx2*), suppressed RANKL-induced osteoclastogenesis (reduced TRAP staining and downregulated *Acp5, Tnfrsf11a, Ctsk, Fos, and Mmp9*; decreased c-Fos immunofluorescence), and promoted fibroblast migration with increased Collagen I immunofluorescence. In a ligature-induced rat periodontitis model (days 3, 7, and 14), local NMm@MINO reduced inflammation (IL-6/CRP IHC; decreased *Il6, Crp, and Il1b*), improved collagen remodeling (H&E/Masson; increased *Col1a1*), and mitigated alveolar bone loss (micro-CT: CEJ-ABC, BV/TV, Tb.N, Tb.Th, and Tb.Sp) while modulating osteoclast/osteogenic markers (TRAP, RANKL/OPG IHC; *Acp5, Tnfsf11, Tnfrsf11b, and Runx2*). Collectively, NMm@MINO coordinates antibacterial, immunomodulatory, and pro-regenerative actions for periodontitis therapy.

## Introduction

Periodontitis is one of the most common oral diseases, which spreads worldwide, affecting over 1 billion people.^1,2^ It is actually a multifactorial disease, driven by the interaction between pathogenic bacteria and a disordered host immune response, and finally leads to the progressive destruction of both periodontal hard and soft tissues.^3,4^ The initiating factor of this disease is a bacterial infection, with *Porphyromonas gingivalis* (*P. gingivalis*) being a key pathogen, whose presence in the periodontal pocket induces chronic inflammation.^5,6^ Such inflammation will activate immune cells like neutrophils and macrophages in turn, resulting in an overactive immune response that aggravates periodontal tissue damage.^7,8^ Conventional periodontitis therapy, such as scaling and root planing, mainly focuses on antibacterial treatments. While effective in controlling infection, these approaches often fall short in modulating immune response and facilitating tissue regeneration.^2,9^ The complexity of periodontitis requires a more comprehensive approach that integrates antibacterial therapy, immune modulation, and tissue regeneration.^10^ Thus, effective periodontitis treatment processes must not only control infection but also regulate immune homeostasis and finally promote the regeneration of both hard and soft tissues.

Growing insight into the multifactorial pathogenesis of periodontitis is shifting therapeutic strategies toward integrated approaches that concurrently target bacteria, modulate immunity, and promote regeneration, driving the development of advanced delivery systems for periodontitis.^11–15^ Despite their promising outcomes, these strategies still face several limitations. First, many current approaches focus only on certain key biological events in periodontitis, failing to provide a complete combination of antibacterial, immunomodulatory, and pro-regenerative functionalities.^10,16–18^ Second, the efficacy of therapeutics is often compromised by limited drug penetration and retention within periodontal pockets, and insufficient active targeting to inflammatory sites.^19,20^ Third, the body’s immune clearance mechanisms can rapidly eliminate administered drugs, reducing their therapeutic potential.^19^ Finally, under the persistent inflammatory microenvironment characteristic of periodontitis, existing regenerative strategies often fail to pay enough attention to the simultaneous repair of both hard and soft tissues.^10^^,21^^,22^

To address these complex challenges, local nano-drug delivery systems offer a promising approach due to their ability to improve drug availability and prolong its retention at the disease site.^19^^,23^^,24^ Yet, traditional nanoparticles (NPs) are suffering from the rapid clearance of the mononuclear phagocyte system and a lack of active targeting to the inflamed tissue.^25,26^ Inspired by the body’s own cell-cell communication, biomimetic cell membrane-coated nanotechnology created new opportunities.^27^ By leveraging the complex protein native to cell membranes, this approach could endow synthetic nanoparticles with complex biological functions, enabling superior immune evasion and bioactive targeting.^27–29^ Among potential cell sources, neutrophil membranes exhibit an excellent ability to seek out inflammation, providing a guiding signal to direct nanoparticles to diseased periodontal tissue^30^. Beyond inflammation homing, neutrophil membrane-coated nanoparticles have also been reported to act as biological decoys that neutralize pro-inflammatory cytokines and attenuate inflammatory damage in vivo.^31^ Moreover, recent mechanistic evidence indicates that neutrophil membrane-coated nanoparticles can display intrinsic anti-inflammatory activity even without drug payload, including mediator sequestration and downstream modulation of inflammatory immune responses.^30^ These findings support the rationale for employing neutrophil membranes as a functional biointerface in inflammatory periodontal lesions.

Concurrently, macrophage membranes have been reported not only to prolong circulation and provide systemic immunomodulatory signals,^32–34^ but also to offer local regulatory cues within inflamed tissues.^35,36^ However, a membrane from only one type of cell cannot perform all the necessary functions to effectively reach and remain in the inflamed periodontal tissue. Consequently, the innovative concept of a hybrid membrane is able to fuse the proteins from both cell types.^37,38^ This design integrates their complementary functions, potentially yielding a synergistic targeting and therapeutic effect unattainable with single-membrane coatings.^39,40^

Building upon this bioinspired design, we engineered a hybrid membrane vesicle derived from neutrophils and macrophages (NMm) to wrap drug-loaded nanoparticle cores. This NMm coating is designed to integrate the strong attraction of neutrophils with the immunomodulatory potential of macrophage membranes, displaying targeting signals from both cells, which enables dual targeting of the inflamed periodontal pocket. As the drug to be loaded, we selected minocycline (MINO), a broad-spectrum tetracycline antibiotic, serving as a widely utilized therapeutic antibiotic for periodontitis.^41^ In addition to its antibacterial effects, MINO exhibits well-documented immunomodulatory properties, such as inhibiting matrix metalloproteinases (MMP) and mitigating pro-inflammatory responses, which are critical for preventing host-mediated tissue damage.^42,43^ To leverage these properties, we loaded MINO into gelatin nanoparticles (GNPs), a biodegradable and MMP-responsive carrier, to form a drug-loaded core, designated as GNP@MINO. In the inflammatory periodontal environment, elevated MMP levels degrade the GNP matrix, promoting the localized release of MINO.^44^

We therefore hypothesize that the GNP@MINO core with a hybrid neutrophil-macrophage membrane termed NMm@MINO will serve as a comprehensive therapeutic platform for periodontitis treatment. We suggest that the NMm coating will confer excellent immune evasion and inflammation-targeting capabilities, guiding the NPs to the periodontal lesion. Upon arrival, the NPs could release MINO in response to elevated MMP levels at the disease site, not only directly eliminating pathogens but also reprogramming the local immune microenvironment. Furthermore, we anticipate that the hybrid membrane itself and the released MINO may coordinately promote periodontal regeneration by enhancing osteogenesis, suppressing osteoclastogenesis, and stimulating periodontal ligament fibroblast functions. Distinct from prior studies focusing on single functions or single-source membranes, our work leverages a dual-source hybrid membrane platform plus sustained local MINO release to achieve an integrated antibacterial, immunomodulatory, and pro-regenerative outcome.

Here, we engineered NMm@MINO, characterized its physicochemical properties and biosafety, and evaluated antibacterial, immunomodulatory, and pro-regenerative outcomes in vitro and in vivo. We systematically evaluated their biocompatibility, antibacterial efficacy, immunomodulatory functions on macrophages, and impacts on osteogenic, osteoclastic and fibroblastic-related cells. For in vivo assessment, we examined the therapeutic efficacy of NMm@MINO in a rat periodontitis model at 3-, 7-, and 14-days post-treatment to determine its effect across different disease stages, from early inflammation to established tissue destruction. Our findings indicate that NMm@MINO exerts therapeutic benefits throughout the course of periodontitis, demonstrating its potential as a versatile treatment option capable of addressing both initial inflammatory responses and later-stage tissue damage. This capability represents a significant advantage for clinical translation. Overall, this work presents a novel and promising biomimetic strategy for synergistic therapy of complex inflammatory diseases.

## Results

### Successful fabrication and characterization of biomimetic hybrid membrane nanoparticles

**Fig. 1:**
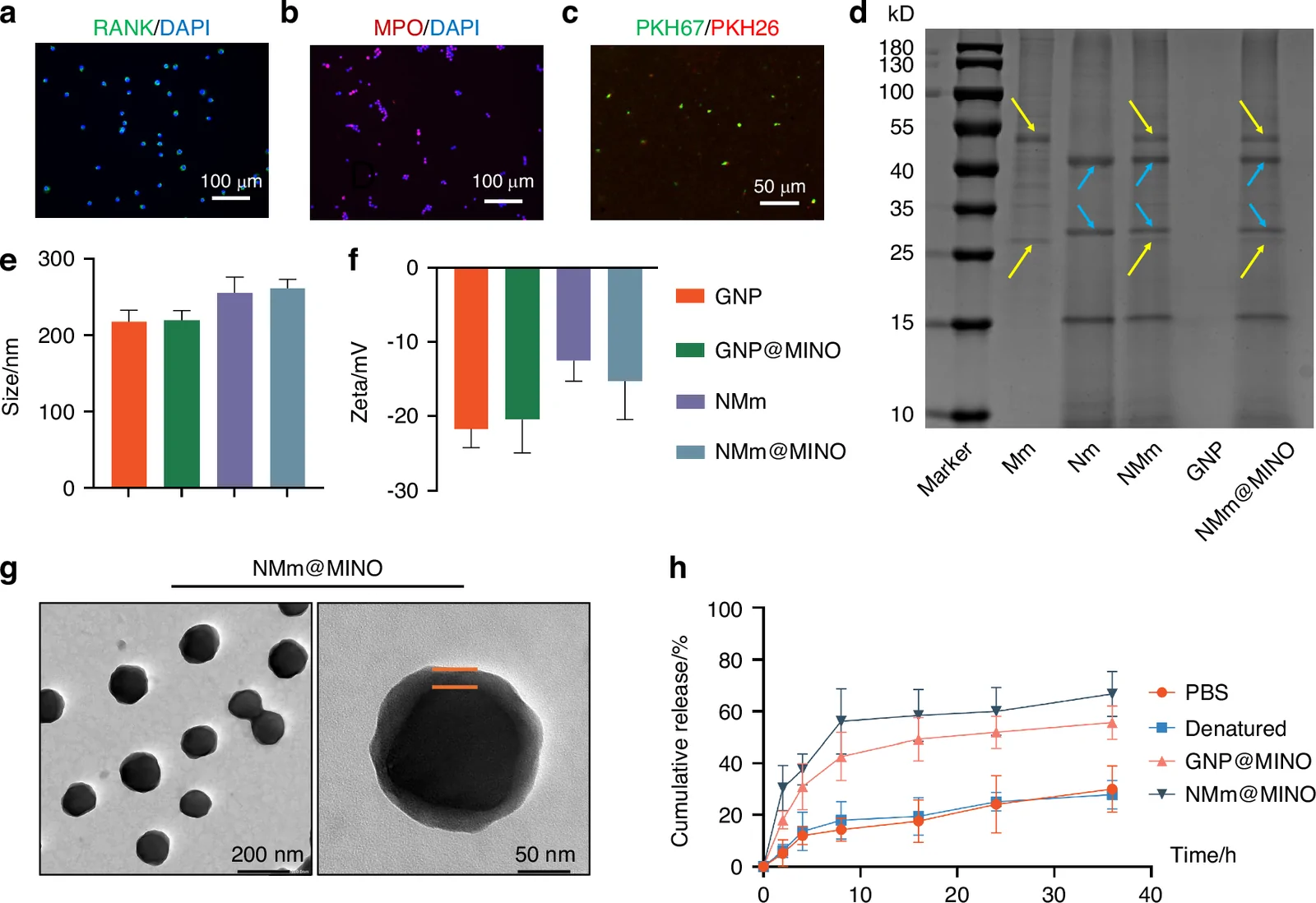
Fabrication and physicochemical characterization of NMm@MINO nanoparticles. **a** Immunofluorescence staining of RANK in NR8383 rat macrophages. Scale bar = 100 μm. **b** Immunofluorescence staining of MPO in primary rat neutrophils. Scale bar = 100 μm. **c** Fluorescence validation of hybrid membrane fusion (neutrophil membrane: PKH67, green; macrophage membrane: PKH26, red). Scale bar = 50 μm. **d** SDS-PAGE protein analysis of Mm, Nm, NMm, GNP and NMm@MINO (yellow arrows indicating the representative protein band of Mm, blue arrows indicating the representative protein band of Nm). **e** Average hydrodynamic diameter of NMm@MINO. **f** Zeta potential of NMm@MINO nanoparticles. **g** Representative TEM images of NMm@MINO. The cell membrane coating, NMm, is outlined by the orange lines. Scale bars = 200 nm (left), 50 nm (right). **h** In vitro release profiles of MINO from GNP@MINO and NMm@MINO. All data are presented as mean ± SD (*n* = 3)

To construct biomimetic nanoparticles with dual-targeting capabilities, we first characterized the key membrane features of the source cells. Immunofluorescence staining confirmed the distinct expression of receptor activator of NF-κB (RANK), a receptor pivotal for immune cell communication, on NR8383 rat macrophage membranes (Fig. 1a). Concurrently, neutrophils were isolated from rat bone marrow via gradient centrifugation, and their identity was verified by the characteristic surface expression of myeloperoxidase (MPO) (Fig. 1b). Subsequently, NMm were prepared using membranes derived from these two cell types. To visually confirm membrane fusion, a critical step for creating a unified nanoparticle coating with combined functionalities—the neutrophil and macrophage membranes were pre-labeled with PKH67 (green) and PKH26 (red) fluorescent dyes, respectively (Figs. S1 and S2). Fluorescence microscopy analysis of the resulting nanoparticles revealed a clear and extensive co-localization of both red and green signals, providing direct evidence for the successful fusion of the two distinct cell membranes into a homogeneous hybrid coating (Fig. 1c). The electrophoretic profiles (Fig. 1d) showed that the fused membrane (NMm) lane contained characteristic protein bands present in both the individual macrophage and neutrophil membrane lanes. Notably, the NMm@MINO lane displayed a similar banding pattern to the fused membrane, confirming the successful coating of the fused cell membrane onto the nanoparticles. The absence of these characteristic bands in the naked gelatin nanoparticle (GNP) control further verified that the protein signals originated from the membrane coating. This result directly confirmed the successful fusion of the two cell membranes and their effective coating on the nanoparticle surface.

The hydrodynamic diameter of NMm@MINO nanoparticles, determined by dynamic light scattering (DLS), was (261.53 ± 11.59) nm (Fig. S3, and Table S1). Compared with uncoated GNPs, the particle size of NMm@MINO increased significantly, accompanied by a decrease in the absolute value of the zeta potential (Fig. 1e, f). This change reflects the successful coating of negatively charged hybrid cell membranes onto the nanoparticle surface, which can partially neutralize the surface charge of the core material. The incorporation of MINO did not markedly alter either particle size or zeta potential, suggesting that drug loading occurred mainly within the nanoparticle matrix rather than affecting its outer surface charge.

Both GNP@MINO and NMm@MINO remained colloidally stable over 30 days in aqueous dispersion (Fig. S4), indicating that the formulations possess inherent physical stability under storage conditions. Transmission electron microscopy (TEM) revealed that GNPs were spherical and well-dispersed, while NMm@MINO nanoparticles displayed a distinct core-shell structure with a uniform shell thickness of approximately 21 nm (Figs. 1g and S5). This morphology provided direct visual confirmation of successful membrane encapsulation. In parallel, minocycline loading conditions were optimized by varying the MINO:GNP feeding ratio. As shown in Fig. S6, increasing the feeding ratio from 20:100 to 200:100 (w/w) led to a gradual decrease in encapsulation efficiency but a plateauing increase in loading capacity; therefore, a ratio of 100:100 (w/w) was selected for all subsequent experiments.

To assess the protease-responsive release behavior of the formulations, NMm@MINO and the corresponding controls were incubated in HEPES buffer supplemented with gelatinase for 36 h, and the cumulative release of MINO was quantified by HPLC. As illustrated in Fig. 1h, exposure to gelatinase markedly accelerated MINO release, with roughly half of the payload liberated within the initial 2 h, followed by a gradual and sustained release phase that reached equilibrium at approximately 8 h.

### NMm@MINO exhibits excellent biocompatibility and hemocompatibility for in vivo application

Prior to evaluating biological efficacy, we first assessed the safety profile of the nanoparticles. CCK-8 assay was employed to determine a suitable working concentration. As illustrated in Fig. S7, a concentration of 80 µg/mL was identified as the minimal dose that consistently promoted the proliferation of RAW264.7 macrophages across 6, 12, and 24 h time points, and was therefore selected for all subsequent in vitro experiments.

**Fig. 2:**
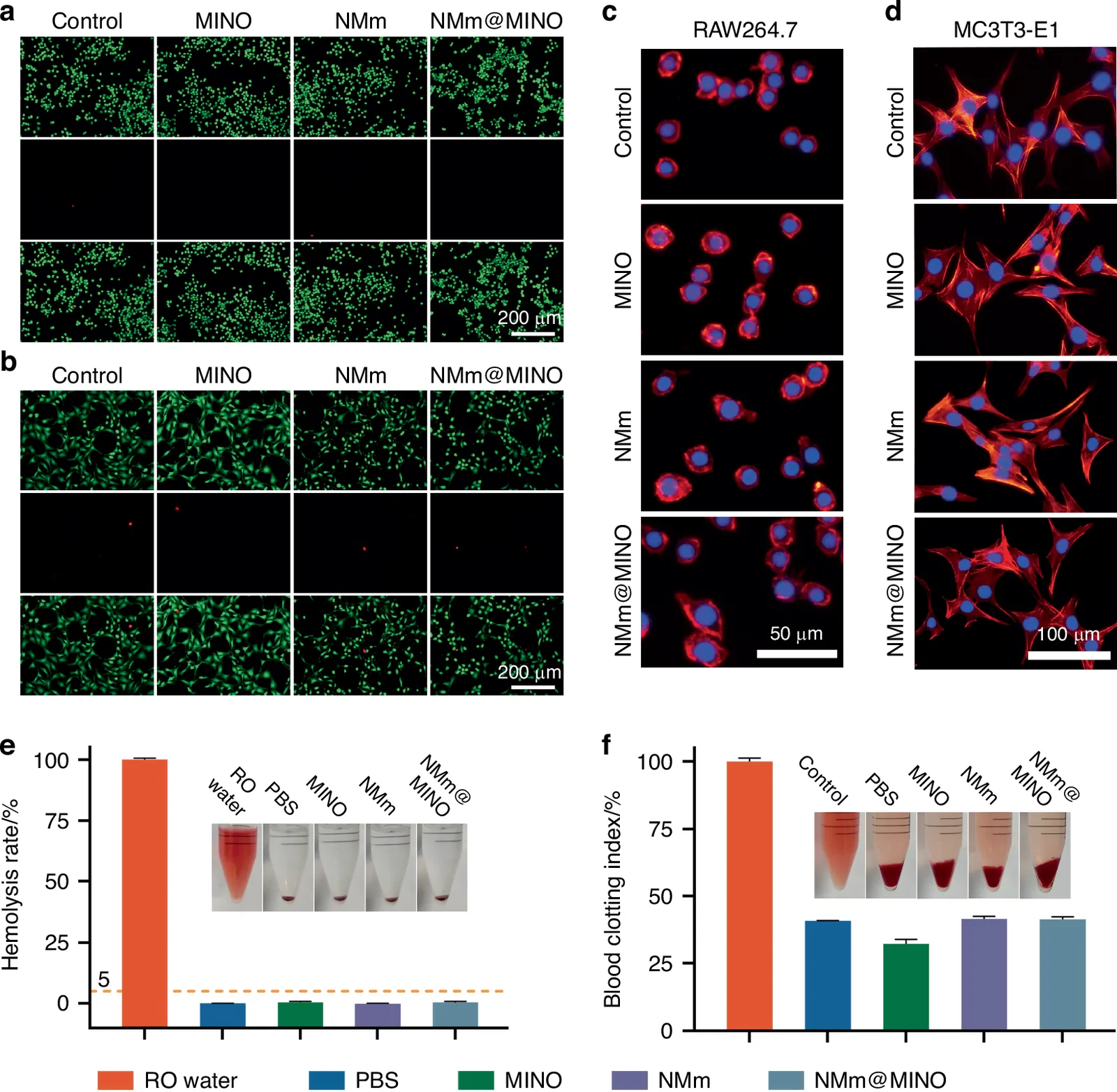
In vitro biocompatibility and hemocompatibility assessment of NMm@MINO nanoparticles. **a** Representative fluorescence images of live/dead staining in RAW264.7 cells. Scale bar = 200 μm. **b** Representative fluorescence images of live/dead staining in MC3T3-E1 cells. Scale bar = 200 μm. **c** Cytoskeleton staining of RAW264.7 cells. Scale bar = 50 μm. **d** Cytoskeleton staining of MC3T3-E1 cells. Scale bar = 100 μm. **e** Hemolysis assay corresponding quantitative hemolysis rate, *n* = 3. **f** Blood coagulation test and clotting index, *n* = 3. Data are presented as mean ± SD

To dissect the contribution of each component, we included four experimental groups: a control group, free MINO at an equivalent concentration to the loaded drug, the hybrid membrane vesicles alone (NMm), and the complete nanoparticle formulation (NMm@MINO). Biocompatibility was evaluated using RAW264.7 macrophages and MC3T3-E1 pre-osteoblasts, which represent crucial cell types involved in periodontitis pathogenesis and bone regeneration. Phase-contrast microscopy revealed no discernible alterations in the morphology of either cell type after treatment (Figs. S8 and S9). This initial observation was further corroborated by live/dead staining (Fig. 2a, b). For RAW264.7 cells, virtually no red fluorescence (indicating dead cells) was detected across all groups. MC3T3-E1 cells exhibited minimal red fluorescence. Then, quantitative analysis of cell death from three random fields confirmed no statistically significant differences among the groups (Fig. S10). These results collectively indicate that neither MINO, the NMm coating, nor the final NMm@MINO formulation exerts any significant cytotoxic effects.

Furthermore, cytoskeleton staining was performed to evaluate effects on cell viability and morphological integrity. Interestingly, treatment with MINO, NMm, and NMm@MINO induced a morphological shift in RAW264.7 cells from their original rounded shape to an elongated, spindle-like morphology (Figs. 2c and S11). In contrast, the morphology and aspect ratio of MC3T3-E1 cells remained unchanged across all treatment groups (Figs. 2d and S11), underscoring the formulation’s safety for osteoblastic cell lineage.

Further hemolysis analysis revealed that the color of all sample groups was comparable to the phosphate buffered saline (PBS) (negative control) group and distinctly different from the bright red solution of reverse osmosis (RO) water (positive control) group, where complete hemolysis occurred (Fig. 2e). Quantification showed that the hemolysis rate for all formulations was well below the 5% ISO safety threshold, confirming their high hemocompatibility. Similarly, in the blood coagulation test, all samples exhibited a similar color to the PBS control and showed no statistically significant difference in the blood clotting index (BCI) (Fig. 2f), indicating that the nanoparticles do not interfere with the coagulation cascade.

### NMm@MINO elicits potent bactericidal effects against *P. gingivalis* by disrupting bacterial membranes and inducing oxidative stress

*Porphyromonas gingivalis* (*P. gingivalis*), a main pathogen in periodontitis, contributes to disease progression by secreting proteases that degrade gingival collagen and evade host immune defenses. We hypothesized that NMm@MINO would provide effective antibacterial activity against *P. gingivalis* through localized sustained release (release data are shown in Fig. 1h) of MINO. Accordingly, colony-forming unit (CFU) assays were performed to quantify antibacterial effects against *P. gingivalis*.

**Fig. 3:**
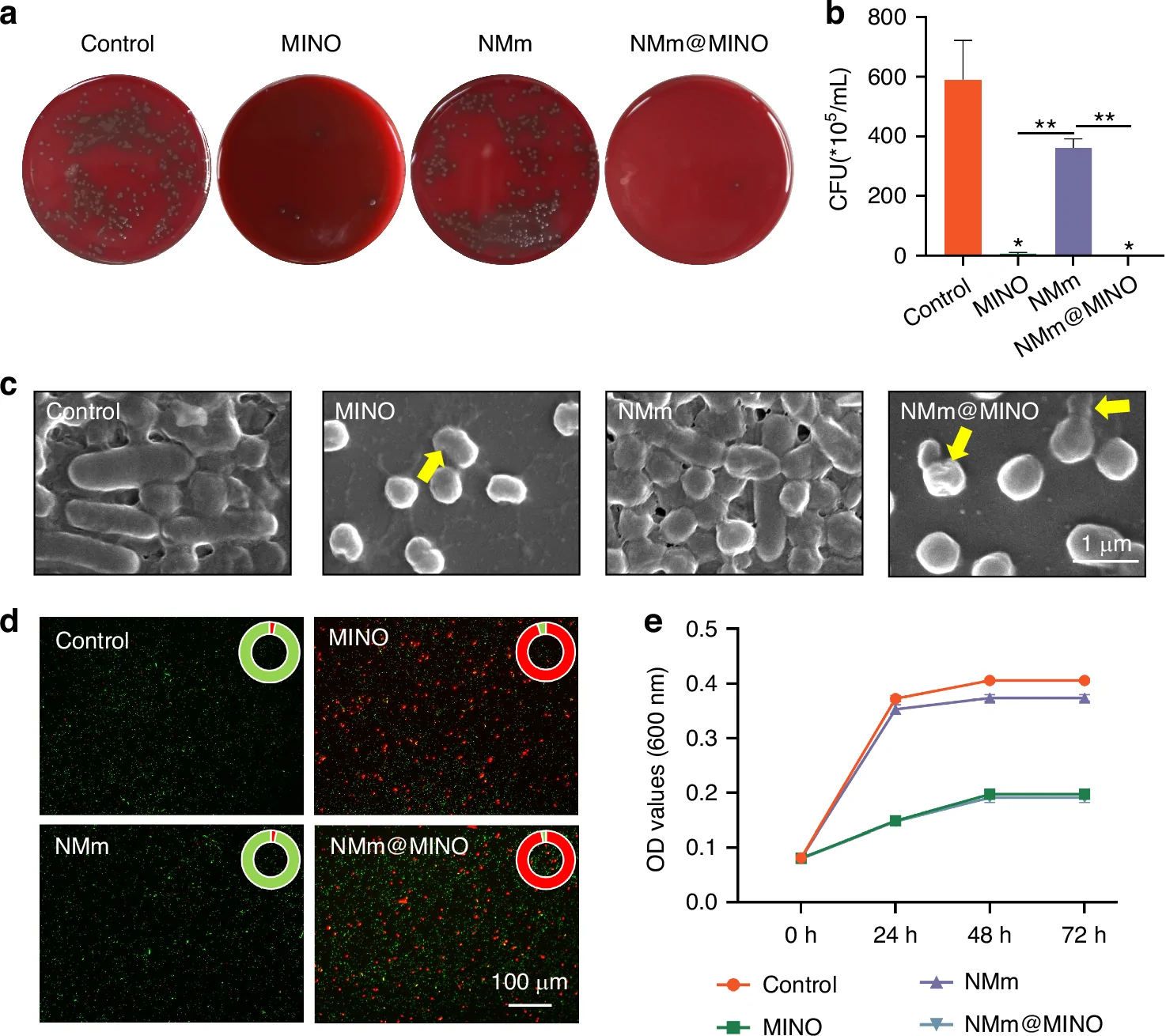
Antibacterial activity effects of NMm@MINO in vitro. **a** Representative images of *P. gingivalis* bacterial colonies. **b** Bacterial colony counting. **c** Representative SEM images of bacteria (yellow arrows indicating wrinkled membranes). Scale bar = 1 μm. **d** Representative live/dead bacterial staining images. Scale bar = 100 μm. **e** Bacterial growth curves (OD_600_ measurement). Data are presented as mean ± SD, *n* = 3, ^*^P < 0.05, ^**^P < 0.01

Initial assessment via CFU assays confirmed this hypothesis. As shown in Fig. 3a, abundant colonies were present in the control and NMm-only groups, whereas both free MINO and NMm@MINO led to a marked reduction in colony numbers. Quantitatively (Fig. 3b), the control group reached ~6 × 10^7^ CFU/mL. The NMm group was not significantly different from control, while both MINO and NMm@MINO reduced viable counts by at least two orders of magnitude.

Further explorations were conducted by scanning electron microscopy (SEM). SEM images (Fig. 3c) revealed a high density of intact bacteria with regular morphology in the control and NMm groups. In contrast, both the MINO and NMm@MINO groups showed a markedly reduced bacterial count, with surviving cells exhibiting membrane wrinkling and concave deformations (indicated by yellow arrows), suggesting structural damage to the cell envelope. This bactericidal effect was corroborated by live/dead bacterial staining (Fig. 3d), where green fluorescence indicates all bacteria and red fluorescence indicates dead cells. The NMm@MINO group displayed the most intense red fluorescence, signifying the highest proportion of dead bacteria, while minimal red signal was detected in the control and NMm groups. To monitor bacterial growth dynamics, we measured the optical density (OD_600_) of cultures over 72 h (Fig. 3e). The control and NMm groups exhibited a characteristic rapid growth phase within the first 24 h, followed by high OD values, indicating robust bacterial proliferation. In contrast, the MINO and NMm@MINO groups showed only marginal increases in OD_600_ over time, confirming potent growth inhibition.

Given the observed membrane disruption, loss of viability, and suppressed proliferation, phenomena often associated with elevated oxidative stress in bacteria, we next examined whether oxidative stress accompanied these effects. Intracellular reactive oxygen species (ROS) staining and semi-quantitative analysis (Fig. S12) revealed the most intense green fluorescence in the MINO and NMm@MINO groups, indicating substantial ROS accumulation and oxidative stress in bacteria exposed to MINO-containing formulations.

*Staphylococcus aureus* (*S. aureus*) is a clinically relevant Gram-positive opportunistic pathogen found in oral infections. Therefore, to evaluate the broader antibacterial potential of NMm@MINO, we tested its efficacy against *S. aureus*, extending the assessment beyond periodontal pathogens. As shown in Fig. S13, the results followed a trend similar to that observed with *P. gingivalis*. Compared to the control, NMm alone had minimal effect on *S. aureus*, whereas both MINO and NMm@MINO groups exhibited a substantial reduction in bacterial colonies, indicating effective bactericidal activity. Furthermore, continuous monitoring of the bacterial culture OD_600_ revealed that, in contrast to the control and NMm groups, the MINO and NMm@MINO groups showed only marginal increases in optical density over time, confirming potent and sustained growth inhibition.

### NMm@MINO reprograms macrophages by balancing M1/M2 polarization under inflammatory conditions

Given the central role of macrophages in orchestrating inflammation and their plasticity between pro-inflammatory M1 and pro-resolving M2 phenotypes, we focused on whether NMm@MINO could favorably regulate macrophage polarization. An inflammatory model was established by stimulating RAW264.7 macrophages with lipopolysaccharide (LPS), with untreated cells as the negative control (control group) and LPS-only treated cells as the positive control (LPS group). All treatment groups were pre-stimulated with LPS before intervention, modeling a resolution after an established inflammation scenario.

**Fig. 4:**
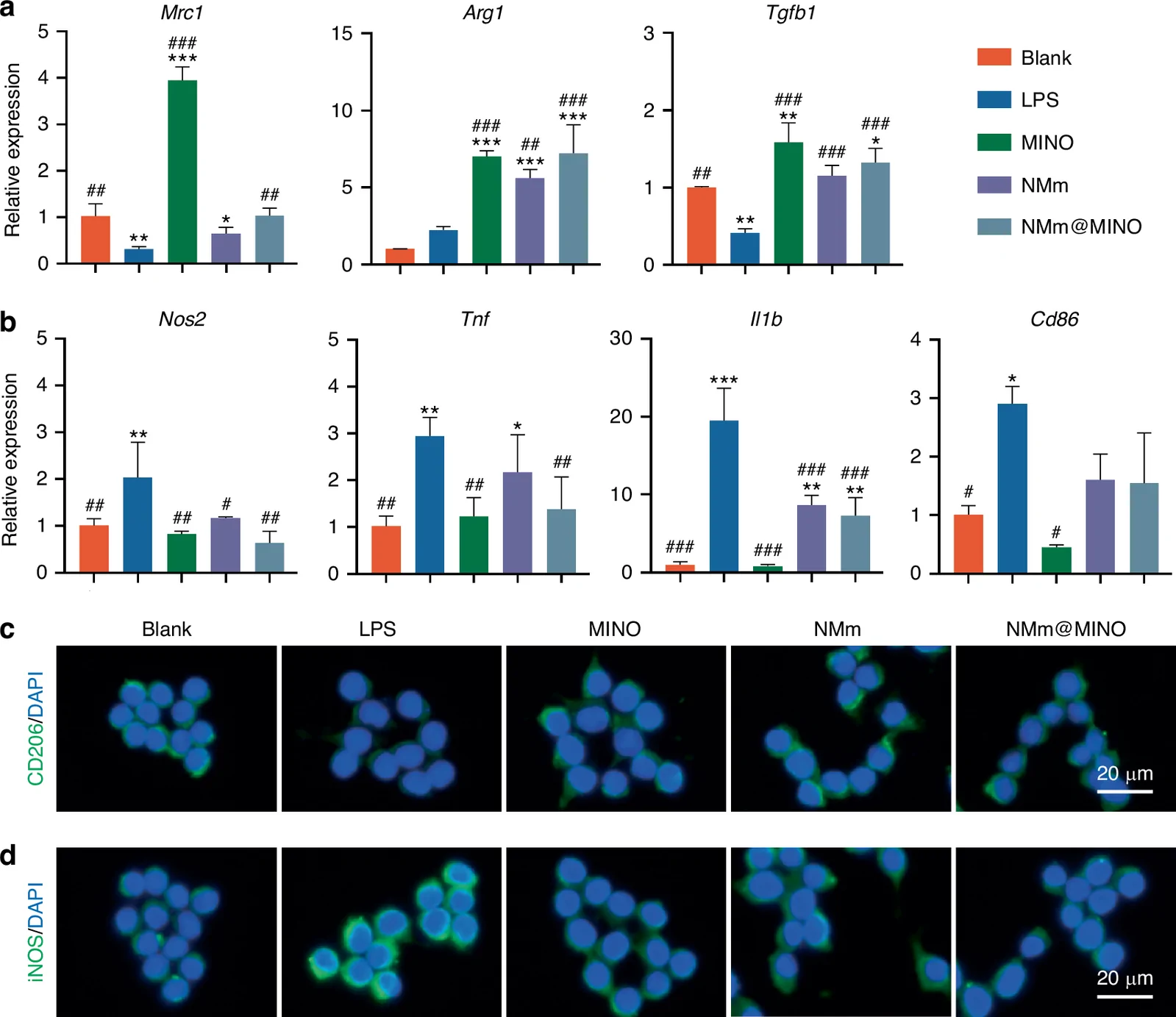
Immunomodulatory effects of NMm@MINO in vitro. **a** mRNA expression of M2 markers (*Mrc1*, *Arg1*, and *Tgfb1*) in RAW264.7 cells. **b** mRNA expression of M1 markers (*Nos2*, *Tnf*, *Il1b*, and *Cd86*) in RAW264.7 cells. **c** CD206 immunofluorescence staining. Scale bar = 20 μm. **d** iNOS immunofluorescence staining. Scale bar = 20 μm. Data are presented as mean ± SD, *n* = 3, compared with control group, ^*^P < 0.05, ^**^P < 0.01, ^***^P < 0.001; compared with LPS group, ^#^*P* < 0.05, ^##^*P* < 0.01, ^###^*P* < 0.001

Real-time PCR analysis revealed that LPS stimulation significantly suppressed the expression of M2 anti-inflammatory markers *Mrc1* (encoding CD206) and *Tgfb1* (encoding TGF-β) (Fig. 4a). This suppression was effectively reversed by both free MINO and NMm@MINO treatments, which not only restored but further enhanced the mRNA expression of *Mrc1*, *Arg1* (encoding Arg-1), and *Tgfb1*. Conversely, LPS stimulation markedly upregulated the mRNA levels of M1 pro-inflammatory markers (*Nos2* (encoding iNOS), *Tnf* (encoding tumor necrosis factor-alpha, TNF-α), *Il1b* (encoding interleukin-1 beta, IL-1β), and *Cd86*) (Fig. 4b). While the NMm coating alone was sufficient to partially normalize *Nos2* and *Cd86* toward baseline, the complete NMm@MINO formulation reduced all four M1 transcripts to levels not different from control, indicating complementary roles of the minocycline payload and the neutrophil-macrophage membrane interface.

Immunofluorescence analysis of CD206 (M2) and iNOS (M1) markers confirmed this phenotypic shift at the protein level (Fig. 4c, d), as further evidenced by the corresponding semi-quantitative data (Figs. S14 and S15). Consistent with the PCR results, the LPS group exhibited the weakest CD206 signal and the strongest iNOS intensity. In contrast, treatments with MINO and NMm@MINO restored the fluorescence intensities of both markers to levels similar to the control group.

Since inflammatory cytokines are key upstream mediators driving macrophage activation and polarization under periodontitis-related inflammatory conditions, we further performed a comparative cytokine neutralization assay to directly verify the “decoy-like” function of the membrane-coated nanoparticles. IL-1β and IL-6, two representative proinflammatory cytokines closely associated with periodontitis, were selected for this assay. Standard IL-1β solution at 300 pg/mL and IL-6 solution at 400 pg/mL were incubated with GNP, Mm@GNP, Nm@GNP, or NMm@GNP, respectively, followed by enzyme-linked immunosorbent assay (ELISA) detection of the residual cytokine concentrations in the supernatant.

As shown in Fig. S16, bare GNP showed negligible neutralization ability toward both IL-1β and IL-6, indicating that the decrease in cytokine levels was not mainly caused by nonspecific adsorption of the nanoparticle core. Compared with GNP, Mm@GNP moderately reduced the residual concentrations of IL-1β and IL-6, suggesting a certain cytokine-binding capacity derived from the macrophage membrane coating. More importantly, Nm@GNP and NMm@GNP markedly decreased the detectable levels of both cytokines, demonstrating a stronger decoy-like cytokine sequestration ability. The comparable cytokine neutralization effects between Nm@GNP and NMm@GNP further indicated that the neutrophil membrane-derived decoy-like function was preserved after fusion with macrophage membrane. Therefore, the comparative design among GNP, single-membrane-coated nanoparticles, and fused-membrane-coated nanoparticles experimentally supports the rationality and effectiveness of incorporating neutrophil membrane into the NMm coating strategy.

### NMm@MINO exerts comprehensive effects on periodontal tissues by coordinating regeneration of hard and soft tissues

An ideal therapeutic agent for periodontitis should not only control infection and inflammation but also directly facilitate the regeneration of the compromised periodontium. To this end, we investigated whether NMm@MINO could directly modulate the core processes governing periodontal tissue homeostasis, specifically focusing on its impact on osteogenic differentiation, osteoclast formation, and the migratory and synthetic functions of periodontal ligament fibroblasts.

**Fig. 5:**
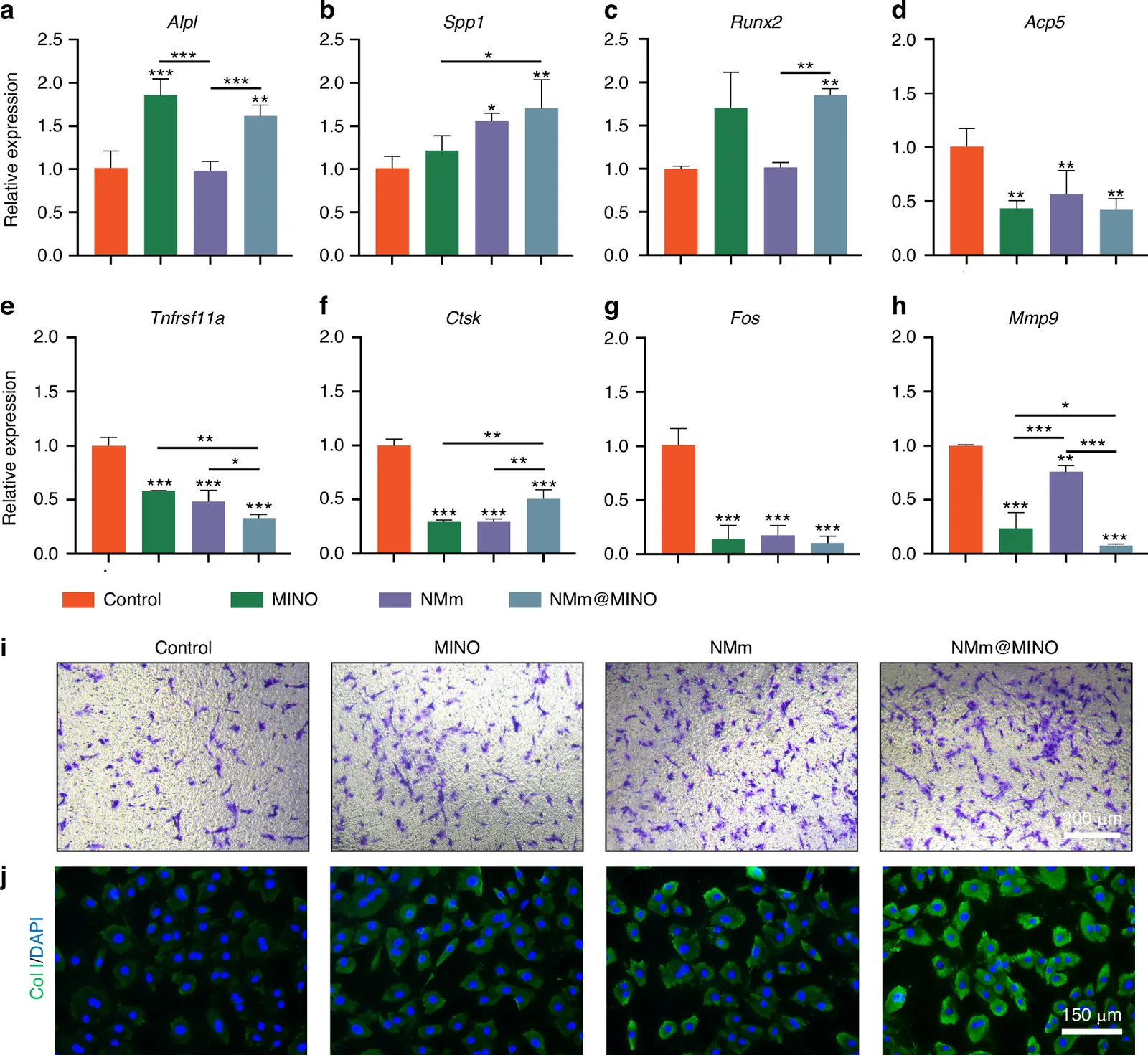
Effects of NMm@MINO on osteogenic, osteoclastic, and fibroblast functions in vitro. mRNA expression of osteogenic markers: **a** *Alpl*, **b** *Spp1*, **c** *Runx2*. mRNA expression of osteoclast-related genes: **d** *Acp5*, **e** *Tnfrsf11a*, **f** *Ctsk*, **g** *Fos*, **h** *Mmp9*. **i** Representative images of the transwell migration assay. Scale bar = 200 μm. **j** Representative immunofluorescence images of Col I expressed by rPDLFs. Scale bar = 150 μm. Data are presented as mean ± SD, *n* = 3, ^*^P < 0.05, ^**^P < 0.01 and ^***^P < 0.001

We first assessed the effect of NMm@MINO on the osteogenic differentiation of MC3T3-E1 pre-osteoblasts by examining both protein activity and mRNA expression levels, using alkaline phosphatase (ALP) staining and qPCR, respectively. ALP staining revealed that the NMm@MINO group exhibited the most intense purplish-blue coloration, indicative of the highest ALP activity, followed by the free MINO group (Fig. S17). Notably, the NMm-only group also showed a slightly darker stain compared to the control group, suggesting a modest pro-osteogenic effect of the hybrid membrane coating itself. Subsequent qPCR analysis of key osteogenic markers further delineated the contributions of each component (Fig. 5a–c). The complete NMm@MINO nanoparticle significantly upregulated the mRNA expression of *Alpl*, *Spp1* (encoding osteopontin, OPN), and *Runx2*. In contrast, free MINO solely enhanced *Alpl* expression, while the NMm coating specifically promoted *Spp1* expression.

Having established the pro-osteogenic effect of NMm@MINO, we next explored whether it could concurrently suppress the bone-resorptive activity of osteoclasts to coordinately favor bone anabolism. Periodontal bone homeostasis is critically dependent on the balance between bone formation by osteoblasts and bone resorption by osteoclasts.

Following the stimulation of RAW264.7 cells with RANKL to induce osteoclastogenesis, osteoclast differentiation was assessed by tartrate-resistant acid phosphatase (TRAP) staining (Fig. S18). In the control group, significant cell fusion, characteristics of mature osteoclasts, was observed. Interestingly, free MINO stimulation appeared to increase the extent of cell fusion and promoted the formation of larger, more extensively branched osteoclasts with darker staining. In contrast, both the NMm and NMm@MINO groups exhibited markedly lighter staining and significantly less cell fusion. qPCR analysis of osteoclast-related genes provided further insight (Fig. 5d–h). Free MINO, NMm, and NMm@MINO all significantly suppressed the expression of *Acp5* (encoding TRAP), *Tnfrsf11a* (encoding RANK), *Ctsk*, *Fos* (encoding c-Fos), and *Mmp9* compared to the control. It is particularly noteworthy that NMm@MINO induced the lowest expression of *Tnfrsf11a* and *Mmp9*. The suppressive effect of c-Fos expression was corroborated at the protein level by immunofluorescence staining (Fig. S19).

In addition to rebalancing bone metabolism, the regeneration of the supportive periodontal soft tissues is paramount. CCK-8 assays revealed that while MINO, NMm, and NMm@MINO all promoted the proliferation of rat periodontal ligament fibroblasts (rPDLFs) to varying degrees on days 1, 3, and 5. NMm and NMm@MINO, particularly the latter, exhibited a stronger pro-proliferative effect on days 1 and 5 (Fig. S20). The transwell migration assay demonstrated that NMm@MINO possessed the most potent ability to stimulate rPDLF migration (Fig. 5i). Furthermore, to assess the cells’ synthetic capacity, we performed immunofluorescence staining for collagen type I (Col I). As shown in Fig. 5j, both MINO and NMm slightly enhanced Col I expression compared to the control, while NMm@MINO treatment resulted in the most intense fluorescence signal among all groups. The coordinated enhancement of proliferation, migration, and matrix synthesis by NMm@MINO highlights its multi-faceted role in promoting soft tissue repair.

### NMm@MINO alleviates periodontal inflammation by suppressing pro-inflammatory mediators

**Fig. 6:**
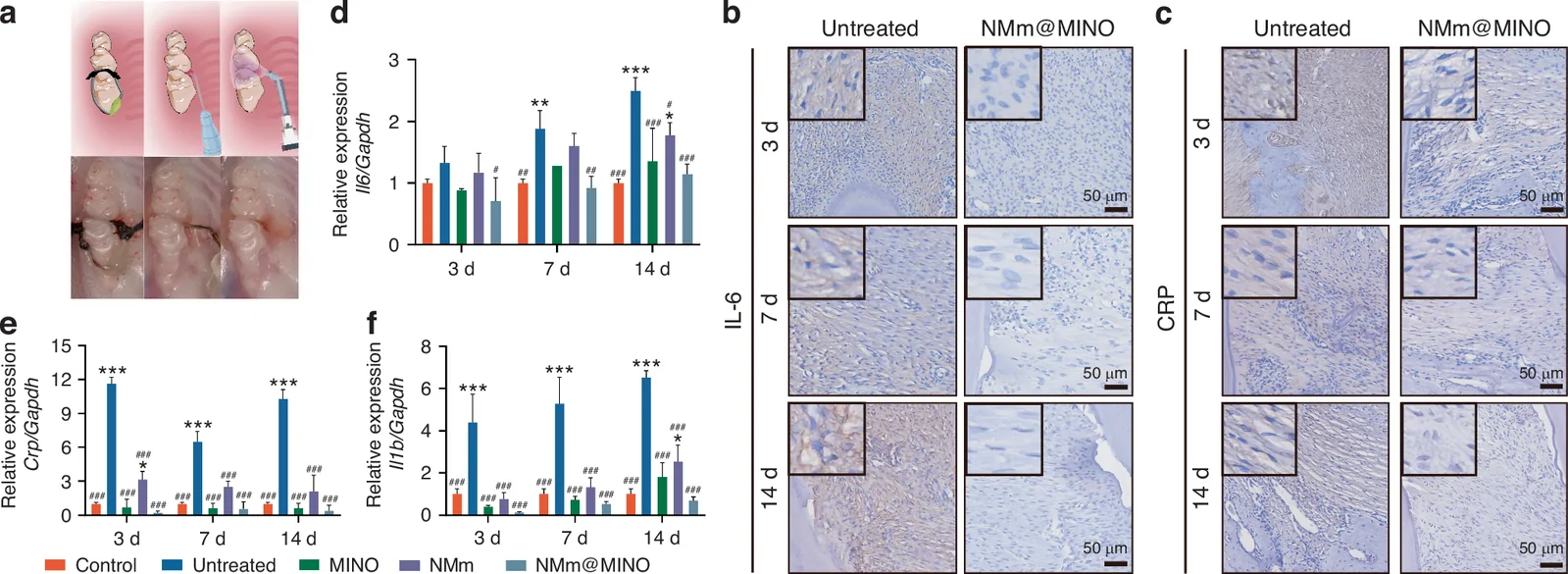
Anti-inflammatory effect of NMm@MINO in vivo. **a** Schematic diagram of the rat periodontitis experiment. Top: Schematic illustrations showing: (i) induction of periodontitis by ligature placement between the maxillary first and second molars; (ii) local administration of therapeutic agents into the periodontal pocket; and (iii) light-curing of the material using a dental curing light. Bottom: Corresponding in vivo photographs of rat maxillary molars depicting the actual ligature placement, local drug delivery, and light-curing steps. **b** IHC staining of IL-6 in the periodontal tissue. Scale bar = 50 μm. **c** IHC staining of CRP in the periodontal tissue. Scale bar = 50 μm. **d** mRNA expression of *Il6*. **e** mRNA expression of *Crp*. **f** mRNA expression of *Il1b*. Data are presented as mean ± SD, *n* = 3, compared with control group, ^*^P < 0.05, ^**^P < 0.01, ^***^P < 0.001; compared with untreated group, ^#^*P* < 0.05, ^##^*P* < 0.01, ^###^*P* < 0.001

To confirm the therapeutic efficacy of NMm@MINO under physiologically relevant conditions, a ligature-induced periodontitis model was established in Sprague-Dawley rats. Ligatures were maintained for 3, 7, and 14 days to simulate early, intermediate, and advanced stages of disease, respectively. Following ligature removal, therapeutic agents were injected locally into the periodontal ligament space every 3 days (Fig. 6a). Five groups were examined: control (healthy, no ligature), untreated (periodontitis, no treatment), MINO (periodontitis + free minocycline), NMm (periodontitis + hybrid membrane vesicles), and NMm@MINO (periodontitis + neutrophil-macrophage hybrid-membrane-coated, minocycline-loaded gelatin nanoparticles).

Prior to in vivo experiments, preliminary studies were performed to optimize local administration frequency. To determine the optimal dosing interval for local injection, we assessed in vivo retention of NMm@MINO at the gingival sites using methylene blue-labeled formulations. Representative gingival images were acquired by 0, 24, 48, and 72 h post-injection (Fig. S21). At 0 h, robust retention of NMm@MINO was confirmed by distinct methylene blue staining at the gingival sites. The blue signal gradually diminished by 24 h, further declined at 48 h, and was nearly undetectable by 72 h, indicating minimal residual drug at the gingival sites. These findings guided the selection of a 3-day (72 h) dosing interval for subsequent therapeutic experiments, ensuring sustained drug exposure at the target site.

Concurrently, we optimized the concentration of minocycline-loaded gelatin nanoparticles (GNP@MINO). Micro-computed tomography (micro-CT) analysis revealed that both 0.5 mg/mL and 1.0 mg/mL effectively reduced alveolar bone resorption, but 0.5 mg/mL was selected for subsequent in vivo studies to balance therapeutic efficacy and biocompatibility. The gelatin nanoparticle carrier alone exerted no protective effect (Fig. S22), confirming that the therapeutic benefit derived from minocycline loading.

To assess the anti-inflammatory effects of NMm@MINO, immunohistochemical (IHC) staining and quantitative PCR analyses were performed for interleukin-6 (IL-6) and C-reactive protein (CRP)—two key mediators of periodontal inflammation. In the untreated group, IL-6 expression increased progressively over time, peaking at day 14 (Figs. 6b and S23). In contrast, NMm@MINO treatment markedly attenuated IL-6 expression across all time points, yielding average optical density (AOD) values comparable to the control group. Notably, NMm@MINO exhibited superior suppression of IL-6 compared to either MINO or NMm alone, highlighting the synergistic anti-inflammatory effect of the combined system. Similarly, CRP expression peaked at day 3 in the untreated group and declined thereafter (Figs. 6c and S23). However, CRP levels remained consistently low in the NMm@MINO group throughout the experimental period, significantly lower than those in both the MINO and NMm groups, further underscoring the enhanced efficacy of the composite formulation.

Quantitative PCR results were fully consistent with the histological findings: NMm@MINO treatment significantly downregulated mRNA expression of *Il6* (encoding IL-6), *Crp*, and *Il1b* (encoding IL-1β) in periodontal tissues (Fig. 6d–f). Strikingly, the expression levels of these cytokines were indistinguishable from those of the control group, indicating complete suppression of the inflammatory cascade. Moreover, the inhibitory effect of NMm@MINO on these pro-inflammatory genes was significantly stronger than that of MINO or NMm alone, reinforcing the notion of a synergistic interaction between the drug and the membrane carrier. Clinical assessments demonstrated that NMm@MINO treatment reduced the gingival bleeding index (GBI) and plaque accumulation (Figs. S24 and S25), further confirming its dual capacity to resolve inflammation and control bacterial burden.

### NMm@MINO facilitates soft tissue regeneration by enhancing collagen synthesis

Functional restoration of the periodontium requires not only inflammation resolution but also the regeneration of gingival tissue, periodontal ligament (PDL) fibers, and cementum. To evaluate the regenerative potential of NMm@MINO, histological staining (H&E and Masson’s trichrome) and qPCR analysis of *Col1a1* (encoding collagen type I, Col I), a principal extracellular matrix protein, were performed.

**Fig. 7:**
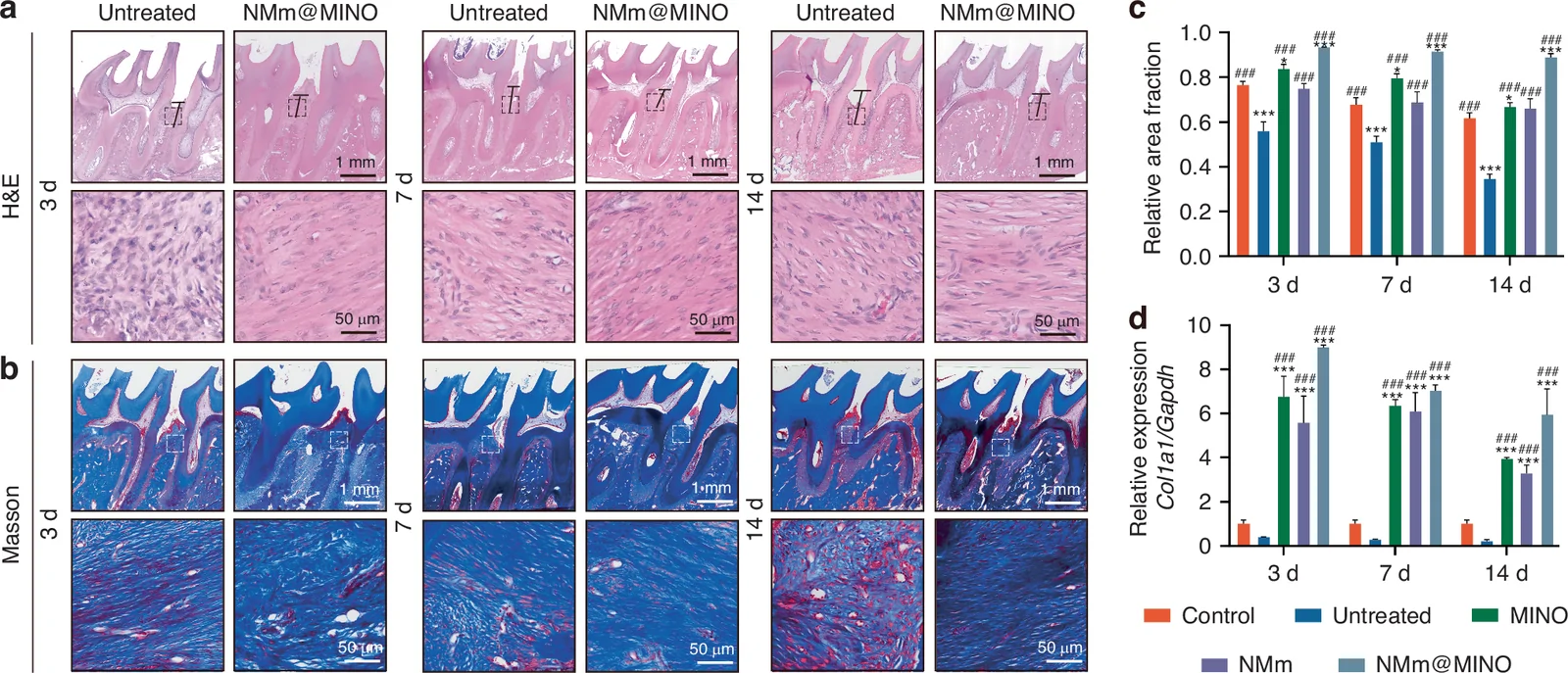
Soft tissue regeneration effect of NMm@MINO in vivo. **a** H&E staining. The black dashed boxes indicate the magnified local views of gingival fibers, periodontal ligament fibers and cementum. Scale bar = 1 mm (first row); 50 μm (second row). **b** Masson staining. The white dashed boxes represent the magnified local views of gingival fibers. Scale bar = 1 mm (first row); 50 μm (second row). **c** Quantitative analysis of collagen fiber relative area of fraction. **d** mRNA expression of *Col1a1*. Data are presented as mean ± SD, *n* = 3, compared with control group, ^*^P < 0.05, ^**^P < 0.01, ^***^P < 0.001; compared with untreated group, ^#^*P* < 0.05, ^##^*P* < 0.01, ^###^*P* < 0.001

In the untreated group, progressive deterioration of soft tissues was observed with increasing ligature duration. By day 14, severe alveolar bone loss, disrupted PDL fiber orientation, intense inflammatory infiltration, and cementum resorption were evident (Figs. 7a and S26a). Conversely, NMm@MINO treatment substantially improved tissue architecture at all time points, showing thicker bone trabeculae, aligned collagen fibers, reduced inflammatory infiltration, and new cementum formation at the root apex, features indicative of a pro-regenerative microenvironment. The restorative effects of NMm@MINO were consistently more pronounced than those in the MINO or NMm groups, suggesting a cooperative role of minocycline and the membrane vesicles in promoting tissue healing.

Masson’s trichrome staining further quantified collagen deposition (Figs. 7b, c and S26b). The NMm@MINO group exhibited significantly higher collagen-positive areas in both gingival and PDL tissues compared with those in the untreated, MINO, and NMm groups at 3, 7, and 14 days. These findings were corroborated by qPCR analysis: while MINO and NMm moderately upregulated *Col1a1* expression, NMm@MINO induced the most pronounced increase across all time points (Fig. 7d). These results demonstrate that NMm@MINO effectively and synergistically enhances collagen synthesis, thereby promoting the structural and functional regeneration of periodontal soft tissues more robustly than its individual constituents.

### NMm@MINO mitigates alveolar bone loss and restores bone metabolic balance

Given that alveolar bone loss is a major clinical manifestation of periodontitis, we next evaluated the effects of NMm@MINO on bone remodeling using micro-CT, tartrate-resistant acid phosphatase (TRAP) staining, and IHC of the RANKL/OPG signaling axis, complemented by qPCR analysis of osteogenic and osteoclastic markers.

**Fig. 8:**
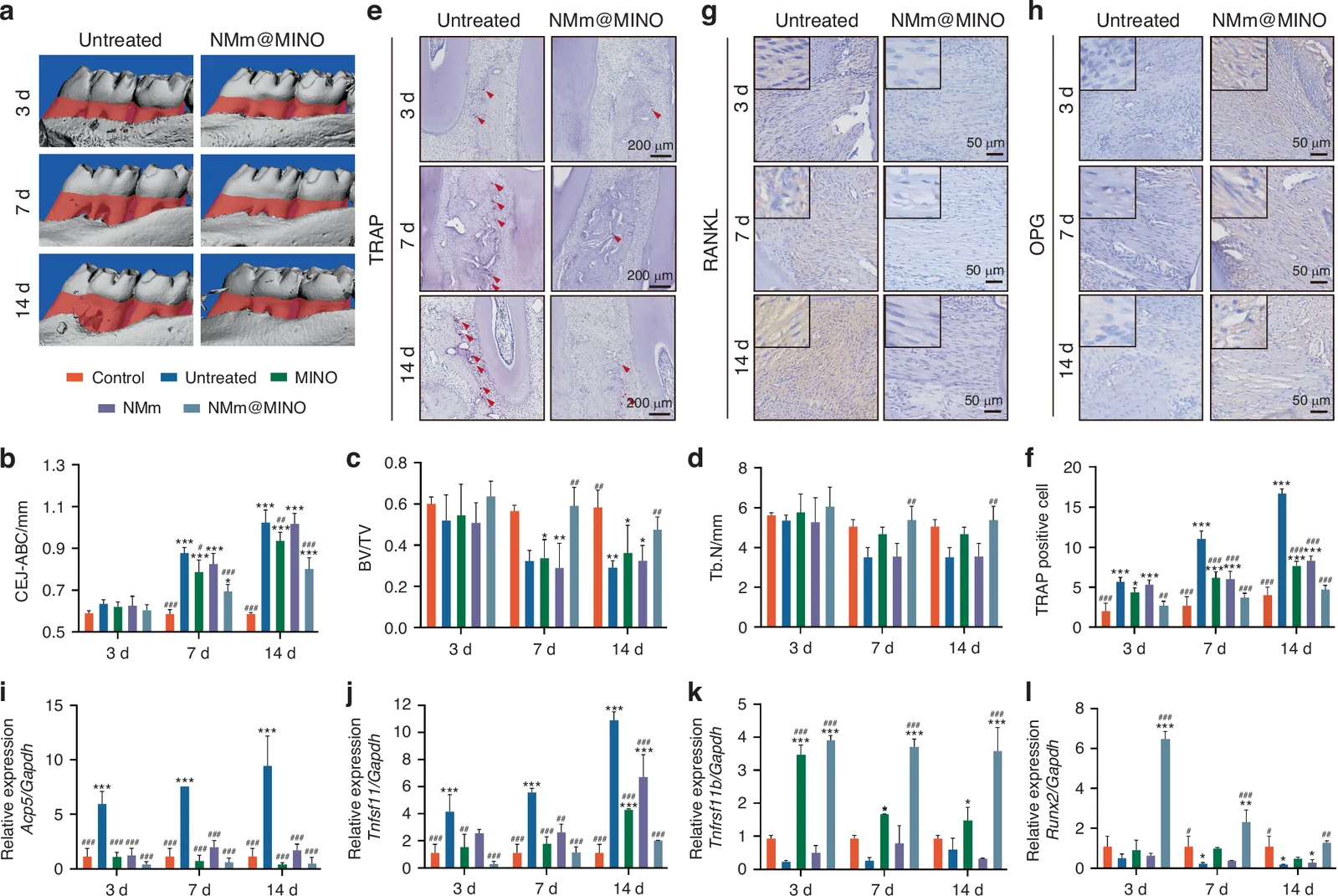
Effects of NMm@MINO on osteogenic and osteoclastic functions in vivo. **a** 3D reconstructed digitized images of the maxillary first and second molars analyzed by micro-CT. The red part displayed the exposure of root. **b** Quantitative analysis of CEJ-ABC. **c** Quantitative analysis of bone volume per tissue volume (BV/TV). **d** Quantitative analysis of Tb.N. **e** TRAP staining. Scale bar = 200 μm. **f** Quantitative analysis of the TRAP positive cell number in periodontal tissues. **g** IHC staining of RANKL in the periodontal tissue. Scale bar = 50 μm. **h** IHC staining of OPG in the periodontal tissue. Scale bar = 50 μm. **i** mRNA expression of *Acp5*. **j** mRNA expression of *Tnfsf11*. **k** mRNA expression of *Tnfrsf11b*. **l** mRNA expression of *Runx2*. Data are presented as mean ± SD, *n* = 3, compared with control group, ^*^P < 0.05, ^**^P < 0.01, ^***^P < 0.001; compared with untreated group, ^#^*P* < 0.05, ^##^*P* < 0.01, ^###^*P* < 0.001

Quantitative micro-CT analysis (Figs. 8a–d and S27a–d) revealed that the untreated group exhibited progressive alveolar bone loss, as indicated by increased CEJ-ABC distance and reduced bone volume fraction (BV/TV), trabecular number (Tb.N), thickness (Tb.Th) and bone mineral density (BMD), accompanied by elevated trabecular spacing (Tb.Sp). In contrast, NMm@MINO treatment significantly reduced CEJ-ABC distance and restored BV/TV, Tb.N, Tb.Th, Tb.Sp and BMD toward control values at days 7 and 14, demonstrating more effective preservation of bone volume and microarchitecture than either MINO or NMm treatment alone.

Histological TRAP staining revealed a time-dependent increase in multinucleated osteoclasts along the alveolar bone surface in the untreated group, which was markedly suppressed in the NMm@MINO group across all time points (Figs. 8e, f and S27e). The anti-osteoclastic effect of NMm@MINO was significantly stronger than that of MINO or NMm, underscoring the synergistic inhibition of bone resorption. Consistent with this, IHC staining showed that untreated tissues displayed elevated RANKL and reduced OPG expression (Figs. 8g, h and S27f–j). NMm@MINO treatment effectively reversed this pattern, decreasing RANKL and increasing OPG expression, with a more favorable RANKL/OPG ratio than either MINO or NMm alone, thereby reestablishing bone metabolic homeostasis more effectively.

At the transcriptional level (Fig. 8i–l), qPCR confirmed that NMm@MINO downregulated osteoclastic markers (*Acp5*, *Tnfsf11*) while upregulating osteogenic genes, including *Tnfrsf11b* (encoding OPG) and *Runx2*. The regulatory effects on these key bone metabolic markers were consistently more pronounced in the NMm@MINO group than in the MINO or NMm groups, indicating a synergistic promotion of an anabolic bone state by suppressing osteoclastogenesis and stimulating osteoblastic differentiation more effectively than the individual components.

To ensure systemic safety, hematological, histopathological, and body weight analyses were performed following repeated NMm@MINO administration. No significant abnormalities were detected in blood biochemistry, organ morphology, or body weight progression compared to the control group (Figs. S28–S30), confirming the excellent biocompatibility and systemic tolerance of NMm@MINO at therapeutic doses.

Taken together, these in vivo results comprehensively validate the therapeutic potential of NMm@MINO for periodontitis management. The formulation effectively suppresses inflammatory cytokines, promotes soft tissue regeneration through enhanced collagen synthesis, and mitigates alveolar bone resorption by rebalancing osteoclast and osteoblast activities. Critically, NMm@MINO consistently outperformed both MINO and NMm treatments across all evaluated parameters, demonstrating a clear synergistic advantage of the combined nanoplatform. Collectively, these findings highlight NMm@MINO as a multifunctional and biocompatible nanotherapeutic platform for coordinated regeneration of periodontal hard and soft tissues.

## Discussion

This study demonstrates that neutrophil-macrophage hybrid-membrane-coated, minocycline-loaded gelatin nanoparticles (NMm@MINO) provide an integrated “antibacterial-immunomodulatory-pro-regenerative” effect both in vitro and in vivo that aligns well with the multifactorial pathophysiology of periodontitis.

To achieve such integrated therapeutic outcomes, effective local drug delivery is quite critical. MINO is a well-established antibiotic for periodontitis therapy; however, systemic administration is often associated with limited local concentration, potential gastrointestinal side effects, and antibiotic resistance.^45,46^ Hence, a controlled and localized delivery strategy is essential to maximize therapeutic efficacy while minimizing systemic toxicity. The design of NMm@MINO supports such a targeted approach. The hybrid membrane gives the particles a disease-relevant biological interface,^28^^,30^ while the gelatin core responds to proteases, particularly gelatinase, which is elevated in the inflammatory periodontitis microenvironment^10^^,^^47^, enabling on-demand drug release at diseased sites.^45,46^

This protease-triggered release behavior was confirmed experimentally: the two-stage profile of NMm@MINO is consistent with the partial enzymatic cleavage of the gelatin network, which facilitates faster drug diffusion from the nanoparticle matrix. In contrast, both the enzyme-free system and the heat-inactivated gelatinase control exhibited only trace release, underscoring that the process is specifically activated by proteolytic activity rather than passive diffusion. Together, these features help extend local drug availability and reduce systemic exposure,^10,47^ which is particularly advantageous in periodontal pockets where drug retention remains challenging. In parallel with the release characterization, dual-color fluorescence co-localization on NMm@MINO indicated integrated presentation of proteins from both source membranes. This hybridized biointerface provides the structural basis for a design that aims to leverage the well-documented tropism of neutrophils for inflammatory sites and the role of macrophage membranes in enhancing circulation and targeting.^48–51^

A critical prerequisite for clinical application is the demonstration of biosafety at therapeutic doses.^52^ At the cellular level, NMm@MINO was cytocompatible with macrophages, osteogenic cells, and periodontal ligament fibroblasts, supporting its use in periodontal regeneration. Given the possibility of limited systemic exposure, because inflamed periodontal tissues are highly vascularized and locally administered nanoparticles may access the circulation^53^, blood-contact safety was assessed by in vitro hemolysis and coagulation assays, which are standard preclinical benchmarks for hemocompatibility.^54^ NMm@MINO met these criteria within the tested range, and no hematological abnormalities were detected in vivo either.

At the tissue/organ level, NMm@MINO did not induce pathological alterations in major organs (heart, liver, kidney) or impair body-weight gain, indicating a favorable safety profile at the tested dose. This profile is consistent with the protease-responsive, localized drug release of the gelatin core, which reduces systemic exposure relative to free minocycline. Considering the limitations of systemic antibiotics, including resistance and off-target toxicity, NMm@MINO’s ability to achieve therapeutic local minocycline levels while minimizing systemic effects is advantageous for long-term periodontal management.^55^

Against *P. gingivalis*, MINO and NMm@MINO reduced bacterial viability, whereas NMm alone did not differ significantly from the control. SEM imaging of MINO- and NMm@MINO-treated bacteria showed membrane corrugation and partial collapse, and intracellular ROS signals were elevated in these two groups. By contrast, the control and NMm groups retained regular morphology with low level of ROS.

These data indicate that minocycline is the primary driver of killing, and that the NMm coating by itself is not intrinsically bactericidal under our in vitro conditions. The coating therefore functions chiefly as a biomimetic carrier, consistent with prior reports that cell-membrane coatings preserve source-cell ligands and can improve targeting/retention in vivo; these aspects formed part of our design rationale rather than endpoints assessed here.^23^^,28^ Mechanistically, tetracyclines primarily inhibit protein synthesis, and the observed envelope deformations likely reflect secondary stress and/or biofilm perturbation rather than direct lysis.^56^ Collectively, the findings are consistent with a model in which NMm provides a biomimetic delivery interface, while MINO may trigger intracellular events in *P. gingivalis*, including ROS accumulation and oxidative damage, that culminate in membrane deformation and bacterial death (as supported by live/dead staining and CFU assays). Future studies using ROS scavengers or inhibitors could help establish causal links for the oxidative component.

Effective periodontitis treatment requires a dual strategy: eliminating the pathogenic biofilm and resolving the accompanying destructive host inflammatory response.^6^ Having established the robust antibacterial activity of NMm@MINO against *P. gingivalis*, we next investigated its capacity to modulate the host immune environment, a critical determinant of disease progression and tissue breakdown.^57^

NMm@MINO shifted macrophage polarization toward an anti-inflammatory state: M1-associated markers (e.g., *Nos2*, *Tnf*, *Il1b*, *Cd86*) were downregulated, whereas M2-associated markers (e.g., *Mrc1*, *Arg1*, *Tgfb1*) were upregulated, consistent with inflammation resolution. This pattern aligns with the known immunomodulatory actions of minocycline, particularly suppression of NF-κB signaling, and indicates that the released drug is a principal driver of the M2-promoting effect.^58–60^ In contrast, NMm alone elicited only a partial response: *Arg1* increased, but *Mrc1* fell below control and *Tgfb1* remained comparable to control, suggesting that the hybrid membrane components by themselves are insufficient to reproduce the breadth of the MINO-dependent phenotype. Intriguingly, the hybrid membrane NMm itself contributed significantly to the suppression of key M1 markers, suggesting that proteins or ligands presented on the fused neutrophil-macrophage membrane may actively engage with and dampen pro-inflammatory signaling pathways in the recipient macrophages.^30^^,^^61^

Notably, MINO is reported to have obvious anti-inflammatory effects and can reduce key inflammatory pathways such as TLR4/NF-κB, which helps macrophages move away from an M1-like state and toward an anti-inflammatory, tissue-repair-associated phenotype.^58,62^ Therefore, our findings indicate that the MINO released from NMm@MINO is what mainly causes a pro-M2 phenotype. NMm by itself can reduce some M1 markers, but it does not produce the full effect seen with MINO (such as the combined increase in *Mrc1* and *Tgfb1*). Beyond the drug effect, the hybrid NMm shell may also help by acting as a “decoy” surface that soaks up inflammatory signals and lowers the inputs that keep macrophages in an M1 state. A *Nature Nanotechnology* study showed neutrophil membrane-coated nanoparticles can neutralize pro-inflammatory cytokines and reduce inflammation in vivo^31^. Later mechanistic work further showed that neutrophil membrane-coated nanoparticles can have anti-inflammatory activity even without a drug, including capturing cytokines/chemokines and influencing macrophage phenotype.^30^ Overall, our results suggested that MINO accounted for most of the polarization effect, while NMm might provide an additional, supportive contribution in dampening inflammatory cues. The exact extent of this contribution will require further mechanism-focused validation.

To determine whether this macrophage reprogramming translated into broader tissue-level control of inflammation, we profiled canonical mediators in vivo. Periodontal inflammation is sustained by dysregulated *Il6*, *Crp*, and *Il1b* that amplify leukocyte infiltration and tissue injury.^63–65^ In our in vivo experiments, NMm@MINO significantly reduced these markers at both protein and mRNA levels across early, intermediate, and advanced stages, indicating robust anti-inflammatory activity. We ascribe this efficacy to the construct’s combined features, a hybrid membrane interface and a protease-responsive gelatin core. Notably, while gelatinase-triggered release was demonstrated, we did not directly quantify intra-pocket retention, biodistribution, or active targeting in vivo; thus, statements regarding enhanced bioadhesion/retention and inflammation homing should be viewed as design rationale/hypotheses. Likewise, the notion of reduced systemic exposure is inferred from localized delivery and biosafety readouts rather than pharmacokinetic measurements. The transient anti-inflammatory effect observed with free MINO is consistent with rapid local clearance, but does not by itself establish that mechanism. Collectively, these data support the premise that the integrated NMm@MINO design is important for sustained modulation of the periodontal inflammatory milieu, while targeted accumulation and pharmacokinetic advantages warrant future verification.

Alveolar bone loss in periodontitis reflects an imbalance between osteoclast-mediated resorption and osteoblast-mediated formation.^66^ In addition to its immunomodulatory effects, NMm@MINO also conferred dual benefits in bone remodeling. On the formation side, it enhanced ALP activity and upregulated *Runx2* and *Spp1*, indicating support for early and intermediate osteogenic stages, in line with reports that minocycline-based local systems can control infection/inflammation while promoting osteogenic differentiation and reducing bone loss, likely via sustained release and microenvironment remodeling.^67–69^ Interestingly, the membrane coating alone also elevated early osteogenic markers, suggesting that surface proteins on NMm (e.g., Jagged1, ephrin-B2) may stimulate pro-differentiation signaling in recipient cells even without osteoinductive drugs.^70–72^ Consistently, the superior and coordinated upregulation of all three genes by NMm@MINO points to a synergistic effect in which the nanoparticle not only delivers MINO but also leverages the inherent bioactivity of the hybrid membrane to amplify the osteogenic program, potentially through a more sustained release profile or co-activation of complementary pathways.^36^^,73,74^

On the bone resorption side, micro-CT and histological analyses confirm that NMm@MINO effectively mitigates alveolar bone resorption, as evidenced by reduced CEJ-ABC distance, improved BV/TV, and restoration of trabecular microarchitecture (Tb.N, Tb.Th, Tb.Sp). This effect is mediated through the regulation of the RANKL/OPG axis, a central pathway governing bone metabolism.^75^ NMm@MINO downregulated RANKL and upregulated OPG, thereby suppressing osteoclast differentiation and activity, as reflected by decreased TRAP-positive cells. These outcomes extend the observation that NMm@MINO inhibits osteoclastogenesis and support a dual action on bone remodeling in vivo. Finally, the protease-responsive release mechanism ensures that minocycline is preferentially liberated at inflamed sites where resorption is most active, achieving targeted regulation of bone remodeling without disrupting normal bone tissue.

At the molecular level, NMm@MINO suppressed RANKL-induced osteoclast formation and reduced the expression of *Acp5*, *Tnfrsf11a*, Ctsk, *Fos*, and *Mmp9*. Notably, *Mmp9* downregulation, a hallmark of osteoclastic matrix degradation, likely reflects diminished osteoclast activity; it may also lessen proteolytic stress on the nanoparticle matrix, thereby helping preserve carrier integrity and favoring more sustained local MINO availability at lesion sites.^76^ This interpretation is consistent with prior evidence that tetracyclines inhibit matrix metalloproteinases^42^.

Taken together, these readouts indicate attenuation of downstream effector programs of osteoclasts (including MMP-9-mediated matrix turnover). To account for upstream initiation, a plausible model is that proteins displayed on the hybrid membrane act as decoy receptors that partially sequester soluble RANKL and dampen RANKL-RANK engagement, while minocycline concurrently suppresses intracellular pro-resorptive signaling, a complementary “decoy + intracellular” mechanism.^35,36^ Although this framework is compatible with the observed phenotype, it remains hypothetical; definitive validation will require targeted experiments (e.g., receptor blocking or ligand neutralization to test the decoy component, and pathway inhibition to map intracellular nodes). We did not delineate the specific membrane protein-ligand interactions underlying osteoclast modulation in this study; future work will profile candidate membrane proteins and establish their functional relevance.

Durable periodontal recovery requires not only infection and inflammation control but also active repair of gingiva, periodontal ligament (PDL), and cementum, processes that are often neglected by conventional treatments. Experimentally, NMm@MINO enhanced rPDLF proliferation, migration, and type I collagen production, core processes for soft-tissue repair. Its superior effect over individual components suggests that the hybrid membrane enhances cellular interaction or uptake, facilitating a more pro-regenerative fibroblast response.^74,77,78^ These benefits are likely attributable to the combined effects of the biomimetic interface, moderated inflammatory tone, and sustained local drug release.^77,78^ Consistent with these cellular findings, in vivo analyses showed increased Col I expression and restoration of parallel collagen-fiber alignment in periodontal tissues, indicating reconstitution of PDL architecture—a functionally meaningful outcome given the role of organized fibers in force transmission and alveolar bone stability.^79–81^ Together, these results highlight NMm@MINO’s capacity to address the soft-tissue dimension of periodontal regeneration that conventional mechanical or antibiotic therapies typically overlook.

Among existing periodontitis treatment strategies, the most clinically established approach is the combination of scaling and root planing (SRP) with local drug delivery (LDD), such as doxycycline gel, minocycline ointment and chlorhexidine mouthwash.^82^ The main advantages of these products are their mature administration routes and the ability to achieve higher local drug concentrations while reducing systemic exposure. Additionally, abundant clinical evidence supports their use alongside SRP. While it is true that these treatments focus on the proactive reduction of local pathogens, they are often limited in their ability to concurrently address immune dysregulation and the requirements for hard and soft tissue regeneration. Moreover, in deep and complex periodontal pockets, therapeutic outcomes may remain variable due to limited retention and the protective barrier of biofilms. In contrast, emerging advanced biomaterials in recent years (e.g., ROS/hypoxia-responsive nanogels, injectable multifunctional hydrogels, biomimetic hydrogels, or MOF-/photothermal-chemical synergistic systems) increasingly emphasize multi-target synergy spanning antibacterial-immunomodulation-regeneration.^12,83–86^ Many of these platforms need an external trigger, such as light or NIR irradiation. This can be limited by penetration depth and by differences in irradiation settings across studies and clinics.^87,88^ In addition, some systems mainly focus on either a single function or a limited set of functions (e.g., antioxidation, photothermal bactericidal action, or osteogenesis promotion), so the overall effect (on-demand release, immune evasion/targeted enrichment, and coordinated hard/soft tissue reconstruction) may be incomplete in complex periodontal lesions.

In this context, the distinctive advantages of the NMm@MINO system lie in its endogenous stimulus-responsiveness and biomimetic interface. Specifically, it employs elevated proteases (MMP/gelatinase) in the periodontitis microenvironment as an “internal trigger” to enable enzyme-responsive release from a gelatin core, thereby improving on-demand drug delivery efficiency within periodontal pockets (~50% release at ~2 h and approaching equilibrium at ~8 h). Meanwhile, the hybrid neutrophil-macrophage cell membrane provides a disease-relevant biointerface that may enhance local retention and inflammation-associated interactions. Importantly, NMm@MINO is not merely designed for “bacterial killing,” but rather integrates the antibacterial and immunomodulatory effects of minocycline with the immunologically active, biomimetic membrane interface. It achieves > 99% bactericidal efficiency against *P. gingivalis* and *S. aureus*, promotes macrophage polarization toward an M2-like phenotype, and downregulates inflammatory cytokines. Consequently, in vivo it enhances soft-tissue collagen deposition, restores the balance of the bone remodeling axis (RANKL/OPG), and improves alveolar bone microstructure, collectively demonstrating a system-level “antibacterial-immune remodeling-soft and hard tissue regeneration” advantage.

**Fig. 9:**
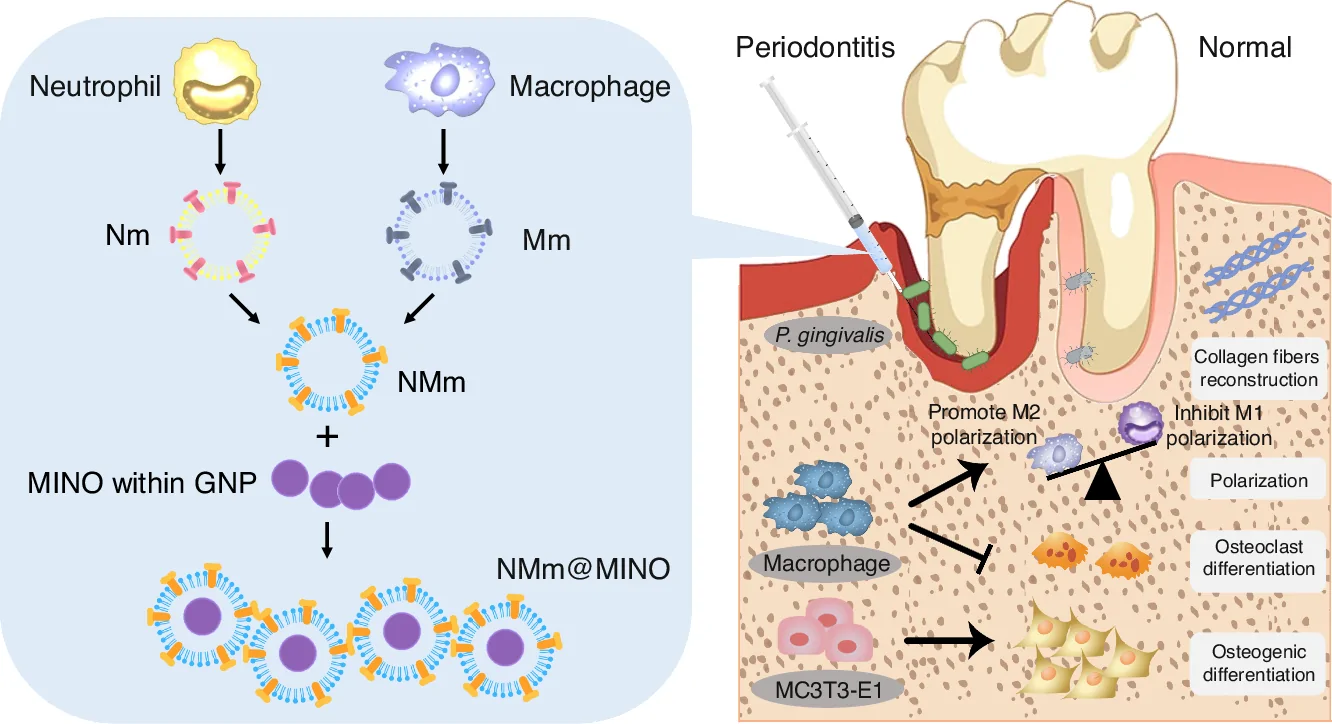
Schematic diagram: bioinspired design of the hybrid membrane-coated NMm@MINO nanoparticle for coordinated antibacterial, immunomodulatory, and pro-regenerative functions in periodontitis

Collectively, our evidence demonstrates that NMm@MINO orchestrates a pro-regenerative microenvironment through integrated antibacterial effects and coordinated regulation of cellular functions, enhancing osteogenic potential, suppressing osteoclastogenesis, and promoting soft-tissue repair (Fig. 9). This combined anti-infective, immunomodulatory, and pro-regenerative action underscores its potential as a comprehensive therapeutic strategy for the multifactorial pathology of periodontitis. Of course, our study also has some limitations. First, NMm@MINO contains an antibiotic, so we still need to check resistance risk and possible changes to the oral microbiome after repeated use. Second, cell membrane-based materials are harder to make at scale. They also need strict quality control to keep batches consistent. We also need to ensure the membrane remains active and stable during processing and storage. Third, we did not directly measure how long NMm@MINO stays in periodontal pockets or how much “active targeting” it truly adds. Future work will use imaging/tracking and biodistribution studies, and head-to-head comparisons with standard clinical LDD products.

## Materials and methods

### Cell isolation and culture

NR8383, RAW 264.7, MC3T3-E1 cell lines and rPDLFs were procured from Procell Life Science & Technology Co., Ltd (Wuhan, China). NR8383 cells were maintained in Ham’s F-12K medium supplemented with 20% fetal bovine serum (FBS) (Vazyme, F103-03, China) and 1% penicillin/streptomycin (P/S). RAW 264.7 was cultured in Dulbecco’s Modified Eagle Medium (DMEM) along with 10% FBS and 1% P/S. MC3T3-E1 and rPDLFs were cultured with alpha-Modified Eagle’s Medium (α-MEM) containing 10% FBS and 1% P/S. Primary neutrophils were isolated from 3-week-old Sprague Dawley (SD) rats via density gradient centrifugation. Following euthanasia, femora and tibiae were aseptically separated. Bone marrow was flushed using sterile culture medium to obtain single-cell suspensions. Neutrophil isolation was performed according to the manufacturer’s protocol for the Rat Bone Marrow Neutrophil Isolation Kit (Solarbio, P2610, China).

Immunofluorescence staining was performed to identify NR8383 cells and rat primary neutrophils using RANK and myeloperoxidase (MPO) markers, respectively. Cells were seeded onto glass coverslips, fixed with 4% paraformaldehyde (30 min, RT), and permeabilized with 0.25% Triton X-100. Endogenous peroxidase activity was quenched with 3% H_2_O_2_ (15 min), followed by blocking with 5% bovine serum albumin (BSA) for 1 h. Primary antibodies were applied overnight at 4 °C: rabbit anti-RANK (1:100, Huabio, ER1915-70, China) for NR8383 cells and rabbit anti-MPO (1:100, Huabio, EM1901-19, China) for neutrophils. After PBS washes, samples were incubated with goat anti-rabbit IgG secondary antibody and DAPI nuclear counterstain (1 μg/mL, 3 min) at 37 °C. Fluorescence images were acquired using a Leica DMi8 inverted microscope.

### Membranes extraction

The plasma membrane fraction was isolated from NR8383 cells and neutrophils following an established protocol with minor modifications.^31,89^ Briefly, NR8383 cells were collected from the culture medium, counted, and pelleted by centrifugation at 1 000 × *g* for 5 min. The macrophage pellet was resuspended in 2 mL of cold Tris-magnesium buffer, mixed with 2 mL of 0.5 mol/L sucrose solution to achieve a final sucrose concentration of 0.25 mol/L. Cell lysis was performed by continuous homogenization in tissue grinding tubes for 5 min. The lysate was centrifuged at 2 000 × *g* (4 °C) for 15 min to remove cellular organelles. The supernatant was then subjected to further centrifugation at 10 000 × *g* (4 °C) for 30 min to isolate the crude membrane fraction. The resulting pellet was resuspended in ice-cold 0.25 mol/L sucrose solution using vigorous vortexing followed by brief ultrasonication. Membranes were re-pelleted by centrifugation at 10 000 × *g* (4 °C) for 30 min. The purified membrane fraction was finally resuspended in PBS using an ultrasonic water bath combined with repetitive pipetting. The PBS volume was standardized at 1 mL per 5 × 10^5^ initial cells and aliquoted into 1.5 mL microcentrifuge tubes. For neutrophils, the cell pellet was resuspended in approximately 4 mL of pre-chilled hypotonic lysis buffer. Cell lysis was achieved by continuous homogenization in tissue grinding tubes for 5 min. The lysate was transferred to 1.5 mL microcentrifuge tubes and centrifuged at 2 000 × *g* (4 °C) for 15 min to remove cellular debris. The supernatant was collected, resuspended, and further centrifuged at 10 000 × *g* (4 °C) for 30 min. After discarding the supernatant, the opaque pellet was retained. Membrane purification was performed by washing and resuspending the pellet in 0.2 mmol/L EDTA solution, followed by centrifugation at 17 000 × *g* (4 °C) for 30 min to concentrate membranes at the tube base. Purified membranes were resuspended in PBS (volume standardized at 1 mL per 5 × 10^5^ initial cells per 1.5 mL microcentrifuge tube). All membrane preparations were stored at −80 °C until use.

Membranes from macrophages and neutrophils were isolated and quantified using a microBCA protein assay kit (Sangon Biotech, Shanghai, China). Membrane proteins from both cell types were then combined at a 1:1 protein mass ratio. The mixture underwent sonication at 80 W and was extruded sequentially ten times through polycarbonate membranes with decreasing pore sizes of 800 nm, 400 nm, and 200 nm at 37 °C using a mini-extruder (Avanti Polar Lipids, USA) to produce hybrid membrane vesicles.

### Preparation of nanoparticles

Gelatin nanoparticle (GNP) cores were synthesized following a standard two-step desolvation method as previously described.^90^ Briefly, type-A gelatin was subjected to initial desolvation by acetone, followed by pH adjustment and a second acetone-induced desolvation, with subsequent crosslinking using glutaraldehyde. Nanoparticles were purified by washing with deionized water.

To load minocycline, lyophilized GNPs were dispersed in an aqueous minocycline solution (20 mg/mL) and stirred gently for 24 h. Unbound minocycline in the supernatant was quantified using high-performance liquid chromatography (HPLC) to determine the loading capacity and encapsulation efficiency. Encapsulation efficiency (EE, %) and loading capacity (LC, µg MINO per mg GNP) were calculated as EE = (M_total - M_free)/M_total × 100%, LC = (M_total - M_free)/M_GNP.

The hybrid membrane vesicles were coated onto the minocycline-loaded GNP cores at a polymer-to-membrane protein mass ratio of 2:1 via sonication (probe sonicator, 80 W, 2 min) to produce membrane-coated nanoparticles (NMm@MINO).

Particle size and ζ-potential were measured at 25 °C by dynamic light scattering using a Zetasizer 3000 (Malvern, UK), with results reported as averages of triplicate measurements. Nanoparticle morphology was characterized using transmission electron microscopy (JEM-2100 Plus, JEOL; 200 kV) following negative staining with 2% phosphotungstic acid.

### Drug release

The minocycline release profiles were investigated using Slide-A-Lyzer MINI dialysis devices (Thermo Fisher Scientific; MWCO = 3.5 kDa). PBS, GNP@MINO (minocycline-loaded GNPs), NMm@MINO (membrane-coated GNP@MINO), and NMm@MINO treated with heat-denatured gelatinase were placed in dialysis units immersed in 50 mmol/L HEPES buffer containing 2 mmol/L CaCl_2_ (pH 7.4) and gelatinase concentrations ranging from 0 to 200 μg/mL. At specified intervals, external buffer aliquots were sampled, replenished with fresh buffer, and analyzed by HPLC to quantify the cumulative minocycline release.

### Hybrid cell membranes characterization

NR8383 macrophages and rat primary neutrophils were fluorescently labeled using PKH26 (red fluorescence) (Solarbio, D0030, China) and PKH67 (green fluorescence) (Solarbio, D0031, China) lipophilic dyes, respectively, according to the manufacturer’s protocols. Membrane fractions and hybrid cell membranes were prepared using the previously described methodology. Then, the hybrid cell membranes were resuspended in PBS. Cell-covered coverslips were incubated with the fused membrane suspension. Colocalization of red (Ex/Em 551/567 nm) and green (Ex/Em 490/502 nm) fluorescence signals was analyzed using a Leica DMi8 inverted microscope equipped with TRITC and FITC filter sets.

### SDS-polyacrylamide gel electrophoresis (SDS-PAGE)

Samples containing 10 μg of total protein were mixed with 5× SDS loading buffer (250 mmol/L Tris-HCl pH 6.8, 10% SDS, 0.5% bromophenol blue, 50% glycerol, 5% β-mercaptoethanol) and heated at 95 °C for 10 min. The denatured proteins were subjected to electrophoresis on 12% SDS-polyacrylamide gels. Electrophoresis was performed at 80 V for 30 min through the stacking gel, followed by 120 V for approximately 90 min until the bromophenol blue front reached the bottom of the gel. After electrophoresis, the gel was stained with 0.25% (w/v) Coomassie Brilliant Blue R-250 for 1 h at room temperature with gentle shaking. The gel was then destained in a solution containing 40% methanol and 10% acetic acid overnight. Gel images were captured using a Gel Doc XR+ imaging system.

### CCK-8

RAW264.7 cells were seeded into a 96-well plate at a density of 1 × 10^3^ cells per well and allowed to adhere for 12 h in a humidified incubator (37 °C, 5% CO_2_). Following adhesion, cells were treated with varying concentrations of NMm nanoparticles and incubated for 6, 12, or 24 h. rPDLFs were plated in 96-well plates at a density of 5 × 10^3^ cells per well and allowed to adhere for 12 h. Subsequently, the cells were treated with various drugs or nanoparticles for 1, 3, and 5 days to assess cell proliferation. At each designated time point, 90 μL of fresh DMEM medium and 10 μL of CCK-8 solution (APE ×BIO, USA) were added to each well. The plate was then incubated in the dark for 1 h. Absorbance was measured at 450 nm using a microplate reader. Relative cell viability (%) was calculated as follows:

$${{\rm{Cell\; viability}}}( \% )=100\times ({{{\rm{OD}}}}_{{{\rm{Treatment}}}}-{{{\rm{OD}}}}_{{{\rm{Blank}}}})/({{{\rm{OD}}}}_{{{\rm{Control}}}}-{{{\rm{OD}}}}_{{{\rm{Blank}}}})$$

All experiments were performed with seven replicates (*n* = 7) per group.

### Live/dead staining

RAW264.7 and MC3T3-E1 cells were plated in 12-well plates at a density of 1 × 10^5^ cells per well. Following a 12-h adhesion period under standard culture conditions (37 °C, 5% CO_2_), cells were exposed to various drug or nanoparticle treatments for 24 h. After treatment, cell viability was assessed using a Calcein-AM/PI Double Stain Kit (Solarbio, China) according to the manufacturer’s instructions. Briefly, cells were incubated with staining solution containing 2 μmol/L Calcein-AM and 5 μmol/L PI. All staining procedures were performed in the dark. Stained samples were visualized using a fluorescence microscope (Leica DMi8, Germany). Live cells (Calcein-AM positive, green fluorescence) and dead cells (PI positive, red fluorescence) were distinguished using appropriate filter sets. For quantitative assessment, dead cell counts were performed in three randomly selected microscopic fields per sample.

### Cytoskeleton staining

Employing identical cell inoculation and processing protocols as those used for live/dead staining, cells were co-cultured with experimental treatments for 24 h, fixed with 4% paraformaldehyde (20 min at room temperature), permeabilized with 0.25% Triton X-100 (8 min), and sequentially stained with TRITC-phalloidin (1:200 dilution) and DAPI (1 μg/mL). Fluorescence imaging was conducted using an inverted microscope (Leica DMi8, Germany) with TRITC (557/576 nm) and DAPI (358/461 nm) filter sets. For semi-quantitative morphometric analysis, five individual cells per experimental group were randomly selected, and cellular aspect ratios (length/width) were determined through ImageJ software.

### Blood compatibility assessment

Standardized hemolysis analysis was performed using freshly collected sterile rabbit whole blood. Test samples contained 200 μL diluted blood mixed with 5 mL of varying filtered test solution. Negative controls consisted of 200 μL diluted blood in 5 mL PBS, while positive controls used 5 mL deionized water instead of PBS. All mixtures were incubated at 37 °C for 30 min, centrifuged at 3 000 r/min (4 °C, 5 min), and supernatant optical density was measured at 545 nm using a microplate reader. Hemolysis ratio (HR) was calculated as:

$${{\rm{HR}}}( \% )=100\times ({{{\rm{OD}}}}_{{{\rm{Treatment}}}}-{{{\rm{OD}}}}_{{{\rm{Negative}}}})/({{{\rm{OD}}}}_{{{\rm{Positive}}}}-{{{\rm{OD}}}}_{{{\rm{Negative}}}})$$

For the blood coagulation assay, test samples were combined with 200 μL ACD-anticoagulated rabbit blood and 20 μL 0.2 mol/L CaCl₂ solution. After vortex mixing, samples were incubated at 37 °C for 10 min. Subsequently, 50 mL sterile water (pre-warmed to 37 °C) was added to each mixture. Absorbance of the resulting solutions was measured at 545 nm using a UV-vis spectrophotometer. Control samples contained 200 μL ACD blood mixed with 50 mL pre-warmed sterile water. The blood coagulation index (BCI) was calculated as:

$${{\rm{BCI}}}( \% )={{{\rm{OD}}}}_{{{\rm{Treatment}}}}/{{{\rm{OD}}}}_{{{\rm{Control}}}}\times 100$$

### Spread plate test

*Porphyromonas gingivalis* (ATCC 33277) suspensions (10^6^ CFU/mL) were dispensed into 48-well plates (300 μL/well). Cultures were treated with drug-supplemented media and incubated anaerobically at 37 °C for 72 h in an anaerobic incubator. The tested concentrations were 80 μg/mL for NMm and 10.4 μg/mL for MINO. Accordingly, the NMm@MINO formulation was prepared to maintain these same concentrations, containing 80 μg of NMm and 10.4 μg of MINO per milliliter. Following incubation, bacterial suspensions were serially diluted 10^4^-fold in sterile PBS, and 100 μL aliquots were plated on blood agar. After 4 days of anaerobic cultivation at 37 °C, colonies were enumerated using image-based quantification (ImageJ software).

For *Staphylococcus aureus* (ATCC 25923), suspensions were adjusted to 10^6^ CFU/mL and dispensed into 48-well plates (300 μL per well). Media supplemented with the same concentrations of NMm, MINO, or the NMm@MINO formulation were added. The plates were then incubated at 37 °C under aerobic conditions for 24 h. Subsequent steps were identical to those described above: cultures were diluted 10^5^ fold, and 100 μL aliquots were plated on LB agar plates. After 24 h of incubation at 37 °C, colonies were counted using ImageJ software. All conditions were tested in triplicate biological replicates.

### OD_600_ measurement

Following the spread plate method, bacterial suspensions were collected at 0, 24, 48, and 72 h after drug exposure. After appropriate dilution, the optical density at 600 nm (OD_600_) of each sample was measured using a microplate reader.

### Bacterial morphology observation

Employing identical bacterial inoculation and processing protocols as those used for the spread plate method, samples were collected 72 h after seeding. Following incubation, bacterial suspensions were smeared onto substrates. Then the samples were chemically fixed (2.5% Glutaraldehyde EM Grade, 4 °C, overnight), dehydrated through an ethanol gradient, sputter-coated with gold. The bacteria were observed using a scanning electron microscope (JEOL Ltd, Japan) at a voltage of 20 kV.

### Bacterial live/dead and ROS staining

Bacterial samples were prepared identically for the SEM protocol. Following smear preparation, specimens were stained with a live/dead viability kit (Solarbio, China) and a ROS staining kit (Solarbio, China), respectively, according to the manufacturer's instructions. Fluorescence imaging was performed using a Leica DMi8 microscope (Germany). Respective fluorescence intensities were quantified via ImageJ, with all experimental conditions assessed in triplicate biological replicates.

### Real-time quantitative PCR

#### Cell culture and differentiation assays

RAW 264.7 macrophages were plated in 6-well plates (4 × 10^5^ cells per well) for parallel experiments: (i) For inflammatory response analysis, control groups remained untreated while experimental groups received 100 ng/mL LPS (2 h) followed by test compound/nanoparticle administration, with RNA extraction at 24 h post-treatment; (ii) For osteoclastogenesis, cultures were switched to osteoclast induction medium (culture medium + 10% FBS + 50 ng/mL RANKL (R&D Systems, USA) with medium replenishment every 48 h, followed by harvest on day 4. Concurrently, MC3T3-E1 pre-osteoblasts (4 × 10^5^ cells per well) were differentiated in osteogenic induction medium containing test agents for 7 days prior to collection.

#### Tissue sample collection

Following euthanasia, gingival tissues surrounding the molars of rats were harvested and pooled in sterile RNase-free tubes, then immediately stored in liquid nitrogen for preservation.Grinding of gingival tissue samples: (i) Prior to use, the tissue grinder (Shanghai Jingxin Industrial Development Co., Ltd) was precooled to −50 °C to maintain sample integrity. (ii) Each tissue sample was transferred to a nuclease-free EP tube, mixed with 1 mL of RNAiso Plus reagent, and combined with stainless steel grinding beads. (iii) The grinder was operated at 60% power output with intermittent cycles (10 s ON, 5 s OFF) for 30 repetitions to fully grind the gingival tissue sample.

#### Gene expression analysis

Total RNA was isolated using FastPure Cell/Tissue RNA Kit (Vazyme, RC112-01, China), followed by cDNA synthesis with HiScript III RT SuperMix (+gDNA wiper; Vazyme, R323-01, China). Quantitative PCR employed Taq Pro Universal SYBR Master Mix (Vazyme, Q712-02, China) on an Applied Biosystems 7300 platform. Relative transcript levels were normalized to GAPDH and calculated via the 2^−ΔΔCt^ method, with primer sequences detailed in Table S2 (Supporting Information).

### Immunofluorescence staining and semi-quantitative analysis

RAW 264.7 macrophages were plated in 12-well plates (1 × 10^5^ cells per well) for two parallel assays: (i) Inflammatory response: Control groups received no treatment, while experimental groups were stimulated with 100 ng/mL LPS (2 h) followed by test compound/nanoparticle administration. RNA was extracted 24 h post-treatment. (ii) Osteoclast differentiation: Cells were induced with osteoclastogenic medium (culture medium + 10% FBS + 50 ng/mL RANKL; R&D Systems USA) for 4 days with medium replenishment every 48 h. rPDLFs were seeded in 12-well plates at a density of 1 × 10^5^ cells per well and allowed to adhere for 12 h. The cells were then treated with various experimental conditions. After 3 days of treatment, the samples were harvested and fixed for subsequent analysis.

Fixed cells were treated for Immunofluorescence Staining according to established protocols using: anti-iNOS (1:100; Huabio, ER1706-89, China), anti-CD206 (1:200; Huabio, ET1702-04, China), anti-c-Fos (1:1 000; Huabio, HA722839, China) and anti-COL1 (1:500; Huabio, HA722517, China) followed by species-matched fluorescent secondary antibodies. Following staining, three random fields per sample were captured using a fluorescence microscope (Leica DMi8, Germany). Fluorescence intensity was quantified in ImageJ software.

### Inflammatory cytokine neutralization assay

To evaluate the decoy-like inflammatory cytokine sequestration ability of different membrane-coated nanoparticles, IL-1β and IL-6 were selected as representative proinflammatory cytokines associated with periodontitis. Recombinant IL-1β (Elabscience, PDEM100209, China) and IL-6 (Elabscience, PKSM041097, China) standard solutions were prepared at final concentrations of 300 pg/mL and 400 pg/mL, respectively. The cytokine solutions were then incubated with GNP, Nm@GNP, Mm@GNP, or NMm@GNP.

For consistency with the subsequent cell experiments, the concentration of GNP was set at 160 μg/mL, and the membrane protein concentration in membrane-coated nanoparticle groups was set at 80 μg/mL. Cytokine solutions without nanoparticle treatment served as the control group. After incubation at 37 °C for 6 h, the mixtures were centrifuged at 15 000 × *g* for 20 min to remove the nanoparticles and nanoparticle-bound cytokines. The supernatants were carefully collected, and the residual concentrations of free IL-1β and IL-6 were determined using commercial ELISA kits for IL-1β and IL-6 (Elabscience, E-EL-M0037 and E-EL-M0044, respectively, China) according to the manufacturer’s instructions.

The absorbance was measured at 450 nm using a microplate reader, and cytokine concentrations were calculated according to the corresponding standard curves. The cytokine neutralization capacity of each formulation was evaluated by comparing the residual cytokine concentrations in the supernatants. Lower residual cytokine levels indicated stronger cytokine sequestration ability. All experiments were performed in triplicate.

### Alkaline phosphatase (ALP) staining

MC3T3-E1 cells were seeded in 6-well plates at 4 × 10^5^ cells per well. After 24 h, cultures were switched to osteogenic induction medium supplemented with experimental compounds/nanoparticles, with medium replenishment every 48 h. Following 7 days of osteogenic induction, cells were washed with PBS, fixed in 4% paraformaldehyde (RT, 30 min), and subjected to alkaline phosphatase (ALP) cytochemical staining using a commercial kit (Beyotime Biotechnology, China) per manufacturer’s protocol. Stained specimens were incubated for 2 h at 37 °C. The images were acquired using a camera and a microscope (Leica, DMi1, Germany).

### Tartrate resistant acid phosphatase (TRAP) staining

RAW 264.7 macrophages were seeded in 96-well plates at a density of 3 × 10^3^ cells per well and allowed to adhere overnight. Following adhesion, the culture medium was replaced with 200 µL of osteoclast induction medium consisting of culture medium supplemented with 10% fetal FBS and 50 ng/mL soluble RANKL (R&D Systems, USA). The induction medium was refreshed every 48 h. On Day 4 post-induction, the medium was aspirated, the cells were gently washed twice with PBS, and fixed. Tartrate-resistant acid phosphatase (TRAP) staining was then performed according to the manufacturer’s instructions using a commercial TRAP staining kit (Solarbio, CA5030, China). Images of the TRAP-stained cells were acquired using a digital camera mounted on an inverted microscope (Leica DMi1, Germany).

### Transwell assay

After 24 h of serum starvation, rPDLFs were seeded into the upper chamber of a Transwell insert at a density of 2 × 10^4^ cells per well in 200 μL of serum-free α-MEM. The lower chamber was filled with complete medium containing the respective drug treatments. Following 24 h of incubation at 37 °C in a 5% CO_2_ atmosphere, the cells were fixed and stained with crystal violet. Migrated cells were visualized and imaged under a microscope and quantitatively analyzed using ImageJ software.

### Experimental rat periodontitis model construction

Sprague-Dawley rats (8 weeks old) were used to establish the ligature-induced periodontitis model. All animal experiments were approved by the Animal Ethics Committee at Chongqing Medical University (AECCMU-2020-004). The animals were randomly divided into five experimental groups (*n* = 6 per group per time point): control group (healthy, no ligature), untreated group (periodontitis + no treatment), MINO group (periodontitis + free minocycline), NMm group (periodontitis + hybrid membrane vesicles), NMm@MINO group (periodontitis + neutrophil-macrophage hybrid-membrane-coated, minocycline-loaded gelatin nanoparticles). Periodontitis was induced by placing a stainless-steel ligature (diameter = 0.2 mm) with secured silk sutures (3–0) around the cervical area between the maxillary first and second molars, and was then fixed with composite resin. Ligatures were checked daily and replaced if lost. The ligatures were maintained for 3, 7, and 14 days to simulate early, intermediate, and advanced disease stages, respectively. Following ligature removal at respective time points, therapeutic agents were administered via local injection into the periodontal ligament space using a micro-syringe (Hamilton, USA). Based on preliminary retention imaging at 0, 24, 48, and 72 h post-injection, the optimal dosing interval was determined to be every 3 days. The MINO group received 20 μL of free minocycline solution at 0.5 mg/mL per injection site; the NMm group received 20 μL of hybrid membrane vesicle suspension per injection site; and the NMm@MINO group received 20 μL of NMm@MINO suspension at 0.5 mg/mL per injection site. Rats were euthanized on days 10, 28, and 35, respectively, corresponding to different therapeutic durations after ligature removal. Maxillae were collected for subsequent histological and micro-CT analyses.

### Periodontal clinical assessments

#### GBI

The GBI was assessed at 1, 3, 7, and 21 days after model establishment to evaluate the dynamic changes in gingival inflammation. During each assessment, a standardized periodontal probe (Williams probe) was gently inserted into the gingival sulcus at six sites per tooth (mesiobuccal, midbuccal, distobuccal, mesiolingual, midlingual, distolingual) around the maxillary first and second molars. The presence or absence of bleeding within 30 s was recorded. The GBI was calculated based on the number of bleeding sites.

#### Plaque staining

Plaque staining was performed on day 3 after model establishment using a commercial disclosing solution (Erythrosin, Sigma-Aldrich) for visual observation. At the designated time point, anesthetized rats received a topical application of the 0.5% Erythrosin solution, evenly coated on the crown surfaces of the maxillary first and second molars using a small brush. The plaque staining patterns on the tooth surfaces were then directly observed and photographed.

### Histological analysis

#### H&E staining

Tissue sections were processed using the H&E staining kit (Solarbio, China) in accordance with the manufacturer’s protocol. Microscopic visualization was performed using a high-resolution slide scanner (VS-200, Olympus, Japan) to capture images of the target regions.

#### TRAP staining

The TRAP staining procedure was carried out with a commercial TRAP staining kit (Solarbio, China). Digital images of stained samples were acquired using a high-resolution slide scanner (VS-200, Olympus, Japan). Osteoclasts, identified as TRAP-positive multinucleated cells (defined as clusters containing three or more nuclei), were quantified in the interdental bone region between the first and second maxillary molars. To mitigate bias, the investigator remained unaware of group assignments during data collection and analysis.

#### Masson’s trichrome staining

Paraffin-embedded sections of rat maxillae were stained with a Masson’s trichrome staining kit (Solarbio Science & Technology Co., Ltd, Beijing, China), followed by histopathological examination under a light microscope (VS-200, Olympus, Japan). The collagen fiber area fraction in the gingival and PDL tissues was quantified using ImageJ software (National Institutes of Health, USA).

### Micro-computed tomography analysis

The maxilla was scanned using a Scanco μCT40 scanner (Viva CT 40, Scanco Medical) at a resolution of 15 μm (70 kV, 113 μA). The raw scan data were reconstructed to obtain a three-dimensional reconstruction of the samples. Periodontitis-induced alveolar bone loss was quantified based on measurements of linear bone loss, bone volume (BV), tissue volume (TV), and bone mineral density (BMD). The linear distance from the cementoenamel junction to the top of the alveolar crest (CEJ-ABC) between the first and second molars was measured to assess the degree of alveolar bone destruction. In addition, the ratio of bone volume to tissue volume (BV/TV) and BMD of each sample were measured. The region of interest (ROI) was determined as an 18 × 18 pixel^2^/μm² area on the alveolar bone between the first and the second molar with a height of 50 slices.

### Immunohistochemical (IHC) staining

Before staining, slides were baked at 65 °C for 2 h. After deparaffinization and rehydration, slides were subjected to antigen retrieval with 0.2% proteinase (ZLI-9010, ZSGB-BIO, China) at 37 °C for 15 min. Then the slides were stained according to the manufacturer’s instructions (SP-0022, Bioss, China). After washing with PBS, slides were incubated with 0.3% hydrogen peroxide at 37 °C for 20 min. After PBS washing, slides were incubated with goat serum at 37 °C for 20 min to block nonspecific binding. Drain the slides and carefully wipe off the excess serum blocking reagent (do not rinse with PBS). Slides were then incubated with primary antibody (Bioss, China, 1:100) at 4 °C overnight. After PBS washing, the slides were incubated with goat anti-rabbit secondary antibody for 20 min at 37 °C. After PBS washing, incubate samples with 1–3 drops of HSS-HRP for 20 min at 37 °C. After PBS washing, the slides were then stained with 3,3-diaminobiphenyl tetrasodium (DAB) substrate for 20 s, and counterstained with hematoxylin. The staining images were scanned and obtained with Olympus scanner (VS-200, Japan). Positive cells were counted in two random 100 × 100 μm^2^ square areas of the gingiva and periodontal ligament between the first and second molars. The investigator was blinded to the groups during data measurement and outcome evaluation.

### Statistical analysis

All data were presented as means ± standard deviation (SD). To ensure reproducibility, independent biological experiments were carried out three or more times. SPSS 22.0 software was used for statistical analysis. Differences were analyzed by Student’s *t*-test and one-way ANOVA with Dunnett’s post hoc test, with statistical significance defined at *P* < 0.05.

## Supplementary information

Supporting Information
