# Supplementary material for: A biomimetic nanoparticle coordinates antibacterial and immunomodulatory functions to promote periodontal regeneration
Source: Int J Oral Sci. 2026 Oct 26;18:67. doi: 10.1038/s41368-026-00459-y (PMC13710849; doi:10.1038/s41368-026-00459-y)
Supplement: Supplementary file 1 [file 41368_2026_459_MOESM1_ESM.pdf]

A biomimetic nanoparticle coordinates antibacterial and immunomodulatory functions to promote periodontal regeneration

Yufei Tang[1,#], Junhua Xu[2,#], Fang Zheng[3,#], Qin Xue[3], Sze Wing Cheung[1], Min Sun[2], Song Zhou[2], Yao He[3,*], Lin Xiang[1,*]

1 State Key Laboratory of Oral Diseases & National Center for Stomatology & National Clinical Research Center for Oral Diseases, West China Hospital of Stomatology, Sichuan University, Chengdu, Sichuan, China.

2 Biopharmaceutical Research Institute, West China Hospital, Sichuan University, Chengdu, Sichuan, China.

3 Stomatological Hospital of Chongqing Medical University & Chongqing Key Laboratory of Oral Diseases and Biomedical Sciences & Chongqing Municipal Key Laboratory of Oral Biomedical Engineering of Higher Education, Chongqing, China.

# The authors contributed equally to this work.

Correspondence

Lin Xiang, State Key Laboratory of Oral Diseases & National Center for Stomatology & National Clinical Research Center for Oral Diseases & Department of Oral Implantology, West China Hospital of Stomatology, Sichuan University, No 14th, 3rd section, Renmin South Road, Chengdu, 610041, China; Phone: +86 28 85503579; E-mail: dentistxiang@126.com.

Yao He, Stomatological Hospital of Chongqing Medical University & Chongqing Key Laboratory of Oral Diseases and Biomedical Sciences & Chongqing Municipal Key Laboratory of Oral Biomedical Engineering of Higher Education, Chongqing, China; E-mail: yaohe@hospital.cqmu.edu.cn.

Contents

1. **Characterization of nanoparticles by dynamic light scattering (Table S1)**
2. **Primer sequences for RT-qPCR (Table S2)**
3. **Fluorescent labeling of NR8383 cell membrane with PKH26 (Fig. S1)**
4. **Fluorescent labeling of rat neutrophil cell membrane with PKH67 (Fig. S2)**
5. **Hydrodynamic size distribution of NMm@MINO measured by DLS (Fig. S3)**
6. **Characterization of nanoparticle stability (Fig. S4)**
7. **Representative TEM images of GNP (Fig. S5)**
8. **Encapsulation efficiency and drug loading of GNP@MINO at different MINO:GNP feeding ratios. (Fig. S6)**
9. **CCK-8 assay for nanoparticle concentration screening (Fig. S7)**
10. **Representative light micrographs of RAW264.7 cells co-cultured with different materials (Fig. S8)**
11. **Representative light micrographs of MC3T3-E1 cells co-cultured with different materials (Fig. S9)**
12. **Statistical analysis of semi-quantified live/dead cell staining (Fig. S10)**
13. **Statistical analysis of cytoskeletal aspect ratio (Fig. S11)**
14. **Detection of intracellular reactive oxygen species (ROS) in P. gingivalis. (Fig. S12)**
15. **Antibacterial activity of NMm@MINO against *S. aureus* i*n vitro* (Fig. S13)**
16. **Semi-quantitative analysis of CD206 immunofluorescence staining (Fig. S14)**
17. **Semi-quantitative analysis of iNOS immunofluorescence staining (Fig. S15)**

18. A neutralization dose response of proinflammatory factors (IL-1β and IL-6) by nanoparticles (Fig. S16)

19. Microscopic and macroscopic view of alkaline phosphatase staining (Fig. S17)

20. Microscopic and macroscopic view of tartrate-resistant acid phosphatase staining (Fig. S18)

21. Representative fluorescence images and semi-quantification of C-fos (Fig. S19)

22. Cytocompatibility of NMm@MINO with rat periodontal ligament fibroblasts (Fig. S20)

23. Retention profile of locally administered NMm@MINO in gingival sites (Fig. S21)

24. *In vivo* preliminary experimental results (Fig. S22)

25. Anti-inflammatory effect of NMm@MINO *in vivo*（Fig. S23）

26. Gum bleeding index of the indicated groups (Fig. S24)

27. Plaque staining for periodontal tissue (Fig. S25)

28. Soft tissue regeneration effect of NMm@MINO *in vivo* (Fig. S26)

29. Effects of NMm@MINO on osteogenic and osteoclastic functions *in vivo* (Fig. S27)

30. Hematological examination analysis (Fig. S28)

31. Biosafety assessment of NMm@MINO *in vivo* (Fig. S29)

32. Weight changes in rats of different groups (Fig. S30)

Table S1 Characterization of nanoparticles by dynamic light scattering (DLS).

	Size (nm)	Zeta potential (mV)	Polydispersity
GNP	218.17 ± 15.06	-21.68 ± 2.52	0.05982 ± 0.05342
GNP@MINO	236.67 ± 15.97	-20.42 ± 4.24	0.03290 ± 0.03028
NMm	258.67 ± 21.34	-12.52 ± 2.47	0.03708 ± 0.01763
NMm@MINO	261.53 ± 11.59	-15.30 ± 5.14	0.06493 ± 0.01977

Table S2 Primer sequences for RT-qPCR.

Primer	Forward primer 5′-3′	Reverse primer 5′-3′
In vitro		
Gapdh	AAGGCCGGGGCCCACTTGAA	GGACTGTGGTCATGAGCCCTTCCA
Arg1	CTCCAAGCCAAAGTCCTTAGAG	GGAGCTGTCATTAGGGACATCA
Cd86	TCAATGGGACTGCATATCTGCC	GCCAAAATACTACCAGCTCACT
Mrc1	CTCTGTTCAGCTATTGGACGC	TGGCACTCCCAAACATAATTTGA
Il1b	GAAATGCCACCTTTTGACAGTG	TGGATGCTCTCATCAGGACAG
Nos2	GTTCTCAGCCCAACAATACAAGA	GTGGACGGGTCGATGTCAC
Tgfb1	GAGCCCGAAGCGGACTACTA	TGGTTTTCTCATAGATGGCGTTG
Tnf	CAGGCGGTGCCTATGTCTC	CGATCACCCCGAAGTTCAGTAG
Fos	CGAAGGGAACGGAATAAGATG	GCTGCCAAAATAAACTCCAG
Ctsk	GAAGAAGACTCACCAGAAGCAG	TCCAGGTTATGGGCAGAGATT
Mmp9	CGTCGTGATCCCCACTTACT	AACACACAGGGTTTGCCTTC
Tnfrsf11a	CTAATCCAGGGAAGCAAAT	GACACGGGCATAGAGTCAGTTC
Acp5	CTGGAGTGCACGATGCCAGCGACA	TCCGTGCTCGGCGATGCACCAGA
Alpl	AACCCAGACACAAGCATTCC	GCCTTTGAGGTTTTTGGTCA
Runx2	GAGGCCGCCGCACGACAACCG	CTCCGGCCCACAAATCTCAGA

Spp1	CCCGGTGAAAGTGACTGATT	TTCTTCAGAGGACACAGCATTC
In vivo		
Gapdh	GGCACAGTCAAGGCTGAGAATG	ATGGTGGTGAAGACGCCAGTA
Il6	AGCCAGAGTCATTCAGAGCAA	GGTCCTTAGCCACTCCTTCTGT
Crp	CAGGAGCAGGACTCGTATGG	CGCCAGTTCAAAACATTGGGG
Il1b	CCCTGAACTCAACTGTGAAATAG	CCCAAGTCAAGGGCTTGGAA
Tnfsf11	AAGATGGCTTCTATTACCTGT	ATTTTGATGCTGGTTTTAACGA
Acp5	ATGACAAGAGGTTCCAGGAGA	ACGGTAATAAGGGCTGGGGA
Tnfrsf11b	AACCGCACCCACAACCGA	CACCTGAGAAGAACCCATCCG
Runx2	CATGGCCGGGAATGATGAG	TGTGAAGACCGTTATGGTCAAAGTG
Col1a1	CACTGCAAGAACAGCGTAGC	AAGTTCCGGTGTGACTCGTG

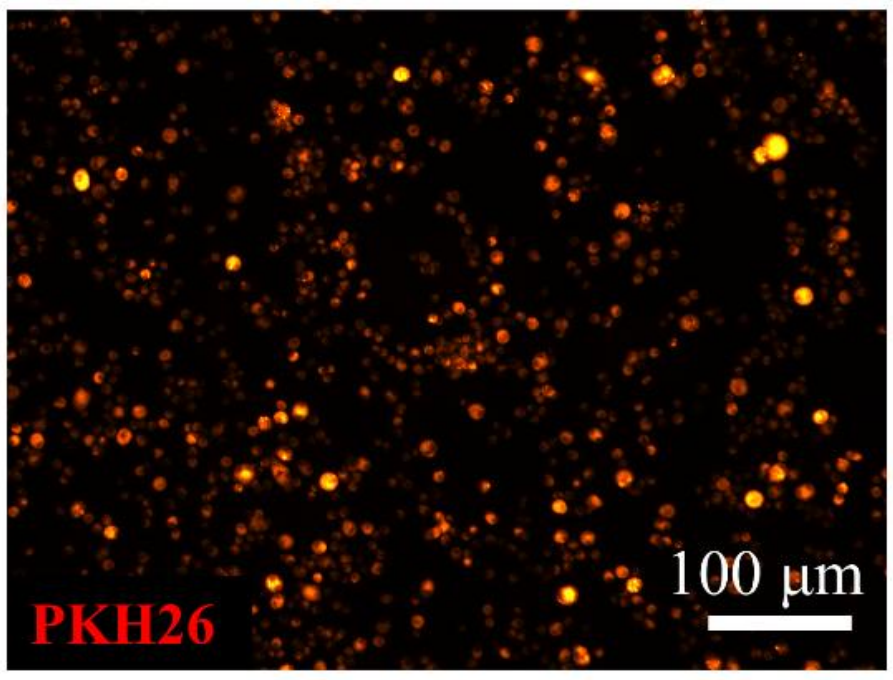

Fig. S1 Fluorescent labeling of NR8383 cell membrane with PKH26 (Red). Scale bar = 100 μm.

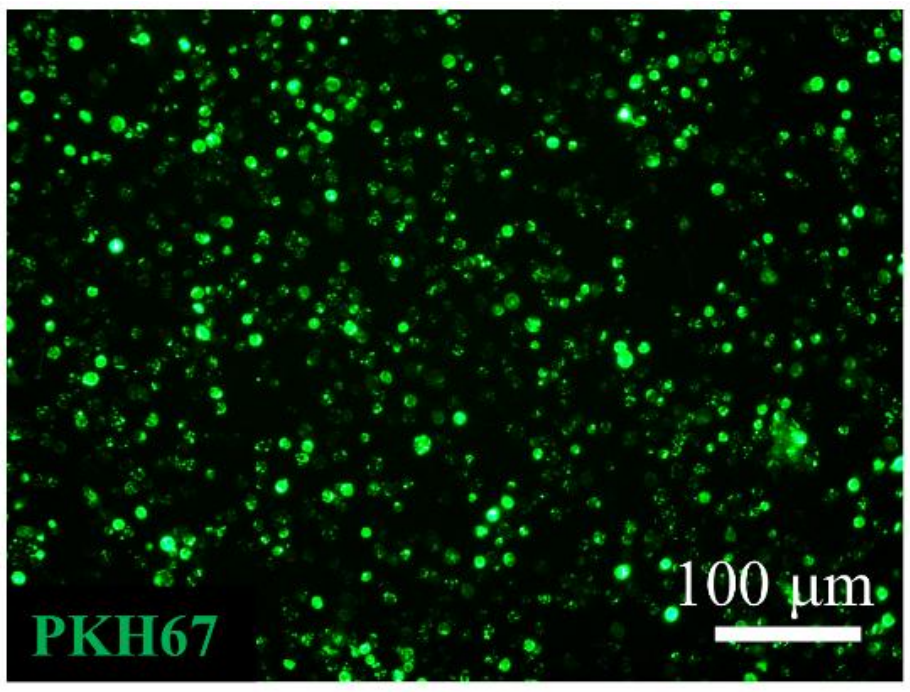

Fig. S2 Fluorescent labeling of rat neutrophil cell membrane with PKH67 (Green). Scale bar = 100 μm.

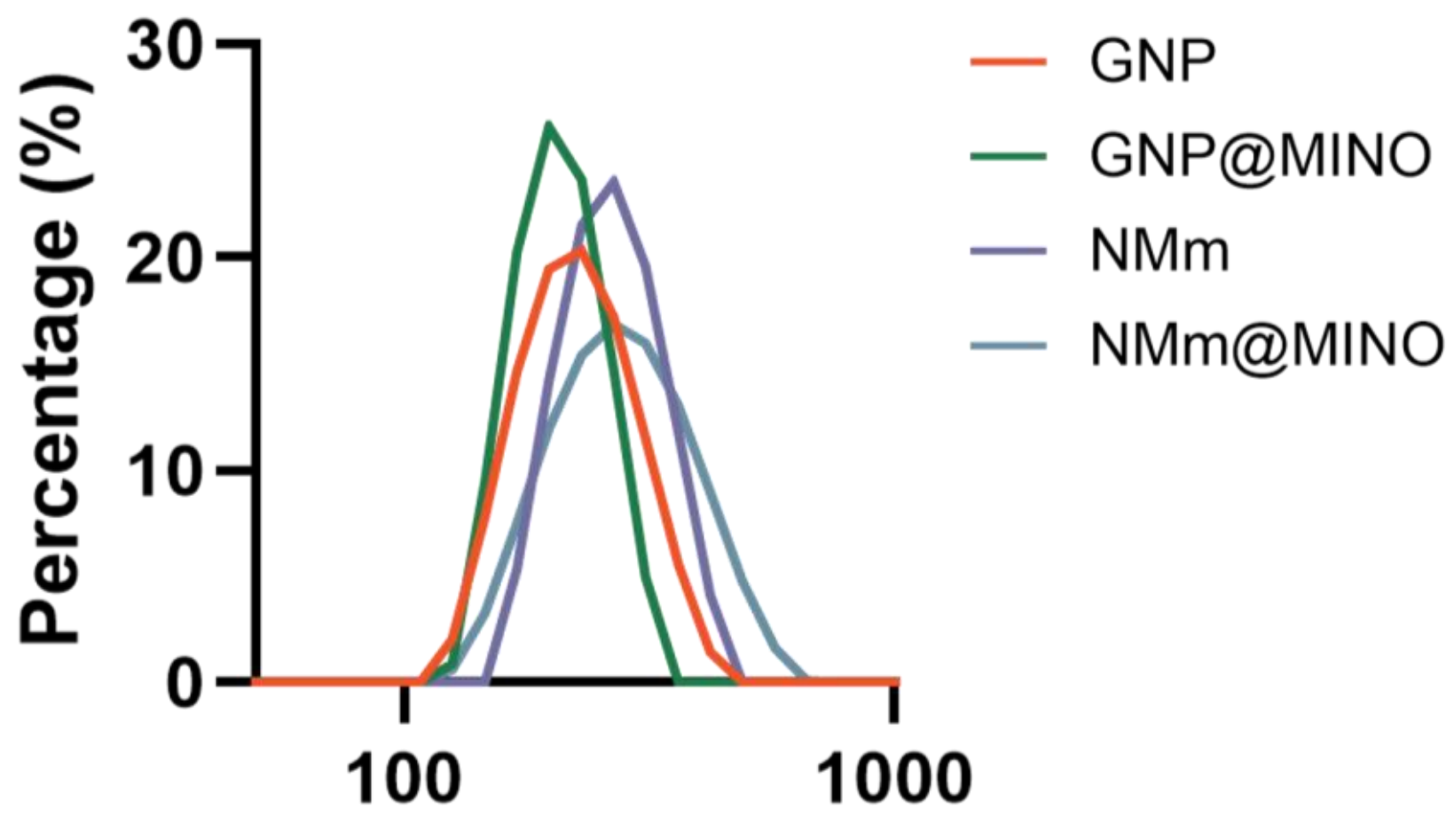

Fig. S3 Hydrodynamic size distribution of NMm@MINO measured by DLS.

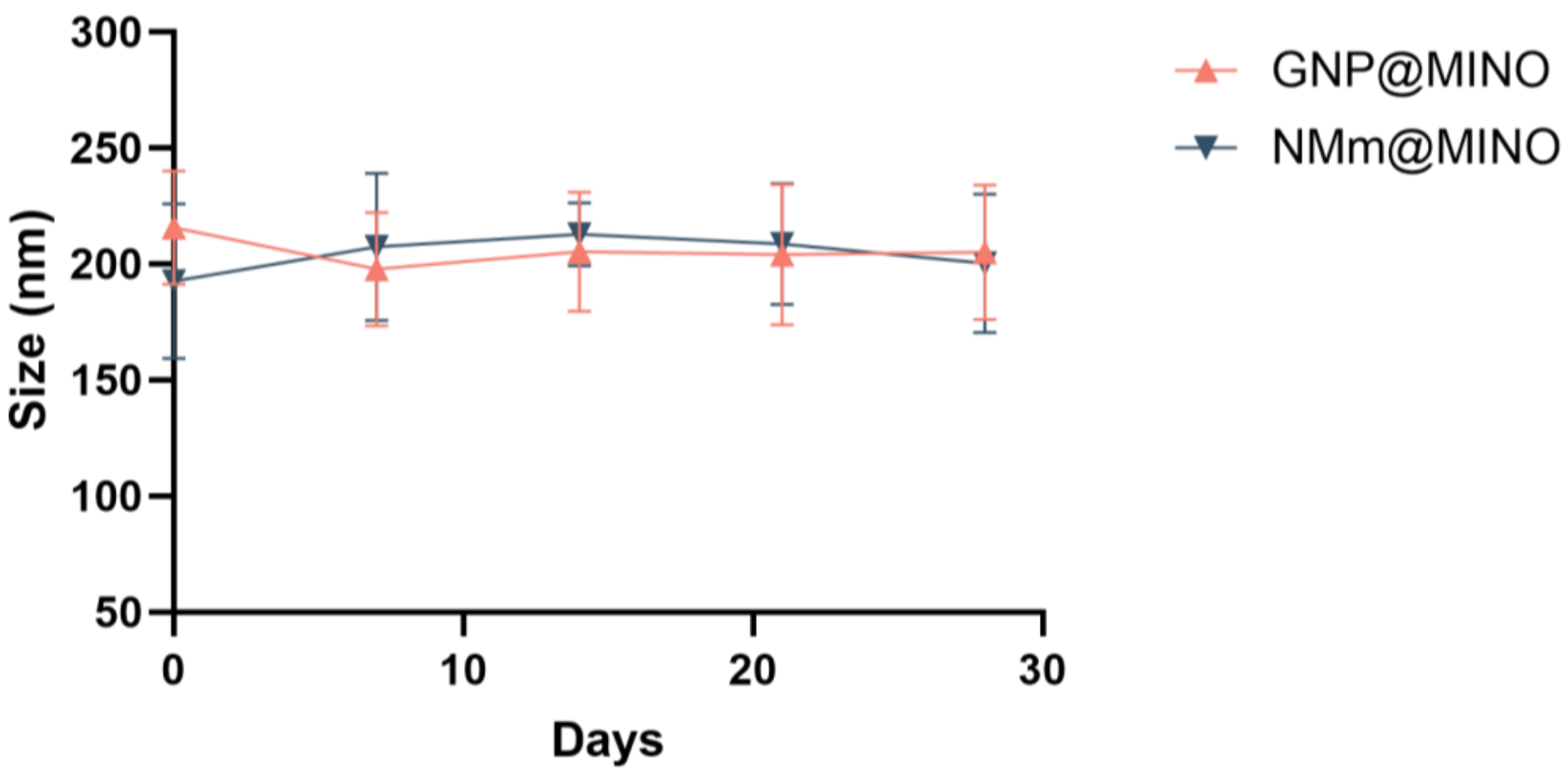

Fig. S4 Characterization of nanoparticles stability.

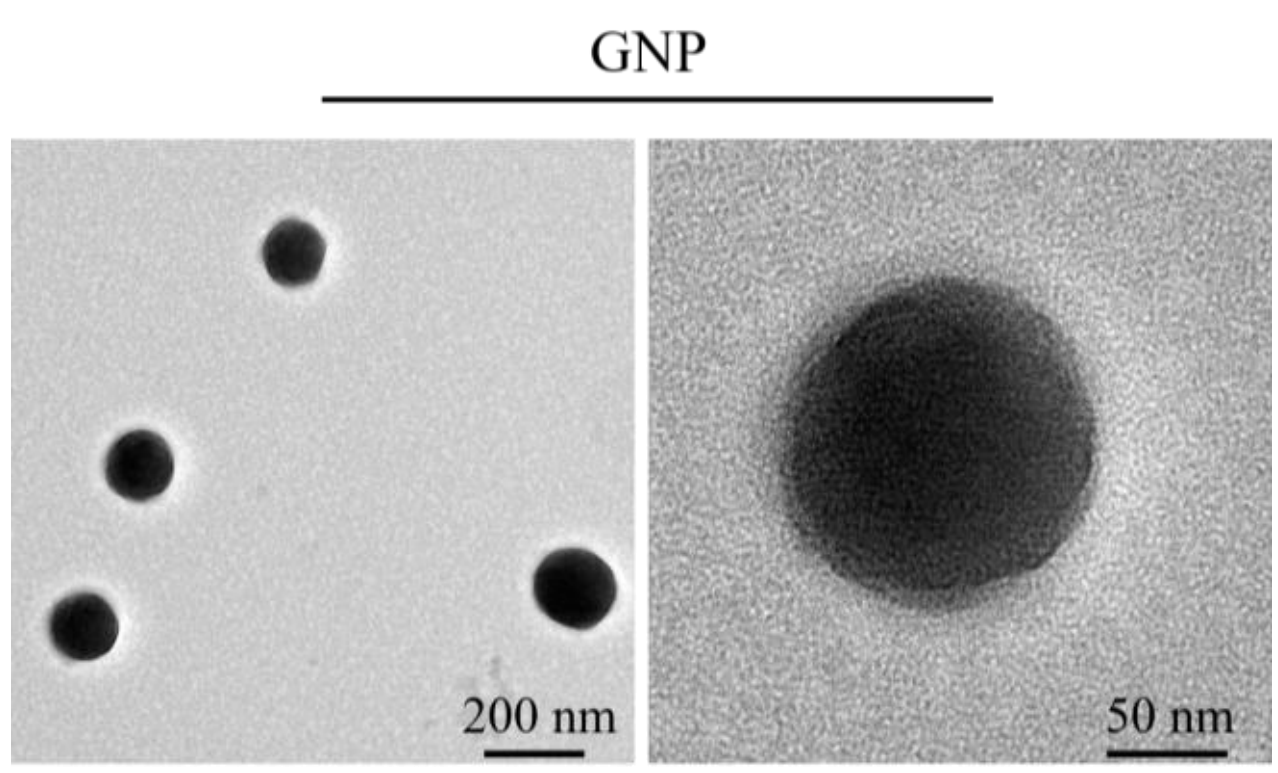

Fig. S5 Representative TEM images of GNP. Scale bars = 200 nm (left), 50 nm (right).

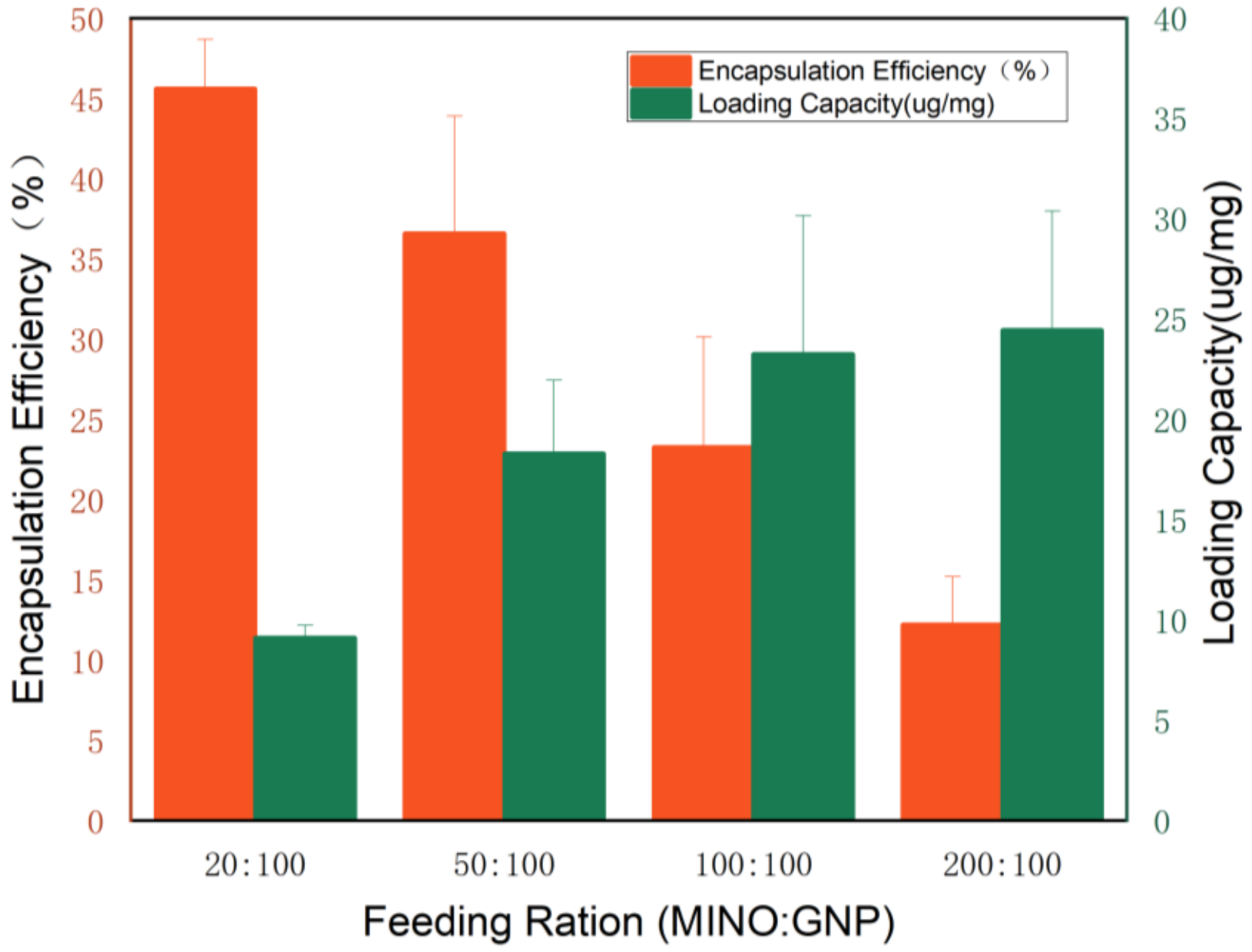

Fig. S6 Encapsulation efficiency and drug loading of GNP@MINO at different MINO:GNP feeding ratios. Gelatin nanoparticles were incubated with minocycline solutions at feeding ratios of 20:100, 50:100, 100:100, and 200:100 (w/w). Unbound drug in the supernatant was quantified by HPLC to calculate encapsulation efficiency (EE) and loading capacity (LC). Data are presented as mean ± SD (n = 3).

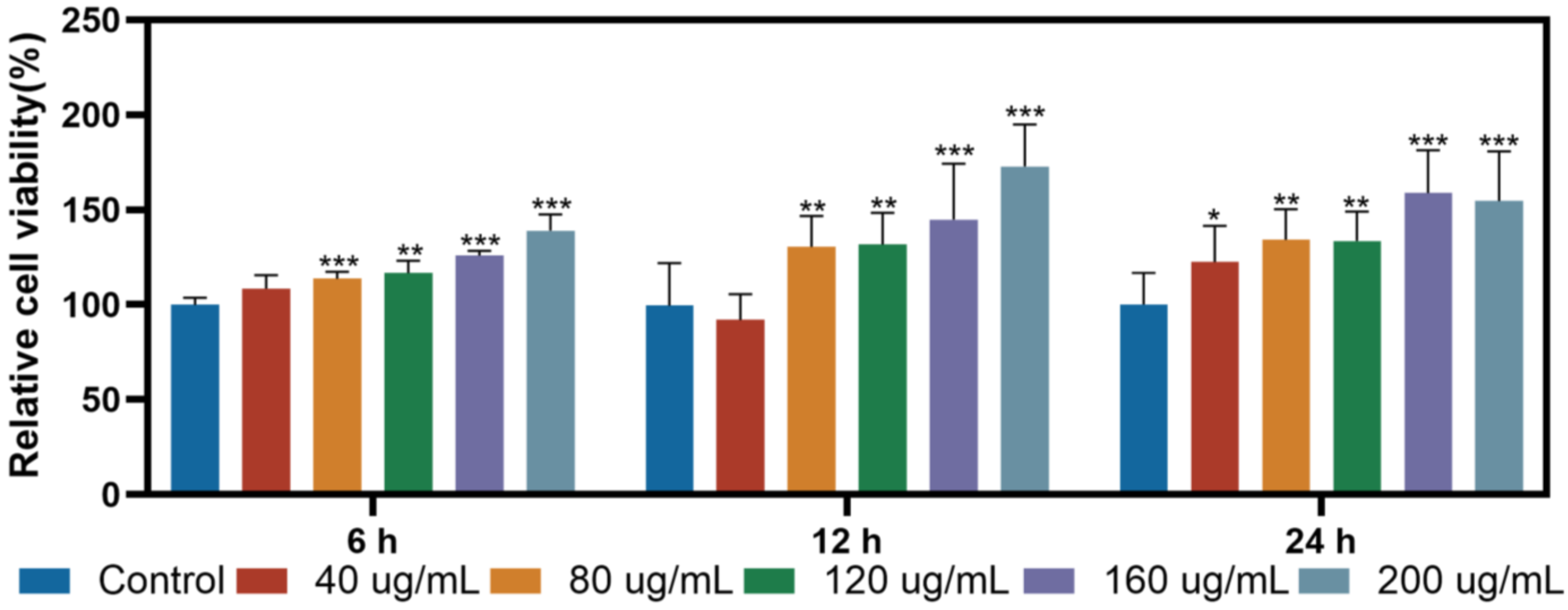

Fig. S7 CCK-8 assay for nanoparticle concentration screening, n = 7. Data are presented as mean ± SD, * $p < 0.05$, ** $p < 0.01$, *** $p < 0.001$.

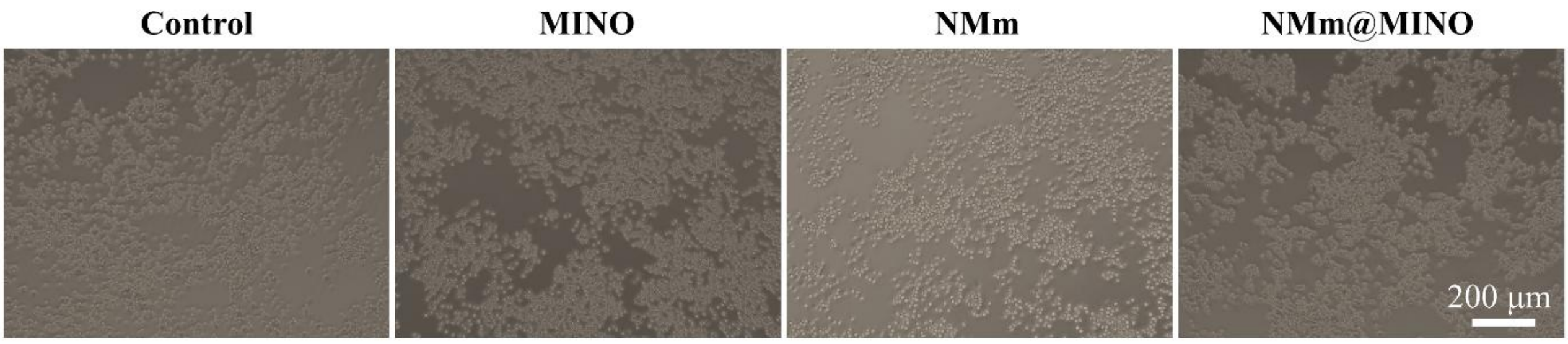

Fig. S8 Representative light micrographs of RAW264.7 cells co-cultured with different materials. Scale bar = 200 μm.

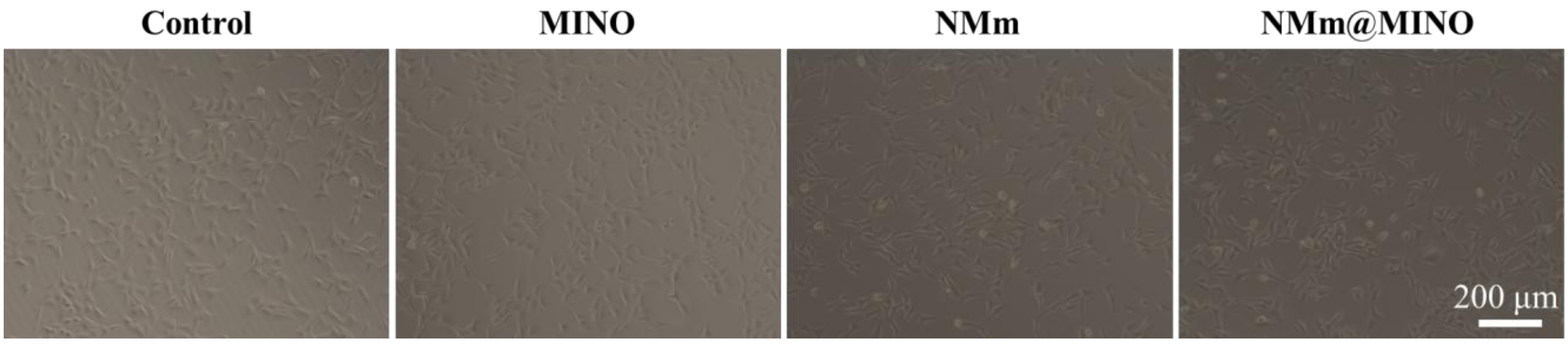

Fig. S9 Representative light micrographs of MC3T3-E1 cells co-cultured with different materials. Scale bar = 200 μm.

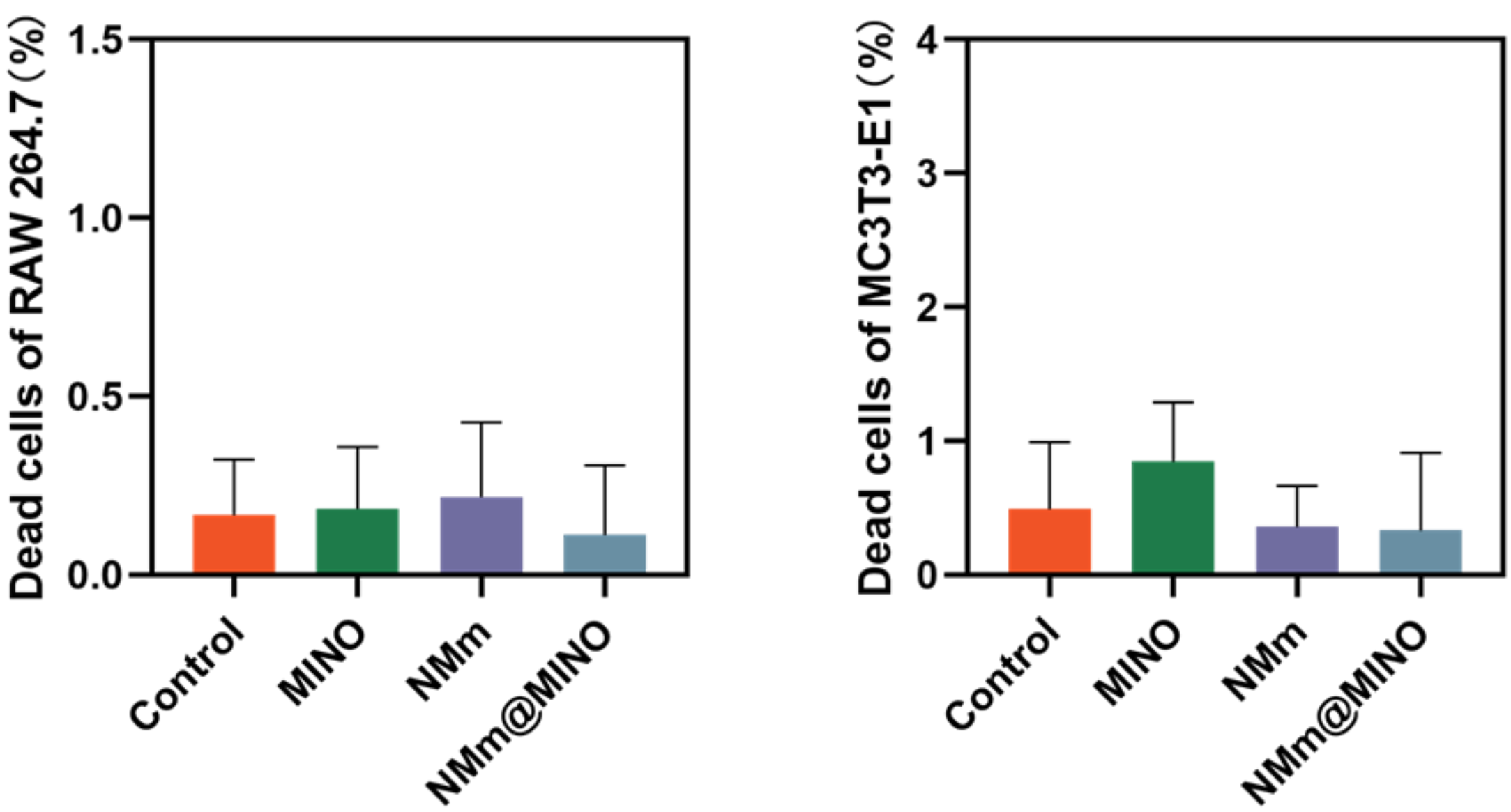

Fig. S10 Statistical analysis of semi-quantified live/dead cell staining. Data are presented as mean ± SD (n = 3).

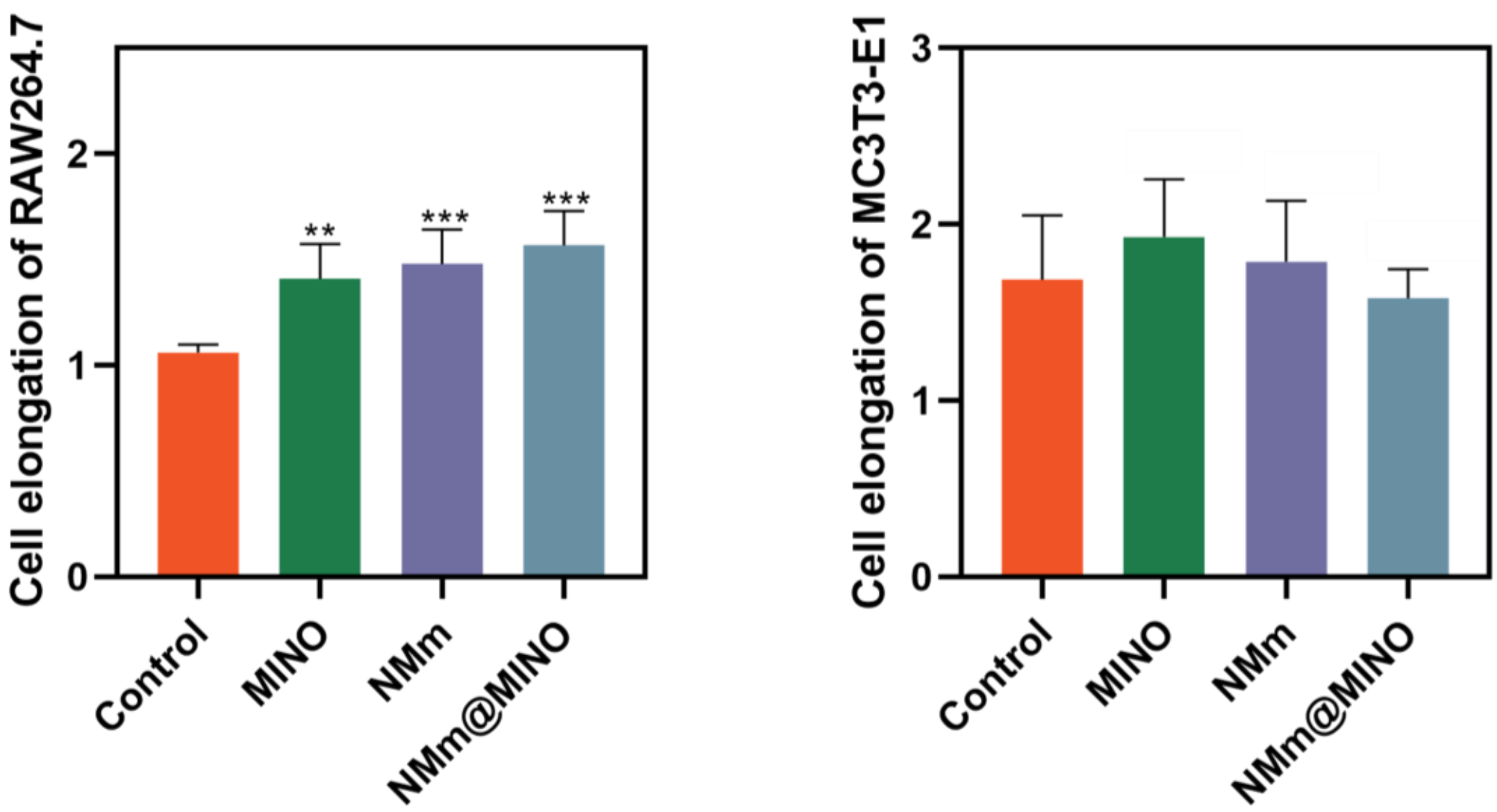

Fig. S11 Statistical analysis of cytoskeletal aspect ratio. Data are presented as mean ± SD (n = 3), * $p < 0.05$, ** $p < 0.01$.

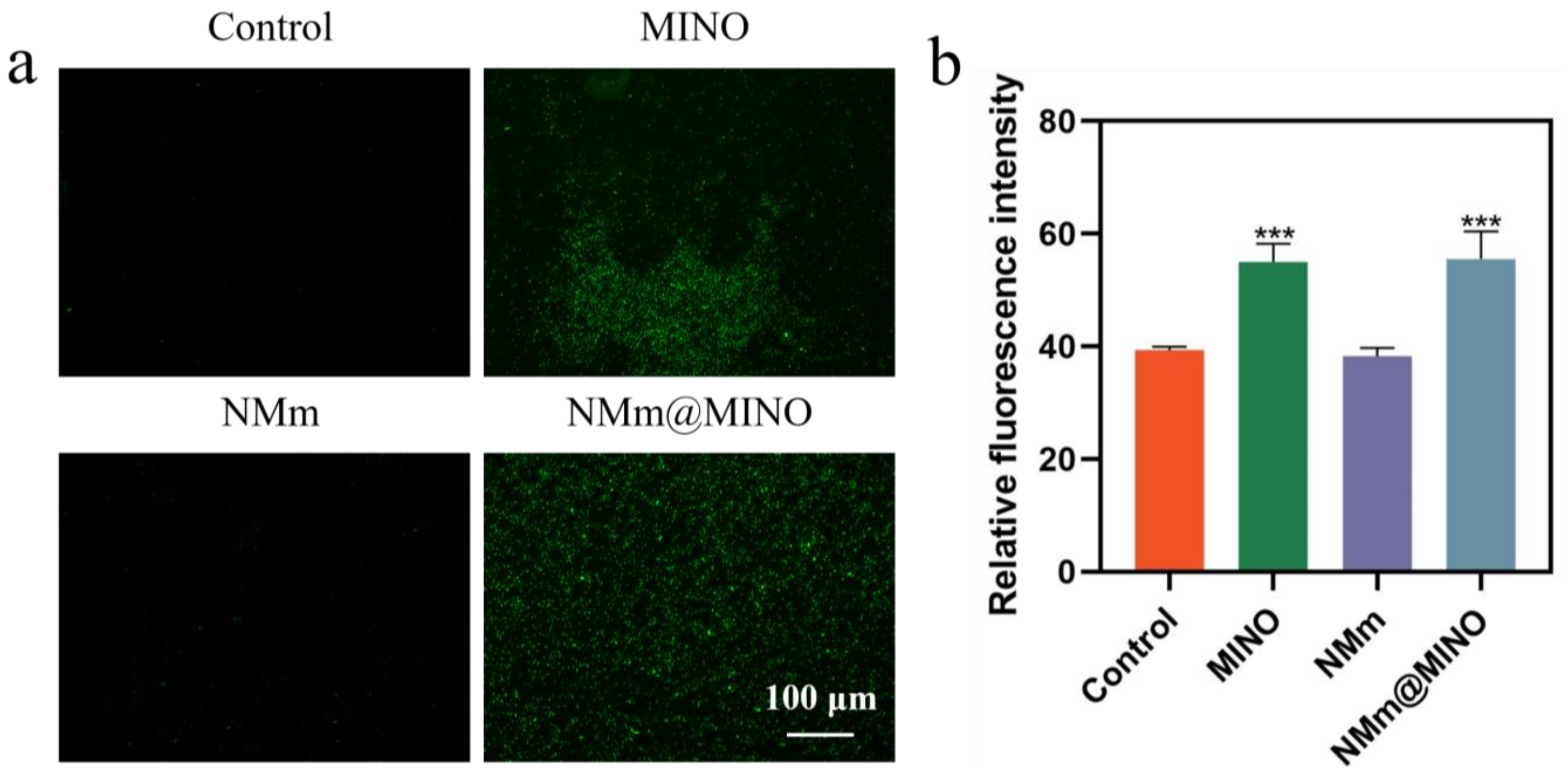

Fig. S12 Detection of intracellular reactive oxygen species (ROS) in *P. gingivalis*. (a) Representative fluorescence images of ROS. Scale bar = 100 μm; (b) Semi-quantitative analysis of intracellular ROS levels in *P. gingivalis*. Data are presented as mean ± SD (n = 3), *** $p < 0.001$.

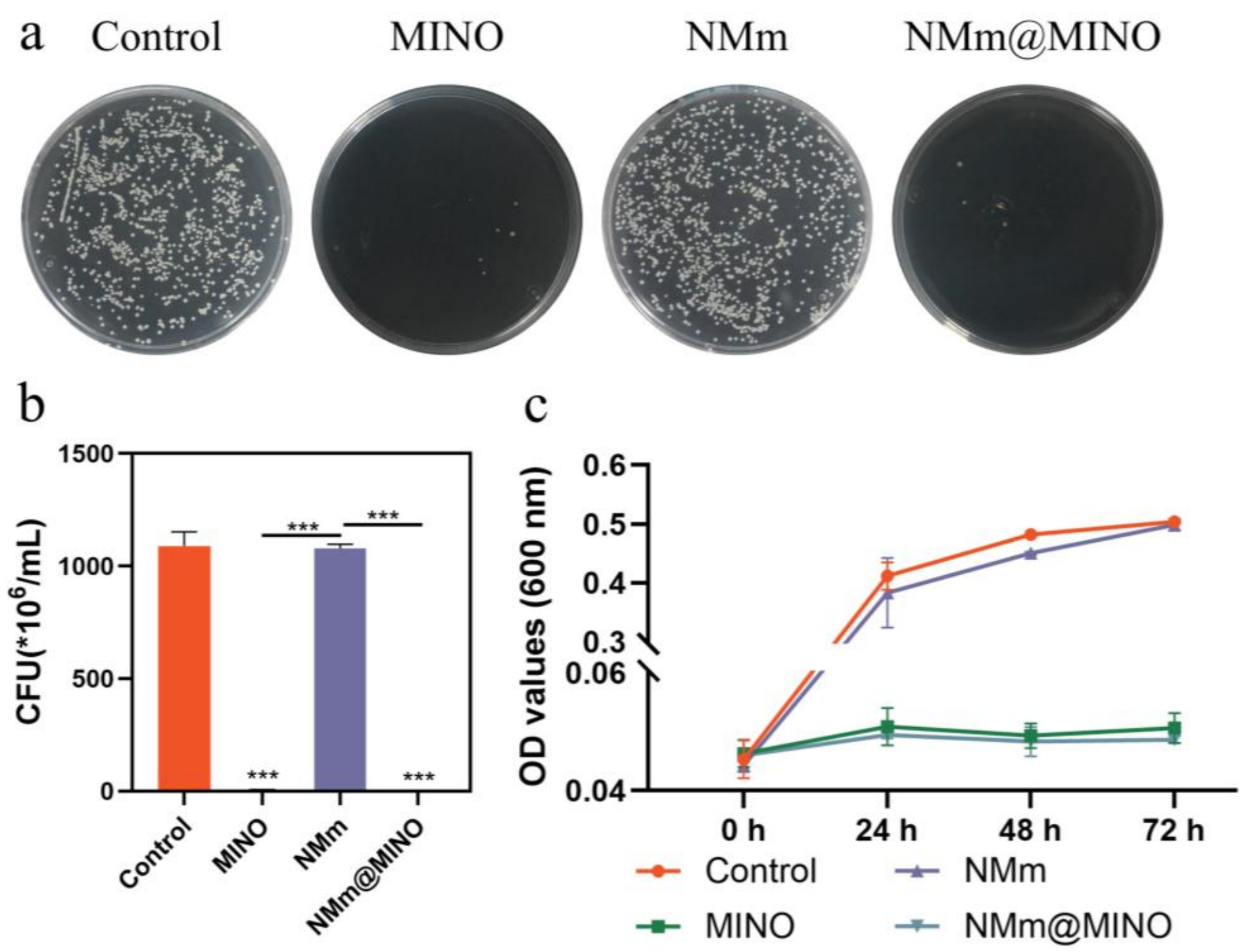

Fig. S13 Antibacterial activity of NMm@MINO against *S. aureus in vitro*. (a) Representative images of *S. aureus* colonies on agar plates; (b) Quantification of viable bacteria by colony-forming unit counting; (c) Growth kinetics of *S. aureus* monitored by continuous optical density measurement at 600 nm (OD600). Data are presented as mean ± SD, n = 3, compared with control group, *** $p < 0.001$.

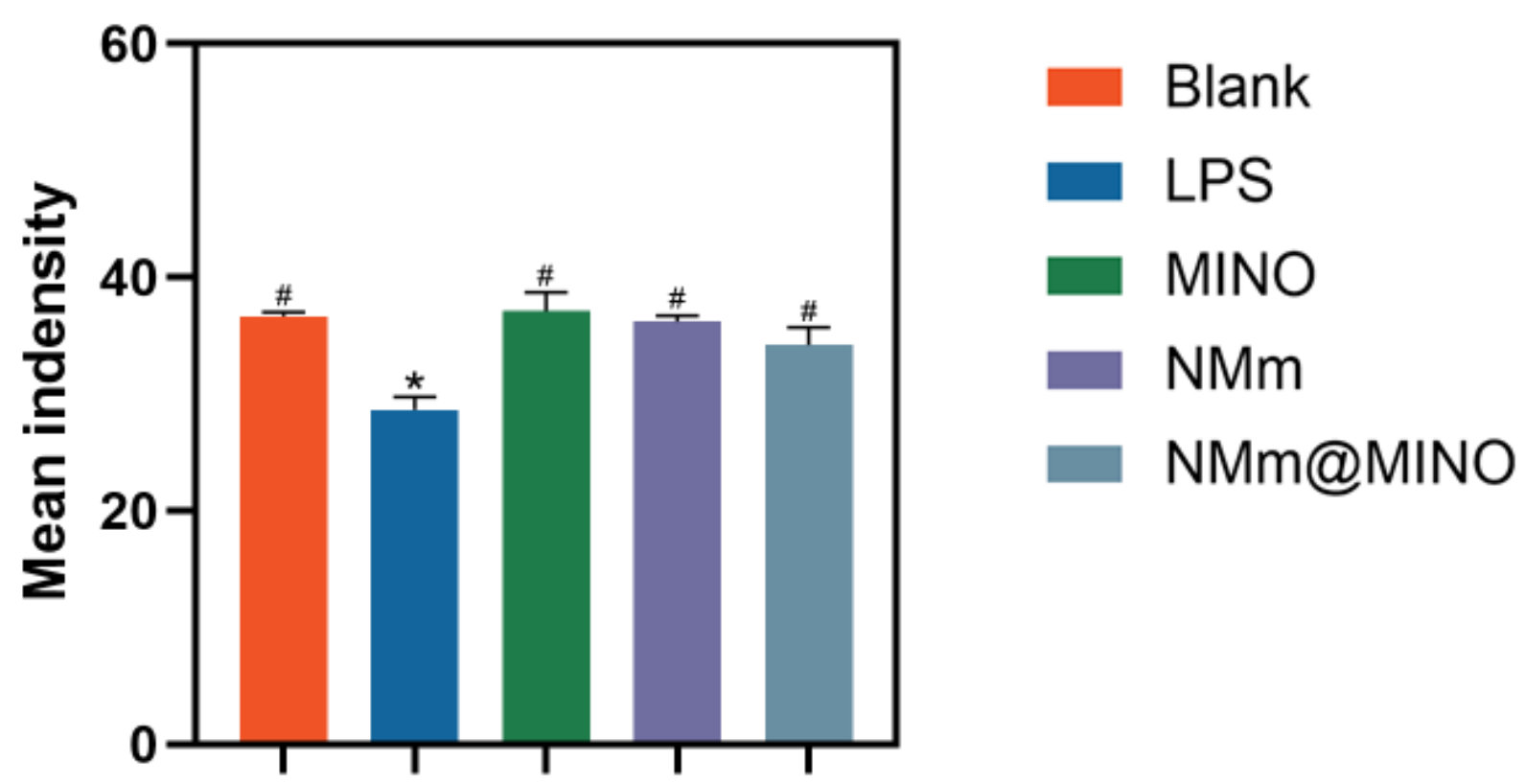

Fig. S14 Semi-quantitative analysis of CD206 immunofluorescence staining. Data are presented as mean ± SD, n = 3, compared with control group, * $p < 0.05$; compared with LPS group, $^{\#}$ $p < 0.05$.

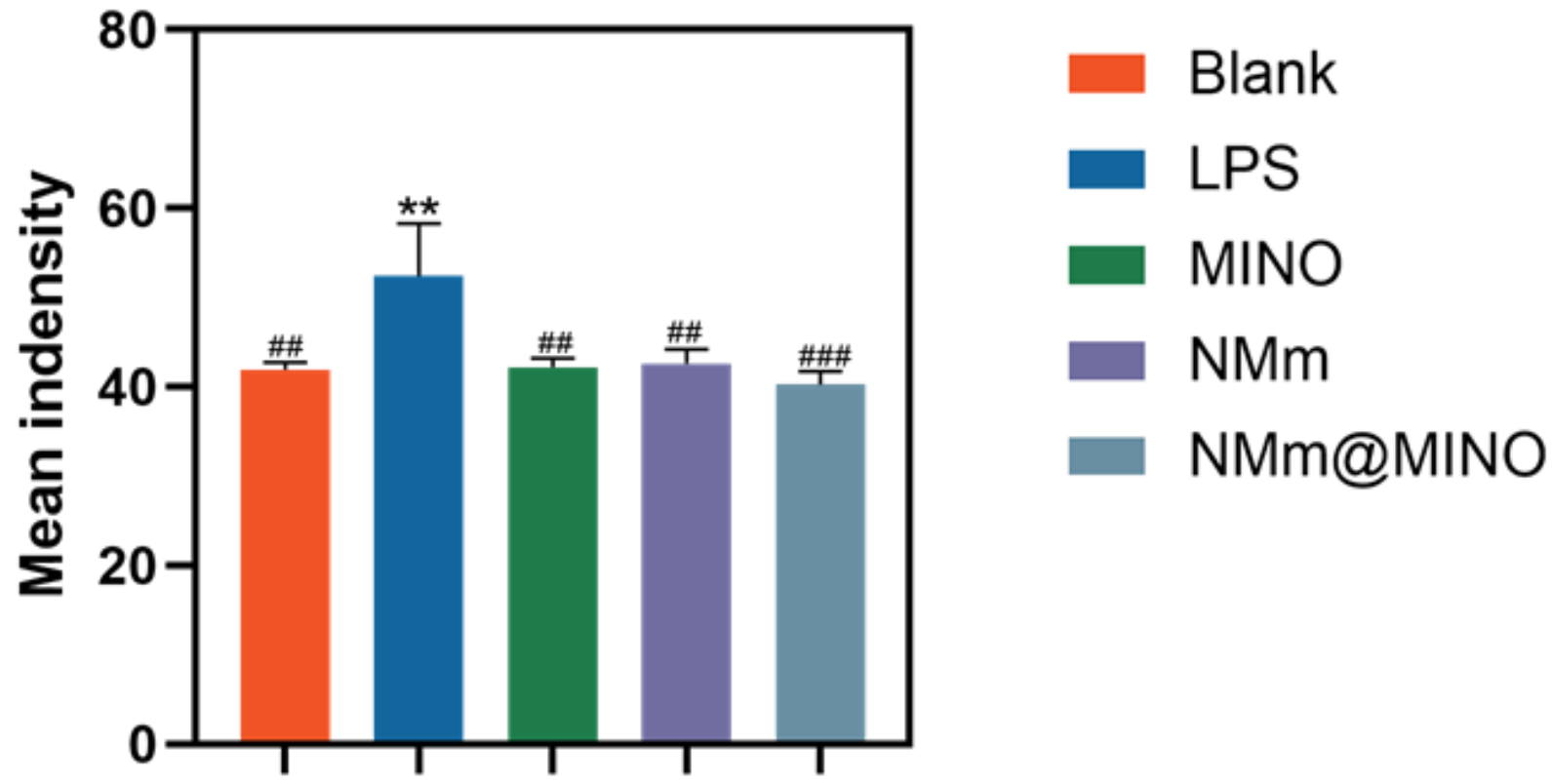

Fig. S15 Semi-quantitative analysis of iNOS immunofluorescence staining. Data are presented as mean ± SD, n = 3, compared with control group, ** $p < 0.01$; compared with LPS group, ## $p < 0.01$, ### $p < 0.001$.

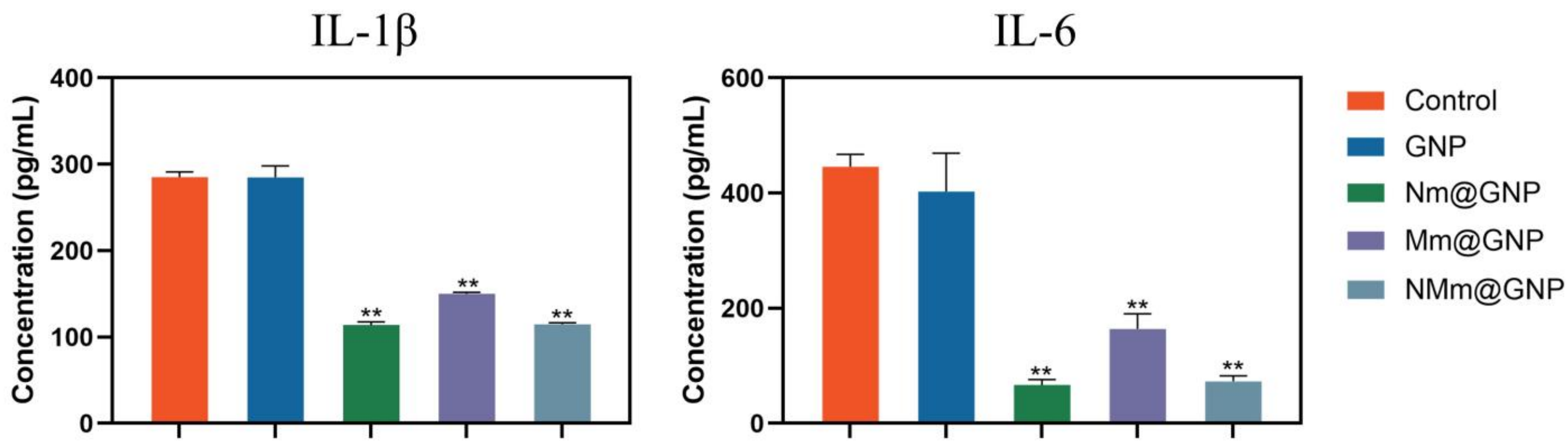

Fig. S16 Neutralization dose response of proinflammatory factors (IL-1β and IL-6) by nanoparticles. Data are presented as mean ± SD, n = 3, ** $p < 0.01$.

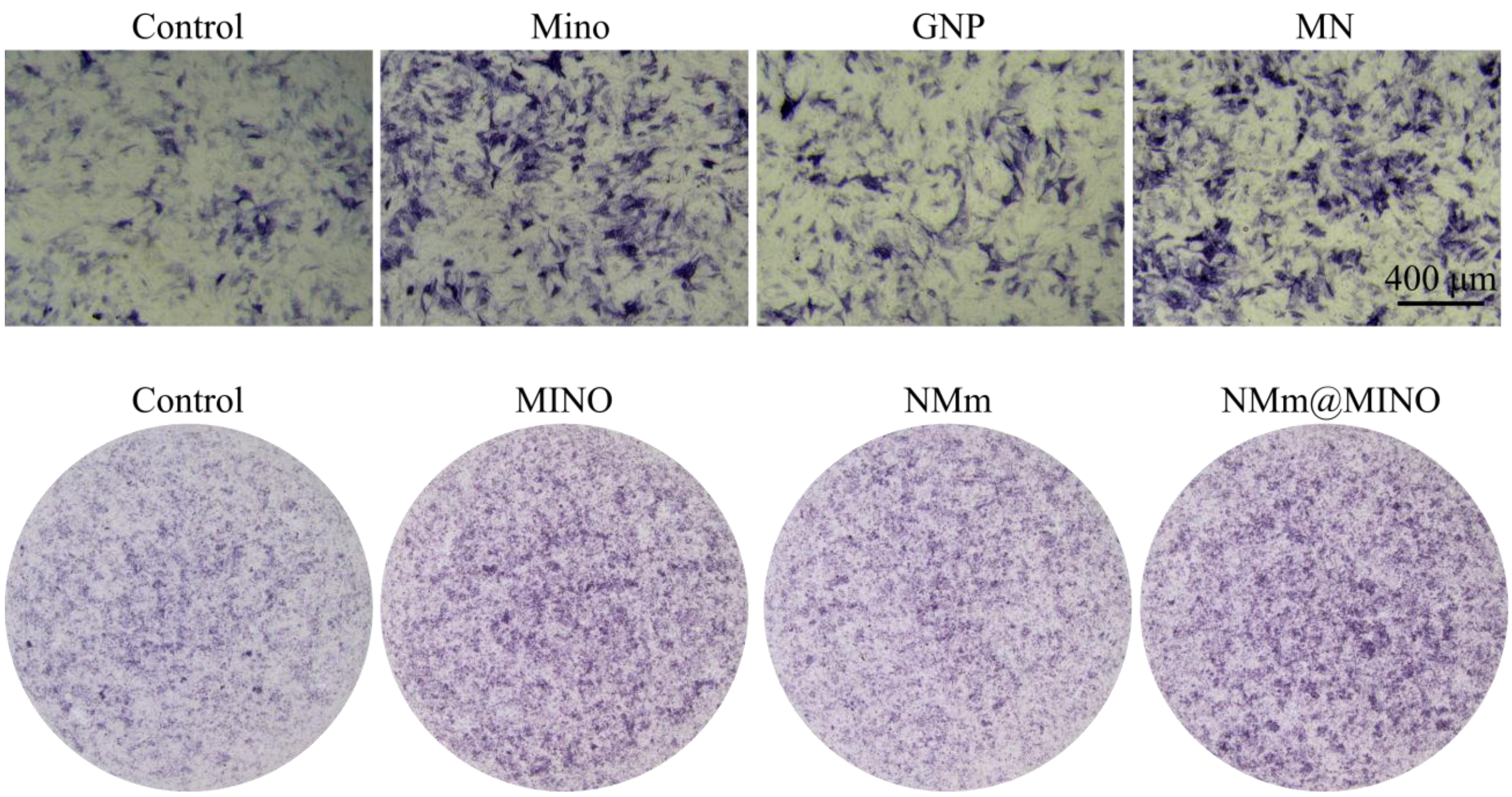

Fig. S17 Microscopic and macroscopic view of alkaline phosphatase (ALP) staining. Scale bar = 400 μm.

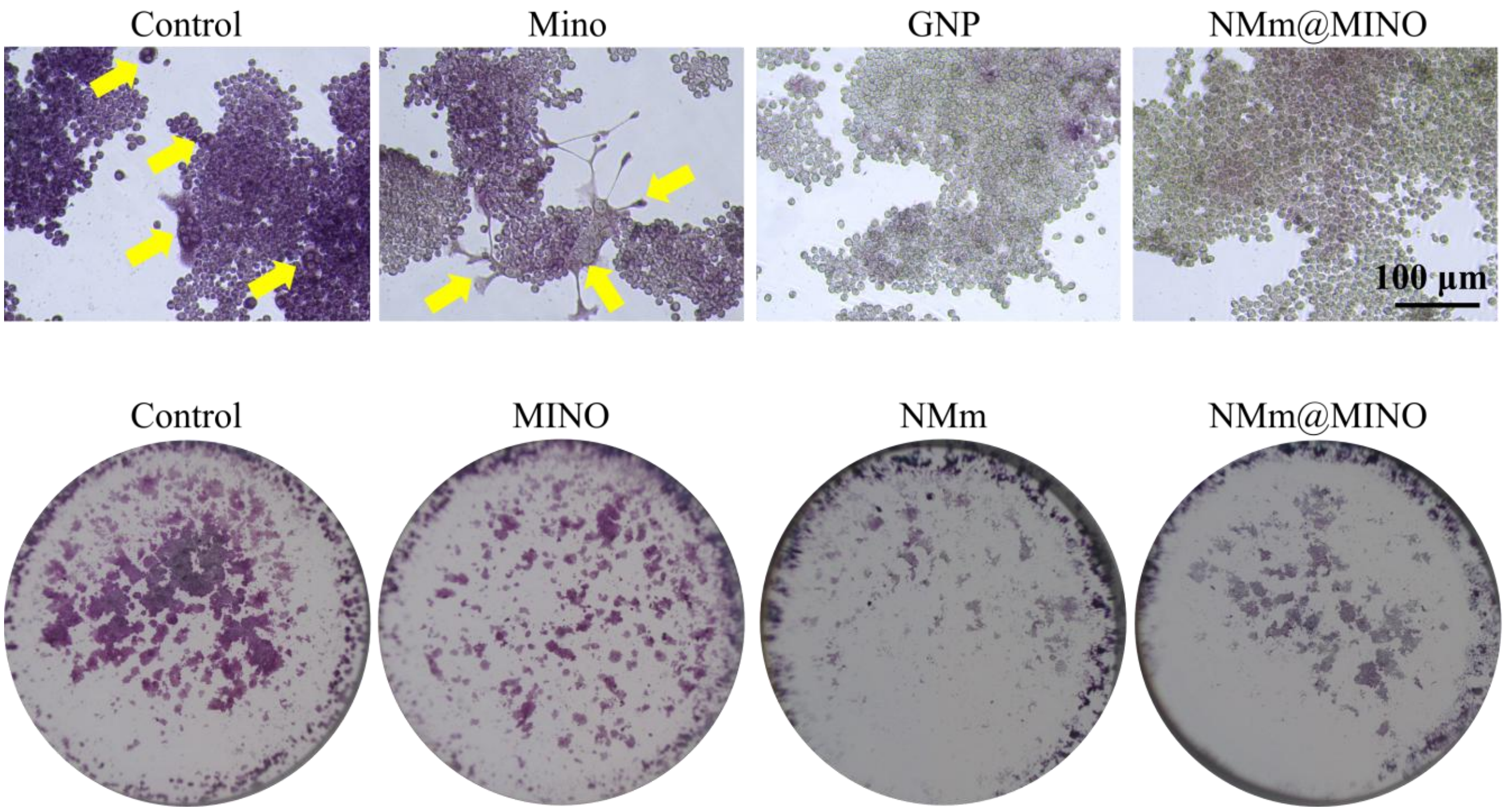

Fig. S18 Microscopic and macroscopic view of tartrate-resistant acid phosphatase (TRAP) staining (yellow arrows indicating characteristic areas of cell fusion). Scale bar = 100 μm.

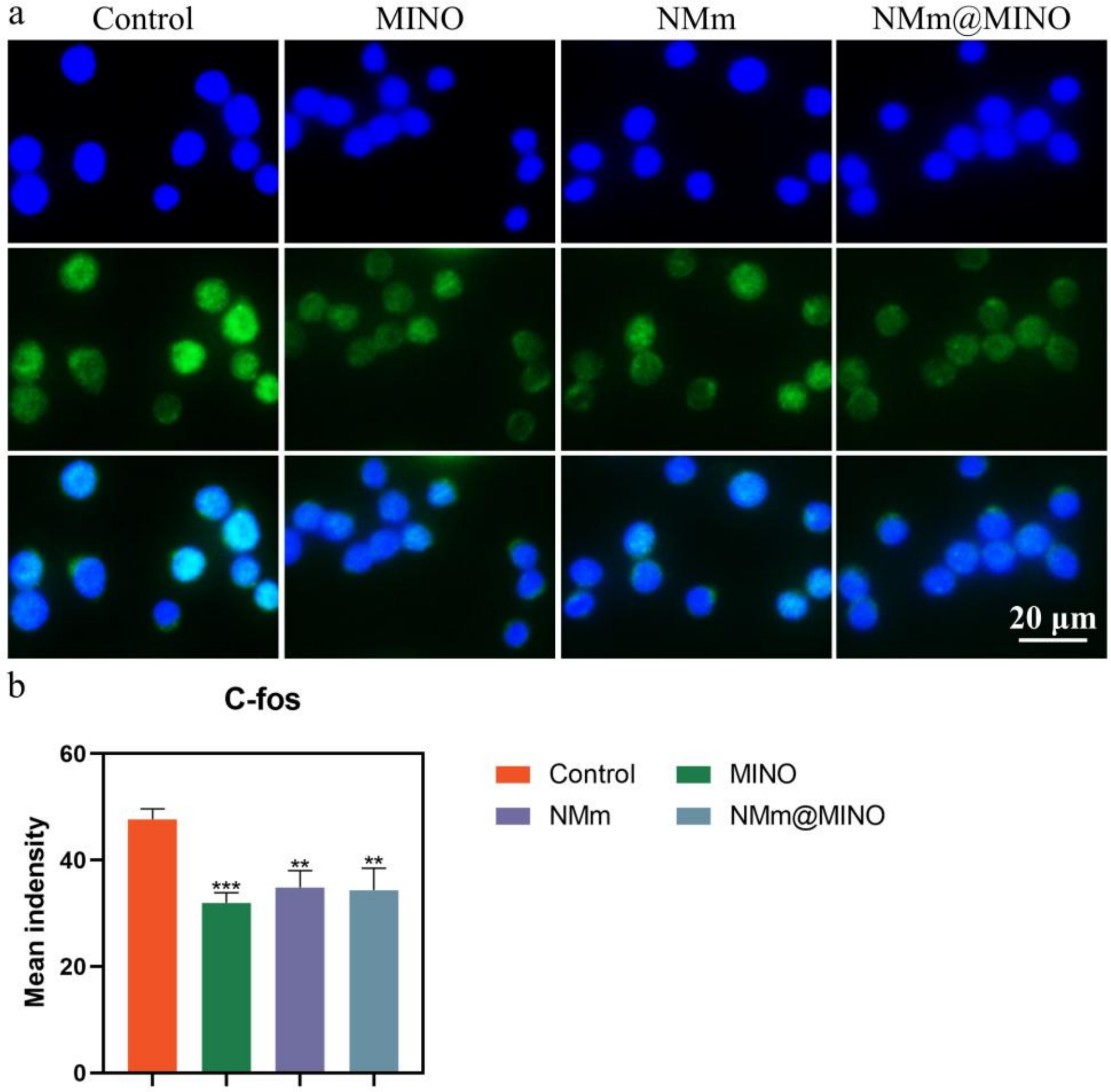

Fig. S19 Representative fluorescence images (**a**) and semi-quantification (**b**) of c-fos. Scale bar = 20 μm. Data are presented as mean ± SD, n = 3, ** $p < 0.01$, *** $p < 0.001$.

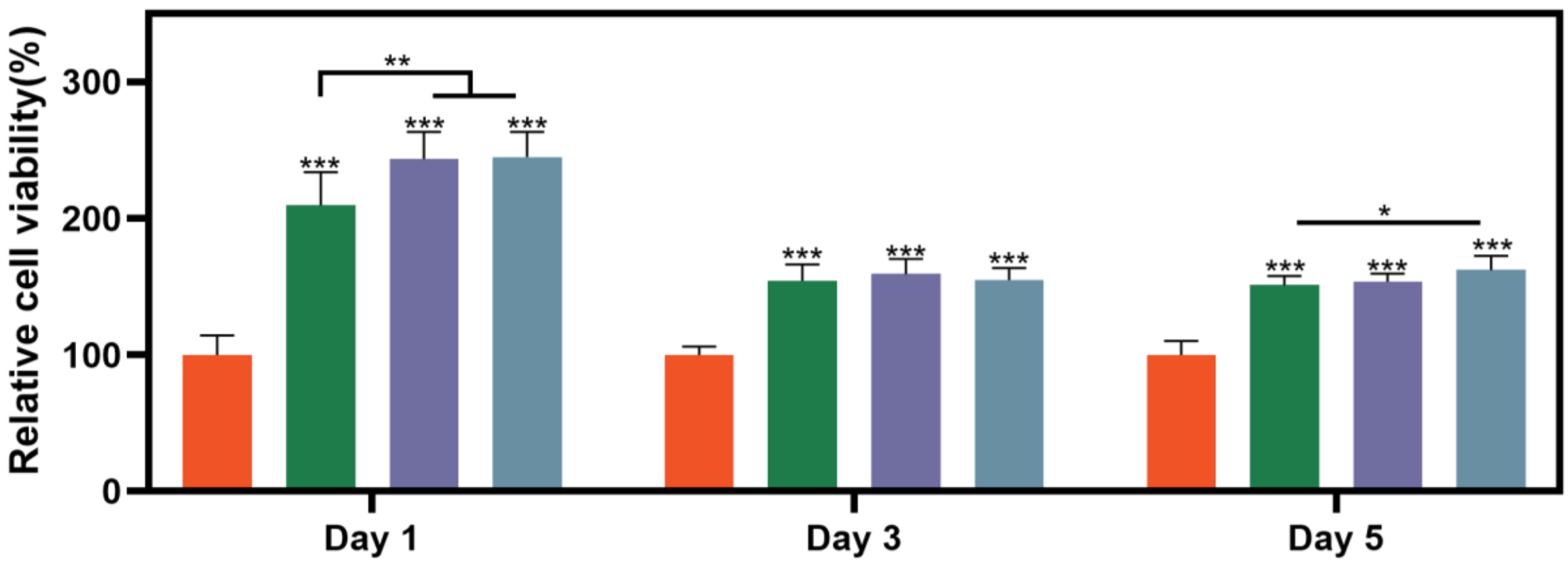

Fig. S20 Cytocompatibility of NMm@MINO with rat periodontal ligament fibroblasts (rPDLFs). Data are presented as mean ± SD, n = 7, * $p < 0.05$, ** $p < 0.01$, *** $p < 0.001$.

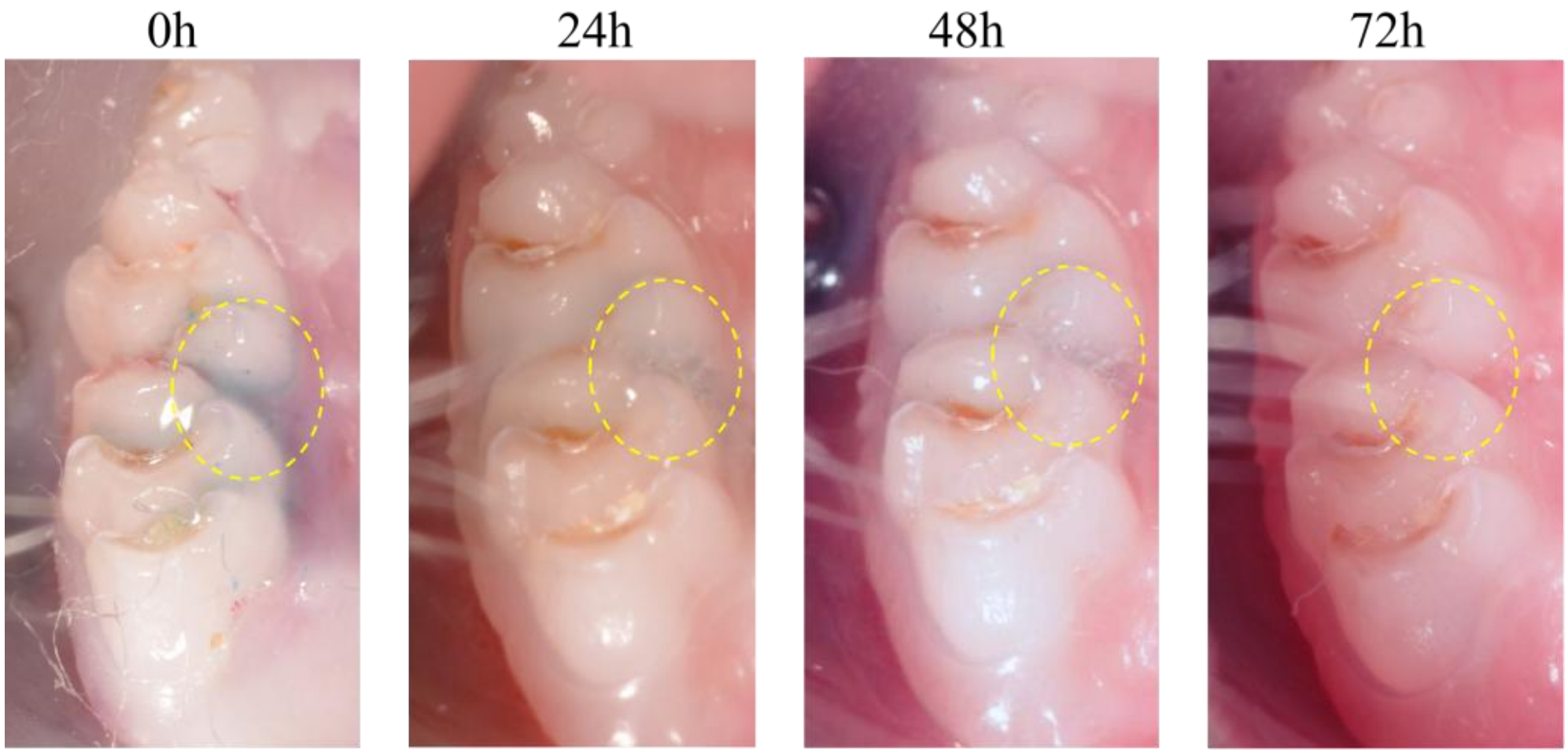

Fig. S21 Retention profile of locally administered NMm@MINO in gingival sites. The yellow dashed circles indicate the gingival sites where NMm@MINO was retained, showing the temporal changes in drug retention at 0 h, 24 h, 48 h, and 72 h after local administration.

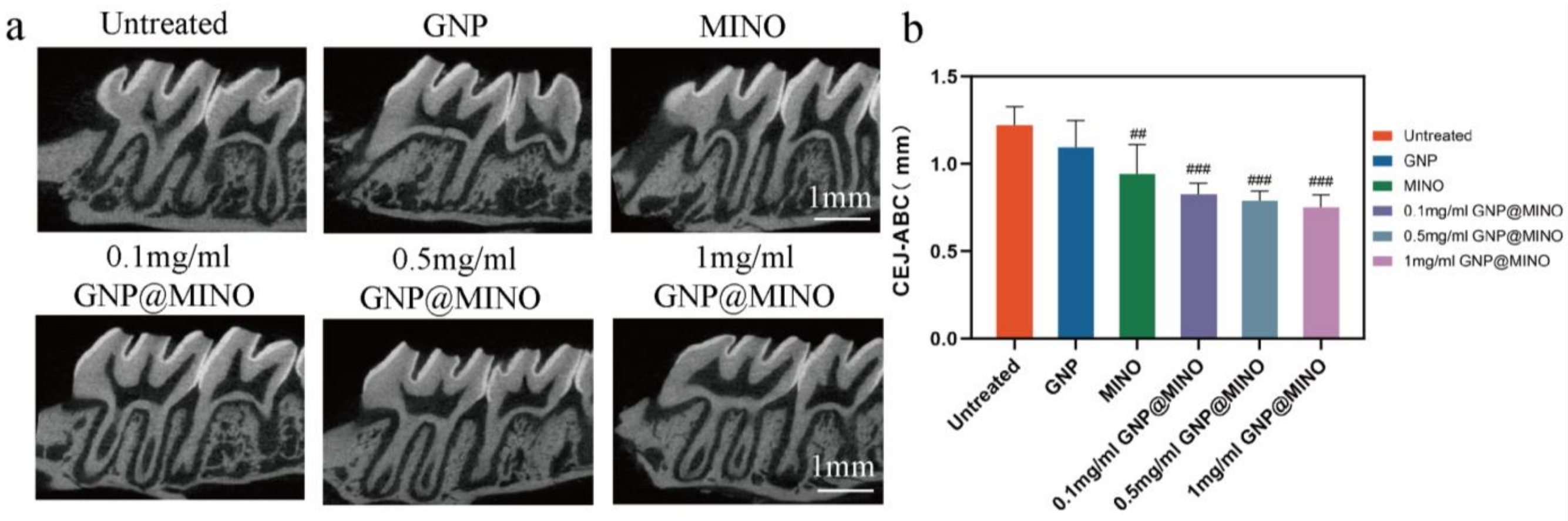

Fig. S22 *In vivo* preliminary experimental results. Representative images of micro-CT (**a**), including the cementoenamel junction (**b**) of alveolar bone height after treatment with minocycline nanogel at different concentration gradients. Scale bar = 1 mm. Data are presented as mean ± SD, n = 4, compared with Untreated group, $^{\#\#}$ $p < 0.01$, $^{\#\#\#}$ $p < 0.001$.

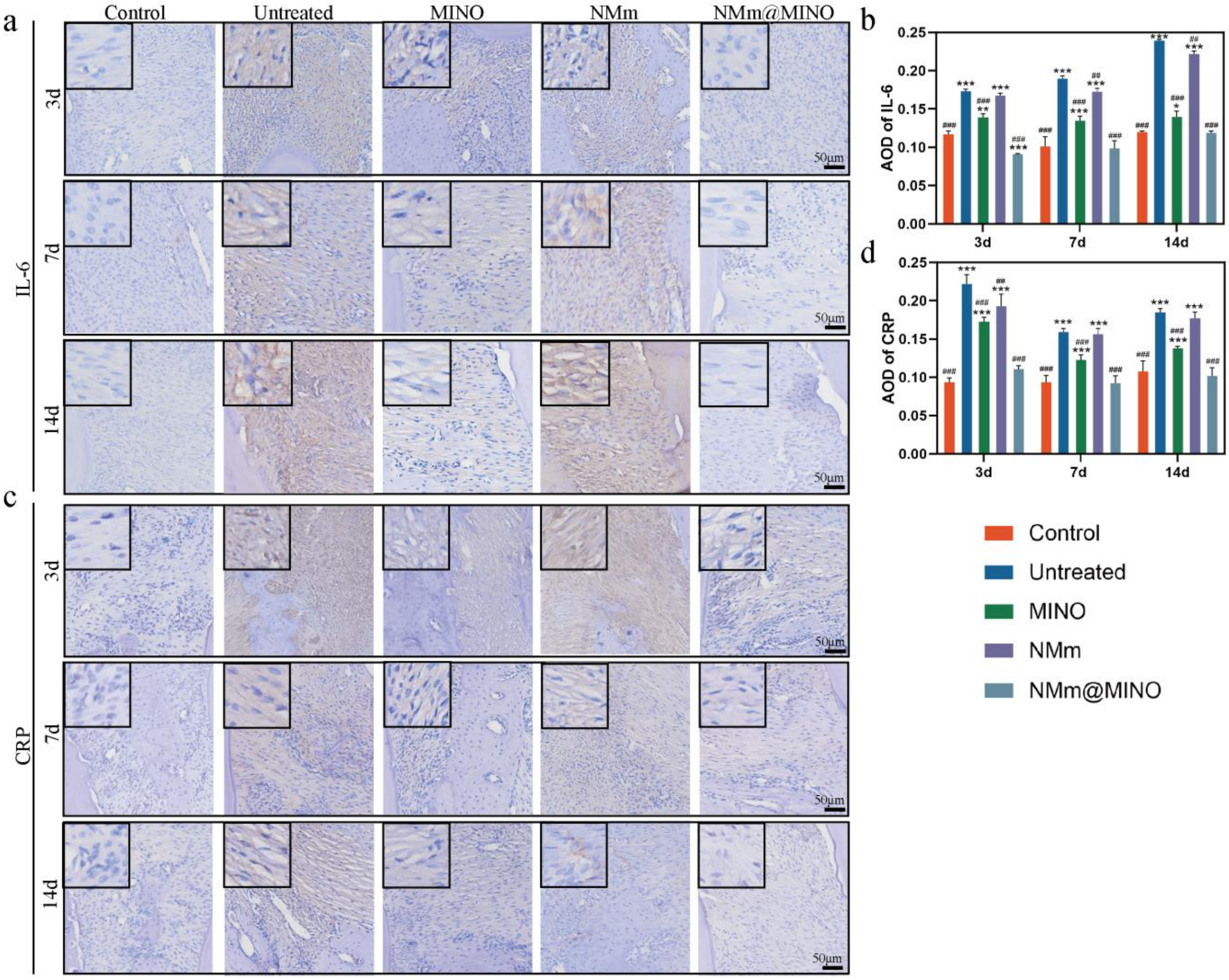

Fig. S23 Anti-inflammatory effect of NMm@MINO *in vivo*. **a** IHC staining of IL-6 in the periodontal tissue. Scale bar = 50 μm. **b** Quantitative analysis of IL-6 expression via average optical density value. **c** IHC staining of CRP in the periodontal tissue. Scale bar = 50 μm. **d** Quantitative analysis of CRP expression via average optical density value. Data are presented as mean ± SD, n = 3, compared with Control group, * $p < 0.05$, ** $p < 0.01$, *** $p < 0.001$; compared with Untreated group, $^{\#}$ $p < 0.05$, $^{\#\#}$ $p < 0.01$, $^{\#\#\#}$ $p < 0.001$.

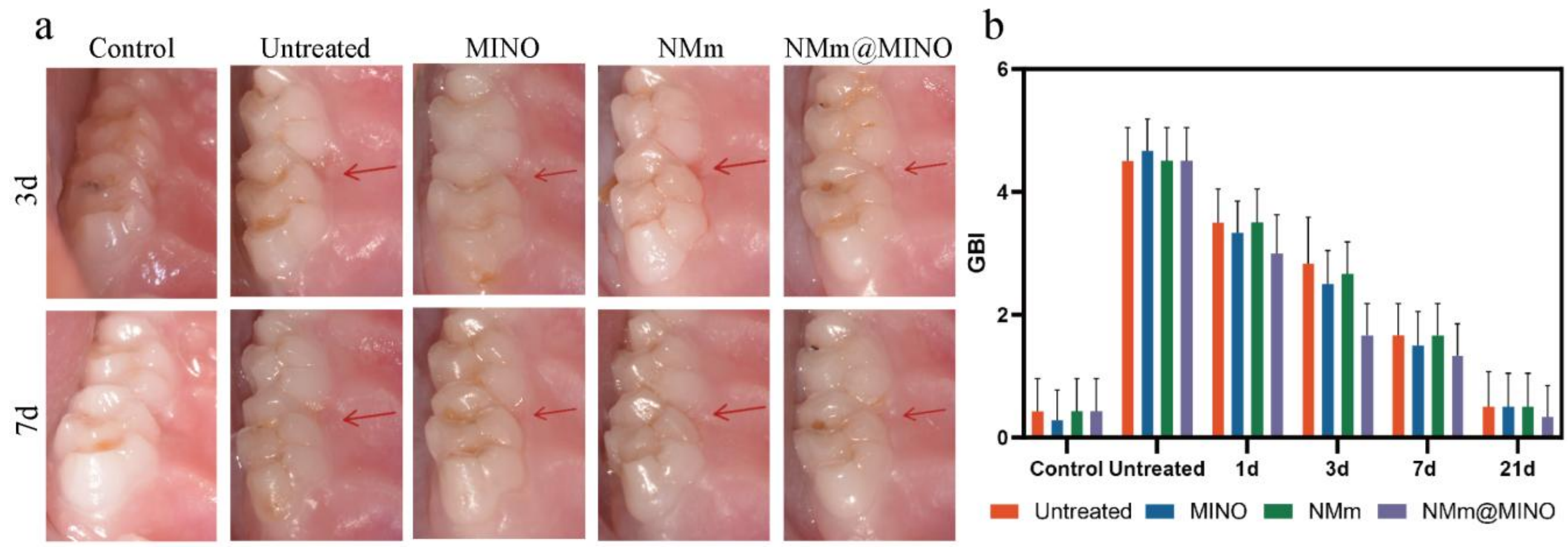

Fig. S24 Gum bleeding index (GBI) of the indicated groups. (**a**) Representative clinical images of gingival bleeding at 3 d and 7 d; the red arrows indicate the sites of gingival bleeding, which were used to evaluate the bleeding status of each group; (**b**) Quantitative analysis of GBI. Data are presented as mean ± SD, n = 3.

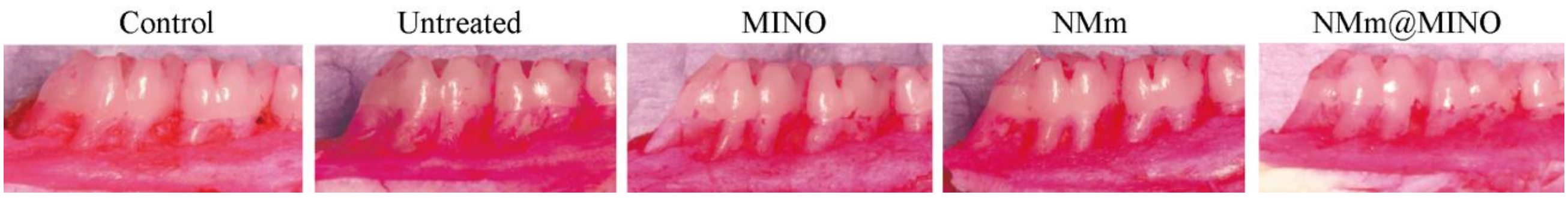

Fig. S25 Plaque staining for periodontal tissue at 3 days.

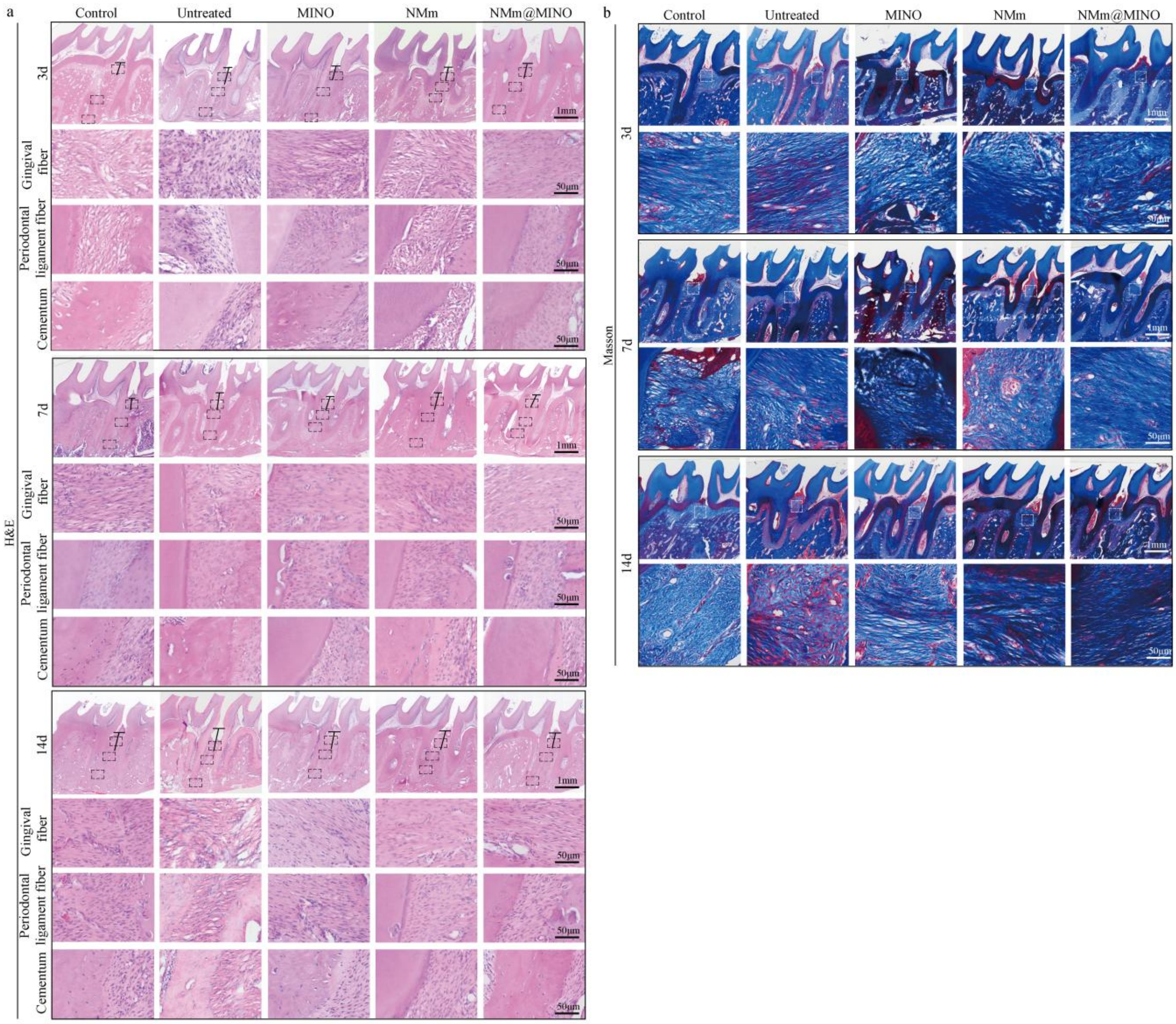

Fig. S26 Soft tissue regeneration effect of NMm@MINO *in vivo*. **a** H&E staining. The black dashed boxes indicate the magnified local views of gingival fibers, periodontal ligament fibers and cementum. Scale bar = 1 mm (first row); 50 µm (second to fourth row). **b** Masson staining. The white dashed boxes represent the magnified local views of gingival fibers. Scale bar = 1 mm (first row); 50 µm (second to fourth row).

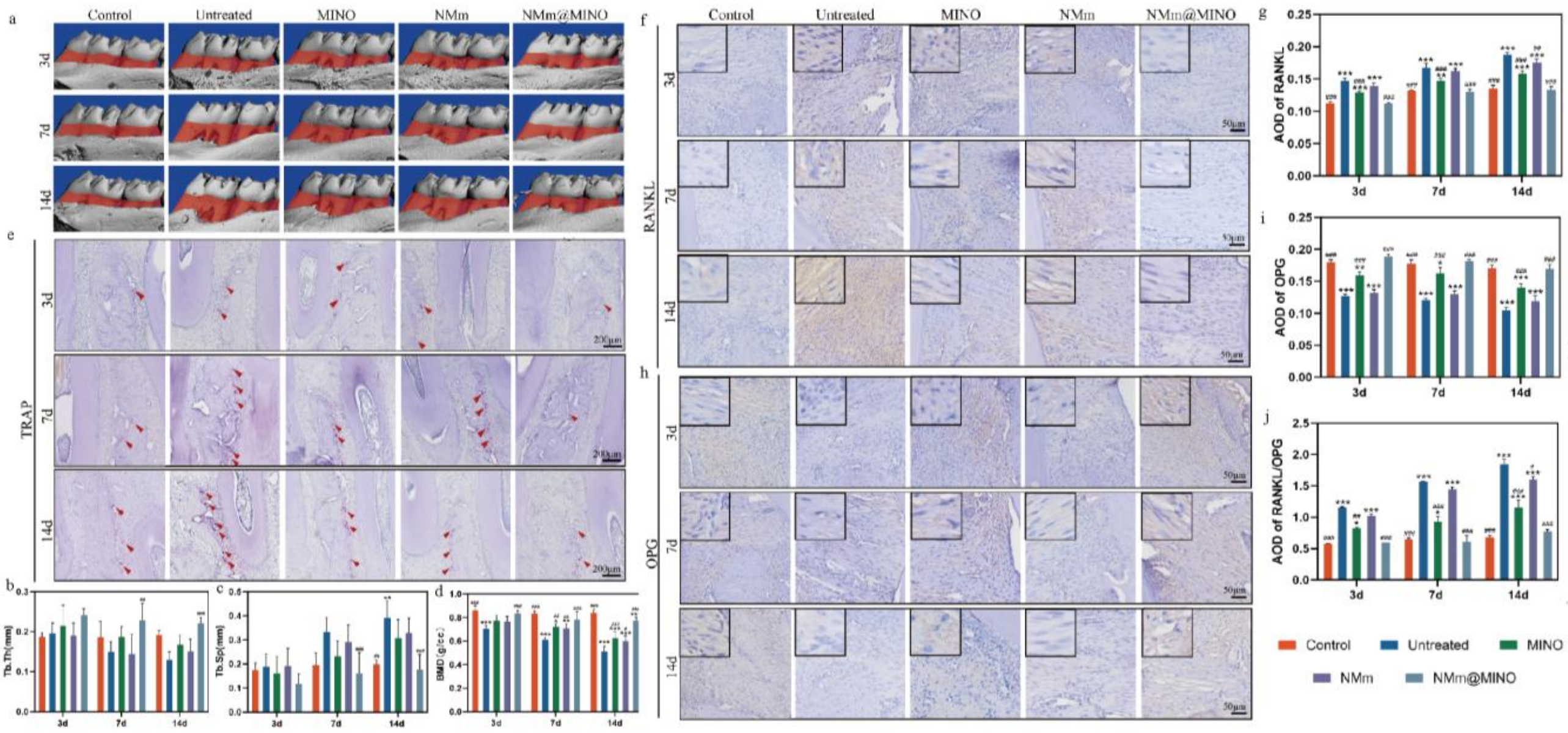

Fig. S27 Effects of NMm@MINO on osteogenic and osteoclastic functions *in vivo*. **a** 3D reconstructed digitized images of the maxillary first and second molars analyzed by micro-CT. The red part displayed the exposure of root. **b** Quantitative analysis of Tb.Th. **c** Quantitative analysis of Tb.Sp. **d** Quantitative analysis of BMD. **e** TRAP staining. Scale bar = 200 μm. **f** IHC staining of RANKL in the periodontal tissue. Scale bar = 50 μm. **g** Quantitative analysis of RANKL expression via average optical density value. **h** IHC staining of OPG in the periodontal tissue. Scale bar = 50 μm. **i** Quantitative analysis of OPG expression via average optical density value. **j** Quantitative analysis of RANKL/OPG expression via average optical density value. Data are presented as mean ± SD, n = 3, compared with Control group, * $p < 0.05$, ** $p < 0.01$, *** $p < 0.001$; compared with Untreated group, $^{\#}$ $p < 0.05$, $^{\#\#}$ $p < 0.01$, $^{\#\#\#}$ $p < 0.001$.

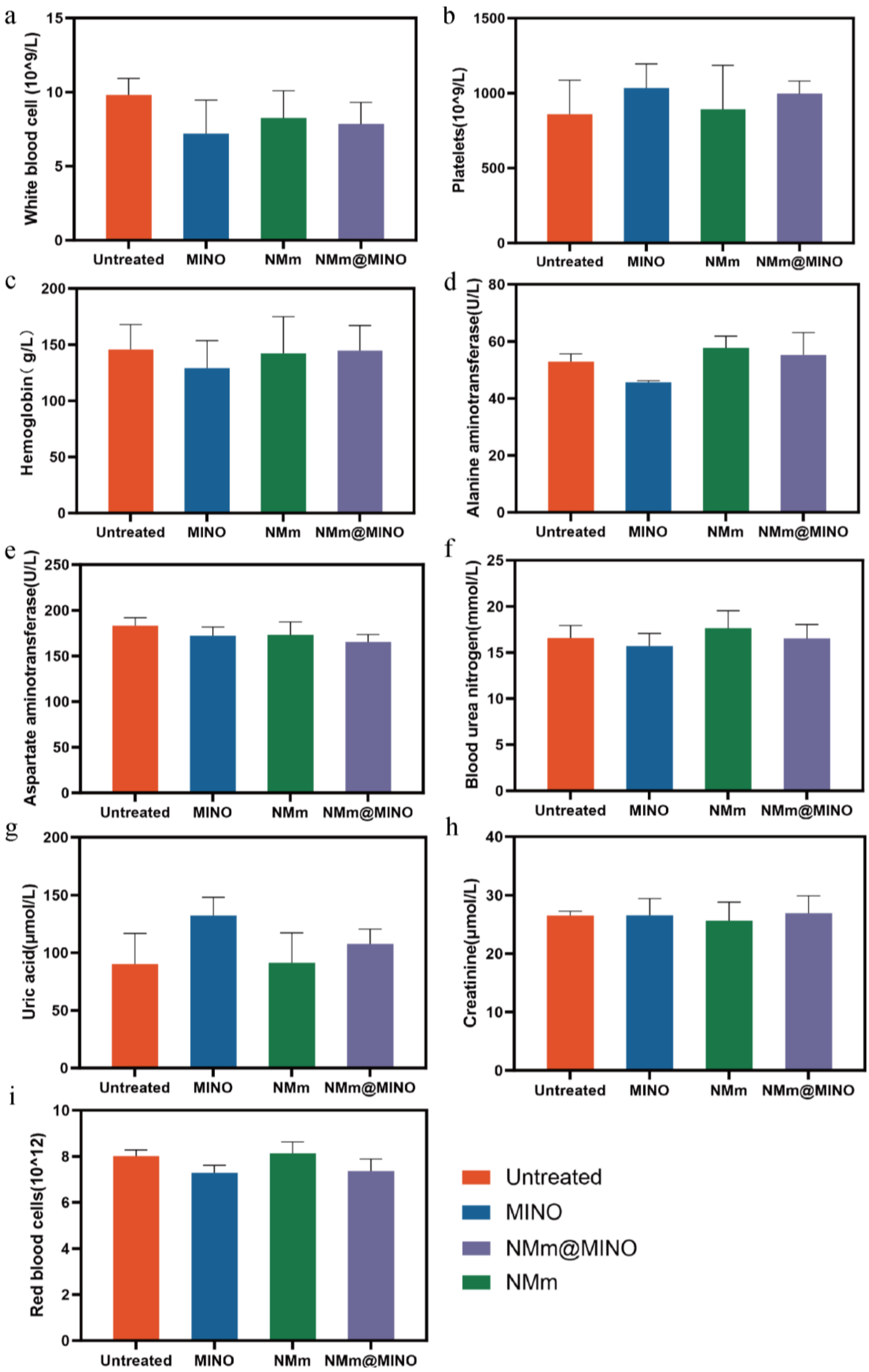

Fig. S28 Hematological examination analysis. Data are presented as mean ± SD, n = 3.

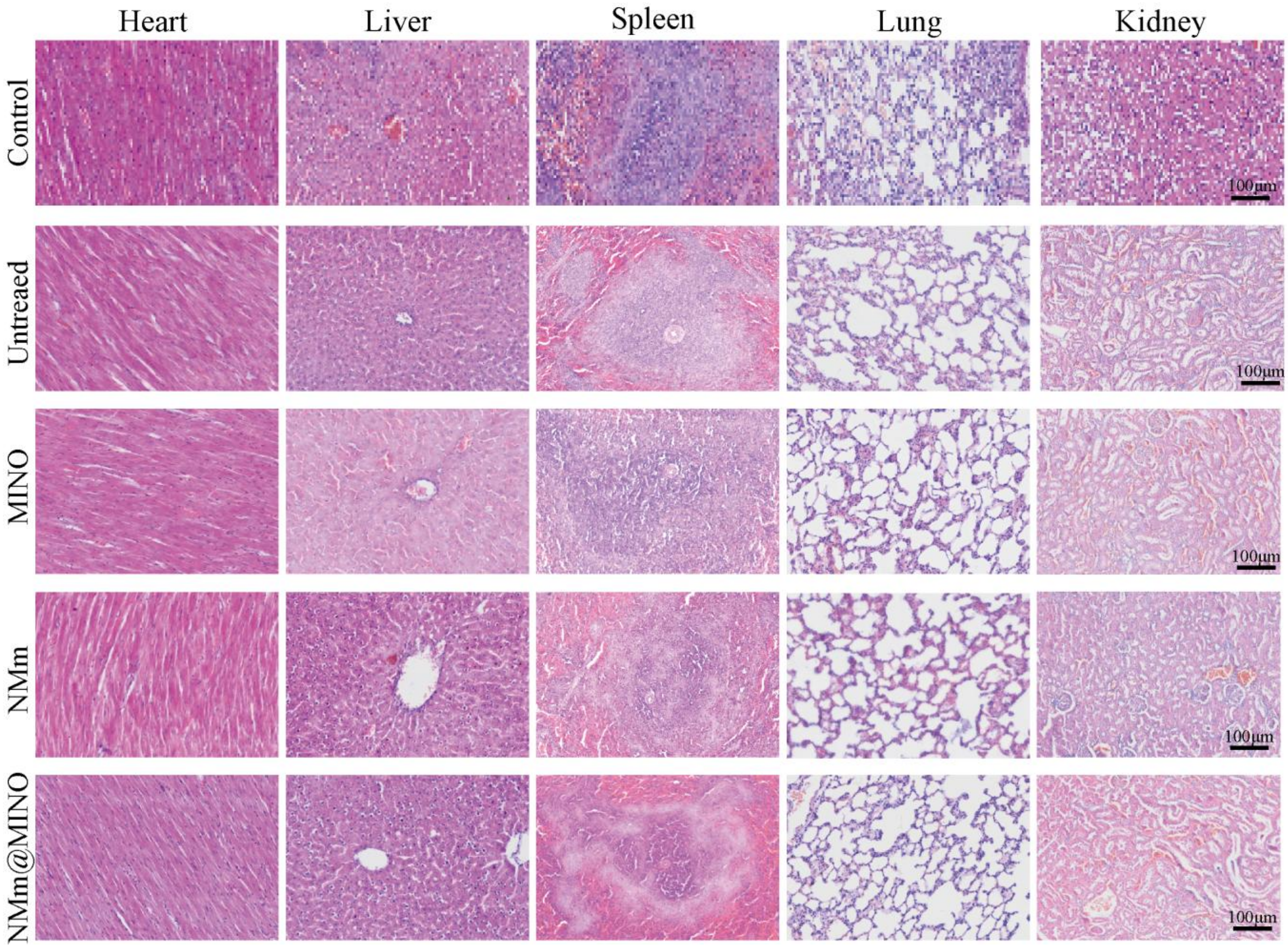

Fig. S29 Biosafety assessment of NMm@MINO *in vivo*. Scale bar = 100 μm.

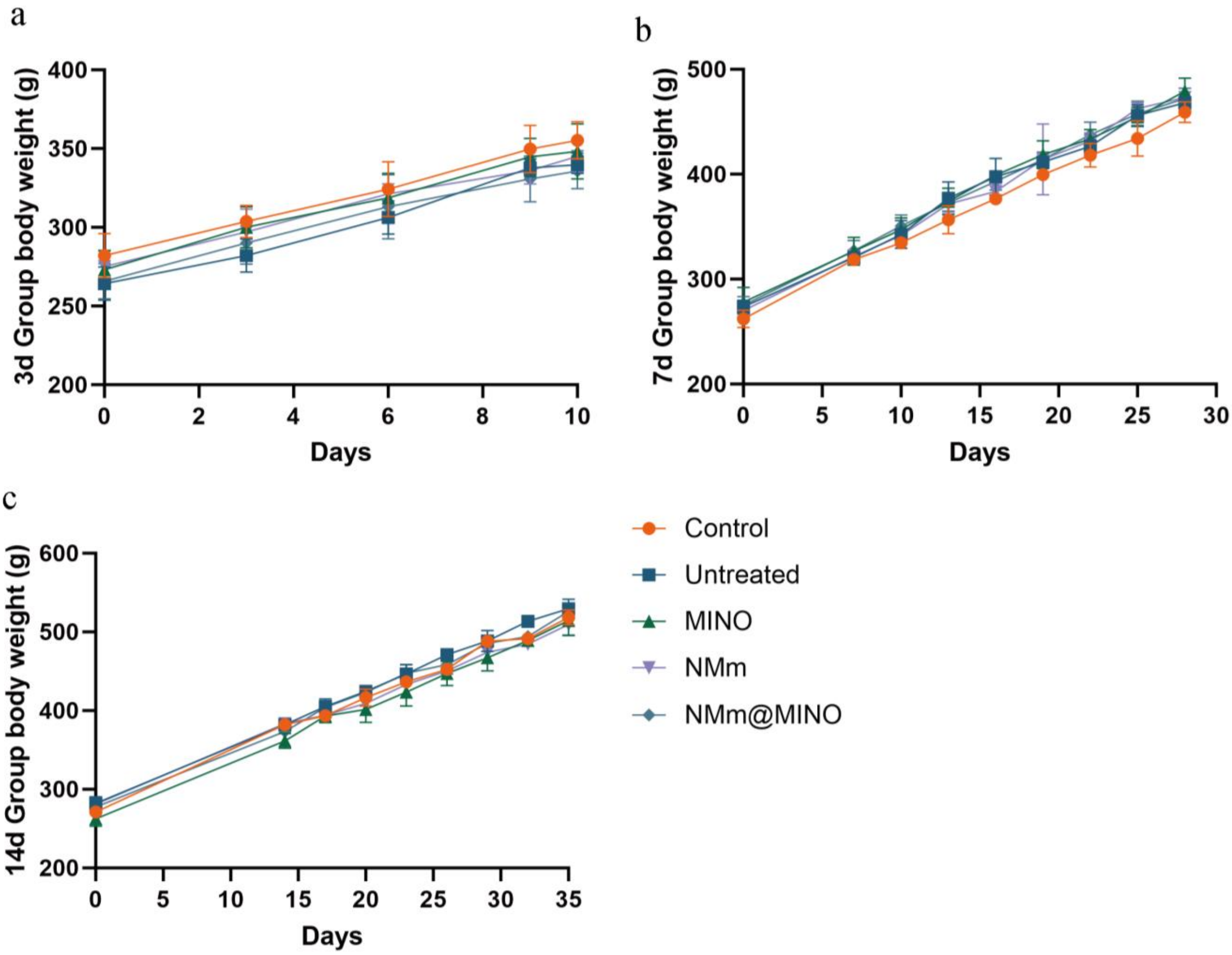

Fig. S30 Weight changes in rats of different groups. (**a**) 3d group, (**b**) 7d group, (**c**) 14d group. Data are presented as mean ± SD, n = 3.
